# Volatilomes of human infection

**DOI:** 10.1007/s00216-023-04986-z

**Published:** 2023-10-16

**Authors:** Shane Fitzgerald, Linda Holland, Waqar Ahmed, Birgit Piechulla, Stephen J. Fowler, Aoife Morrin

**Affiliations:** 1 SFI Insight Centre for Data Analytics, School of Chemical Sciences, National Centre for Sensor Research, Dublin City University, Dublin, Ireland; 2 School of Biotechnology, Dublin City University, Dublin, Ireland; 3 Division of Immunology, Immunity to Infection and Respiratory Medicine, School of Biological Sciences, The University of Manchester, Manchester, UK; 4 Institute of Biological Sciences, University of Rostock, Rostock, Germany; 5 Respiratory Medicine, Manchester Academic Health Science Centre, Manchester University NHS Foundation Trust, Manchester, UK

**Keywords:** Microbial volatiles, Clinical, Metabolic pathways, Gas chromatography, Mass spectrometry

## Abstract

The human volatilome comprises a vast mixture of volatile emissions produced by the human body and its microbiomes. Following infection, the human volatilome undergoes significant shifts, and presents a unique medium for non-invasive biomarker discovery. In this review, we examine how the onset of infection impacts the production of volatile metabolites that reflects dysbiosis by pathogenic microbes. We describe key analytical workflows applied across both microbial and clinical volatilomics and emphasize the value in linking microbial studies to clinical investigations to robustly elucidate the metabolic species and pathways leading to the observed volatile signatures. We review the current state of the art across microbial and clinical volatilomics, outlining common objectives and successes of microbial-clinical volatilomic workflows. Finally, we propose key challenges, as well as our perspectives on emerging opportunities for developing clinically useful and targeted workflows that could significantly enhance and expedite current practices in infection diagnosis and monitoring.

**Graphical Abstract:**

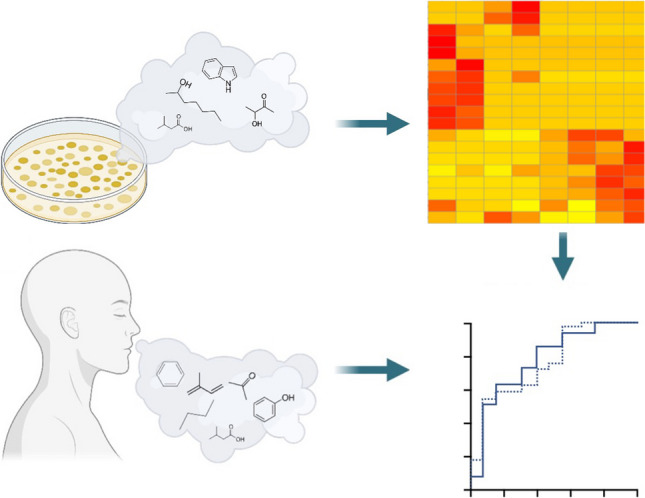

**Supplementary Information:**

The online version contains supplementary material available at 10.1007/s00216-023-04986-z.

## Introduction

The growing threat of infectious diseases has proven to be a significant burden on public health and economies [1]. On a global level, the spread of disease has been accelerated by rising populations worldwide, increased travel and trade [2], increased human interference with nature and wildlife [3, 4], and the overuse of antibiotics [5, 6]. A major factor in the increase in severity and mortality rates of infectious diseases is the rapid spread of antimicrobial resistance [6]. Despite recent developments in molecular diagnostics, these techniques are expensive to employ, are highly specialised, and are not universally accessible. Endogenous blood biomarkers such as white blood cell count, erythrocyte sedimentation rate, C-reactive protein, and procalcitonin are regularly used as indicators of the host response to infection and therefore a relative measure of infection severity [7, 8]. However, these markers are highly sensitive to comorbidities and can be unreliable for the discrimination of inflammation caused by or independent of infection. Chest X-rays and microbiology culture methods are also common in identifying infections, but subject to long waiting times and low accuracy. Development of innovative techniques to accurately target specific volatile patterns and/or biomarkers of infectious diseases would provide a rapid, cost-effective, and non-invasive alternative to conventional methods.

Microbes emit volatile organic compounds (VOCs) as products of primary and secondary metabolic pathways. Across microbial kingdoms, the metabolism of sugars, lipids, amino acids, sulfur- and nitrogen-containing compounds, and aromatic compounds and the subsequent metabolism of those products give rise to thousands of VOCs [9–11]. As such the microbial volatilome is defined as the holistic collection of VOCs produced by microbes. Analysing pure cultures in varying growth conditions allows accessory and core VOCs to be characterised. These techniques have been recently highlighted as a potential route towards discovery of volatile biomarkers of infection. Discriminatory volatilomic patterns and compounds have been associated with infectious diseases and maladies such as pneumonia, tuberculosis, COVID-19, and wounds. Identifying VOCs from the growth of specific pathogens in vitro provides an insight into altered metabolism during infection.

Wide variation in experimental and instrumental techniques used across volatilomics has essentially slowed progress towards clinical biomarker validation, and independent validation studies producing multi-dimensional data struggle to find common ground. However, the field and literature-base are rapidly progressing, with reviews [9, 12, 13], books [10], and online databases [14] increasing confidence in specific VOCs being validated towards clinical translation. However, it is critical to move towards standardised methods of sample collection, pre-analytical preparation, and analysis. Such a move will reduce biases and improve reproducibility of results across studies and ultimately lay the foundations for future clinical applications of volatilomics.

The aim of this review is to highlight the clinical potential of microbial VOCs for future diagnostics. An initial broad discussion of the fundamental pathways from which these metabolites are generated will precede a discussion of key topics: sampling and analysis tools used in microbial and clinical volatilomics; translation of in vitro microbial volatilomics into clinical volatilomics; the challenges and emerging directions of volatilomics.

## Metabolic pathways involved in microbial volatilomes

### Primary metabolism

Primary metabolic products are derived from pathways such as glycolysis, fermentation, the tricarboxylic acid (TCA) cycle, and various electron transport chains [15]. In primary metabolism, carbon is derived from organic material (chemoorganotrophy), atmospheric CO_2_, or other inorganic molecules (chemolithotrophy), to generate energy in the form of adenosine triphosphate (ATP). When oxygen is utilised as the final electron acceptor, many microbes can oxidize glucose completely to the inorganic volatile CO_2_ to generate a maximum amount of ATP. The steps involved in the complete oxidation of glucose give rise to a variety of highly volatile primary metabolites such as acetic acid, acetoin, and acetaldehyde. Under oxygen-limited conditions, lower growth rates are characterised by the use of exogenous SO_4_^2−^, NO_3_^−^, or CO_3_^2−^ as alternative electron acceptors to generate comparatively less ATP [16]. In addition to these key alternative electron acceptors, some compounds are used as electron sinks; e.g., dimethyl sulfoxide (DMSO) is reduced to dimethyl sulfide, and trimethyl amine-N-oxide (TMAO) to trimethylamine [11]. By utilising all available electron acceptors, microbes can survive longer in different environments including low-oxygen environments. Without external electron acceptors, microbes can initiate fermentation and transfer the electrons to internal acceptors. This process is primarily characterised by the production of ethanol but can lead to the emission of a variety of alcohols, fatty acids, ketones, aldehydes, and other chemical classes. Certain bacteria and fungi can excrete fermentation products even in the presence of oxygen and other high potential electron acceptors. This seemingly less efficient and wasteful phenomenon is known as overflow metabolism [17]. It is characterised by the excretion of acetate (the “acetate switch” [18]), which can occur aerobically when growth on excess glucose inhibits respiration (i.e. the Crabtree effect [19]). This can also be due to an enzyme of the tricarboxylic acid cycle being missing or repressed [20].

### Secondary metabolism

Primary metabolic intermediates and products can be further metabolised into large numbers of secondary metabolites [9, 12]. Secondary metabolism typically occurs during the stationary phase of microbial growth as microbes begin to exhaust all available primary substrates. The metabolites generated through secondary metabolism are highly diverse containing a variety of chemical classes such as terpenes, aromatic compounds, sulfurous and nitrogenous compounds, and fatty acid derivatives. Unlike primary metabolites which are highly common across the microbial kingdom, secondary metabolites are potentially species-specific and mediate various host-microbe and microbe-microbe interactions [11].

### Short-chain and aromatic amino acid metabolism

Amino acids can be derived from microbial breakdown of host proteins during tissue necrosis. The metabolism of these amino acids is a key source of volatile metabolites. Leucine is a short-chain amino acid that is readily catabolised by microbes. VOCs such as 3-methyl-1-butanol (isoamyl alcohol), 3-methylbutyric acid (isovaleric acid), 2-methylbutyric acid, and 3-methyl-1-butanol acetate (isoamyl acetate) [21] result from this catabolism. The shikimate pathway is a seven-step metabolic pathway linking the metabolism of sugars to the biosynthesis of aromatic compounds [22]. Key amino acids such as tryptophan, tyrosine, and phenylalanine are commonly produced using this pathway. Phenylethyl alcohol [23], indole [24], and 2-aminoacetophenone [25] are examples of downstream metabolites produced at various stages of this pathway [17]. In the context of infection, some microbes form biofilms in vivo as a survival mechanism. In *Candida* spp., the upregulation of amino acids during biofilm development [26] can shift cell metabolism in favour of amino acids over sugars. A recent work has supported this by demonstrating the volatile output of amino acid–derived metabolites increases as *Candida* spp. biofilms mature [27]. Sulfur-containing volatiles are primarily derived from the oxidation of methanethiol. Methanethiol is associated with decaying biomass. It spontaneously dimerises in air to form dimethyl disulfide [28] but also can be oxidised metabolically by microbial oxidase enzymes [29] to form sulfides, disulfides, and trisulfides. In living systems, sulfur-containing volatile compounds can also be generated through the metabolism of sulfur-containing amino acids cysteine and methionine [30]. These pathways are particularly relevant to volatilomes of gastrointestinal (GI)-associated pathogens such as *Helicobacter pylori* [31] and *Clostridium difficile* [32], which both have volatile sulfurous-compound profiles.

### Fatty acid biosynthesis and degradation

During human infections, many pathogens efficiently metabolise host fats [33, 34] to compensate for low availability of sugars and amino acids. Fatty acids are typically synthesised by condensation reaction between acetyl CoA and malonyl CoA before undergoing several stages of chain elongation. Microbes are capable of utilising a variety of starter units — other than acetyl CoA — for this particular reaction and results in vast diversity in compounds produced. Decarboxylation of intermediate compounds — produced as biproducts of each chain extension cycle — leads to the generation of various alkanes, 1-alkenes, and methyl ketones [21]. Microbial breakdown of fatty acids involves a ß-oxidation reaction that ultimately ends with the acetyl CoA starter unit [35] liberating a variety of volatile ketones, aldehydes, acids, and alcohols of varying chain lengths at each degradative step. Hydrolysis and reduction reactions of metabolic intermediates also give rise to a variety of compounds such as acids, 1-alcohols, and aldehydes. This pathway is utilised by many bacteria, including infection-causing pathogens. For example, 1-undecene was detected in breath of patients with *Acinetobacter baumannii–*positive ventilator-associated pneumonia (VAP) [36].

## Analytical techniques used across microbial and clinical volatilomics

Microbial culture headspace (HS) experiments are typically carried out in glass HS vials [16, 36–41]. Alternative HS collection vessels such as cell culture well plates may be used dependent on the experimental objectives being pursued [27, 42]. Table 1 provides several experimental systems used across microbial volatilomics investigations. Clinical translation has primarily been focused on breath research, likely due to the relative ease of non-invasive sample collection. Sampling and analysis of breath samples has been comprehensively reviewed [43, 44]. A significant development in breath sample collection was catalysed by the Breathe-Free Consortium in the standardisation of an open-source breath sampling device (ReCIVA, Owlstone Medical) [45]. Other human matrices such as sputum, urine, blood, faeces, sperm, sweat, and wound tissue/fluid also provide opportunities for clinical volatilomic research, examples of which can also be seen in Table 1. Large reference libraries available with techniques such as GC–MS support broad untargeted screening of compounds (e.g. NIST mass spectral library) are commonly used to identify VOCs. GC–MS utilises a temperature ramp across narrow open-tubular columns to allow high-resolution analysis of trace analytes of varying volatilities. Compounds eluting from the GC column are then fragmented in a highly reproducible process via electron ionisation before passing to the MS detector. Electron ionisation of compounds allows the construction of vast reference libraries such as NIST due to the reproducibility of the process. However, the diversity of molecules that can be analysed is limited in conventional one-dimensional GC–MS due to its individual use of either a polar or non-polar column and low-resolution mass analyser. The range of compounds that can be accurately analysed per run as well as chromatographic resolution can be significantly enhanced using two-dimensional GC–MS (GC × GC)(46). GC × GC incorporates two separation stages using two columns with different retention mechanisms that are connected to each other via a modulator that traps compounds eluting from the first column before rapidly injecting them into the second column. As discussed later in this review, high-resolution mass analysers such as time of flight (TOFs) and orbitraps also expand the untargeted screening capabilities of GC–MS by accurately revealing the ionic species present in chromatographic peaks. Untargeted whole volatilome profiling allows a wide range of volatile compounds to be identified and assessed for their discriminative impact. Discriminative compounds identified in untargeted clinical analyses must be subsequently targeted, quantified, and validated before being proposed as potential biomarkers of disease. However, wide variation in experimental techniques used across microbial and clinical volatilomics limits cross-study comparisons of data and ultimately blocks external validation of results. Several studies use direct mass spectrometric techniques which have limitations in the range of compounds which can be analysed. That said, routine workflows have been implemented for techniques such as selected ion flow tube (SIFT)-MS [47], as shown in Fig. 2.

**Table 1:** Overview of frequently used sampling and analytical methods in microbial and clinical volatilomics

Technique	Benefits	Limitations	Microbial volatilomics	Clinical volatilomics
SPME fibre	• Diverse analyte range • Easily automated with GC–MS • Adaptable sampling methods e.g. cutaneous (skin, wounds)	• Semi-quantitative challenging • Storage of sample • Method optimisation required	• HS vial [36–41] • Culture vessel[61] • Glass enclosed 6-well plate[27]	• Skin [27, 62] • Breath [63, 64] • Wounds[65–67] • Faeces[68]
Sorption tube/needle trap	• Diverse analyte range •Easily automated with GC–MS • Suitable for breath analysis • Can be stored and transported after sampling	• Method optimisation required • Not suitable for online analysis • Water retention issues	• HS vial [69, 70] • Culture vessel [71]	• Breath [36, 37, 72] • Faeces [73]
Gas collection bag/direct syringe	• Simple collection procedure	• Pre-concentration required for non-sorbent syringe methods • Cannot be stored long term	• HS vial [74, 75]	• Breath [54, 76]
Real-time analysis (SIFT-, PTR-, SESI-MS)	• Quantification • Real-time • Highly sensitive (LOD < 1 ppb) • Targeted analysis • Low cost per sample	• Challenging for screening of unknowns • Limited VOC profiles • High instrument cost	• HS vial [47, 77–79] • Biofilm assay [80] • Culture vessel [81]	• Skin [82] • Breath [55, 78–80]

### Sorption-based sampling with gas chromatography-mass spectrometry

Thermal desorption sampling using sorption tubes and solid phase micro-extraction (SPME) fibers are commonplace in microbial and clinical volatilomics. Less common thermal desorption sampling such as stir-bar sorptive extraction (SBSE) has also been effectively applied for in vitro VOC sampling [48]. These sampling approaches are all compatible with GC–MS. Sorption tubes are typically stainless steel or glass tubes, or needle trap devices that are packed with a single or combination of sorbent material beds (e.g. porous polymers, graphitised carbon, silica gels) to expand the range of analytes that can be trapped [49]. The sorbent materials used for a particular experiment must be carefully considered and optimised against other materials as extraction yields, selectivity, and reproducibility are significantly associated with specific sorbents [50].

This technique has proven to be well suited to clinical breath studies (Table 1) where the breath sample is typically collected into sorption tubes for offline analysis. SPME utilises a chemically enhanced silica fiber (stationary phase) consisting of various phases to capture a wide range of analytes via an equilibrium extraction mechanism. During sampling, the SPME fiber is exposed to an enclosed sample HS where — at an experimentally determined time point — a partitioning equilibrium between the sample matrix and fiber is reached. At this point, the fiber can be retracted from the sample HS and injected into the GC–MS. Each phase has unique pore sizes and polarity characteristics that allow the retention of both small volatile non-polar compounds and larger less volatile, polar compounds [51]. Research into advanced fiber coatings is being carried out to expand on the ranges of compounds that can be extracted by SPME [52]. SBSE relies on aseptic stir bars that can be coated with sorbent materials such as polydimethylsiloxane/ethylene glycol and exposed to VOCs in an enclosed system before being thermally desorbed and analysed using GC–MS [48].

### Online and near-patient volatilomics profiling

**Fig. 1:**
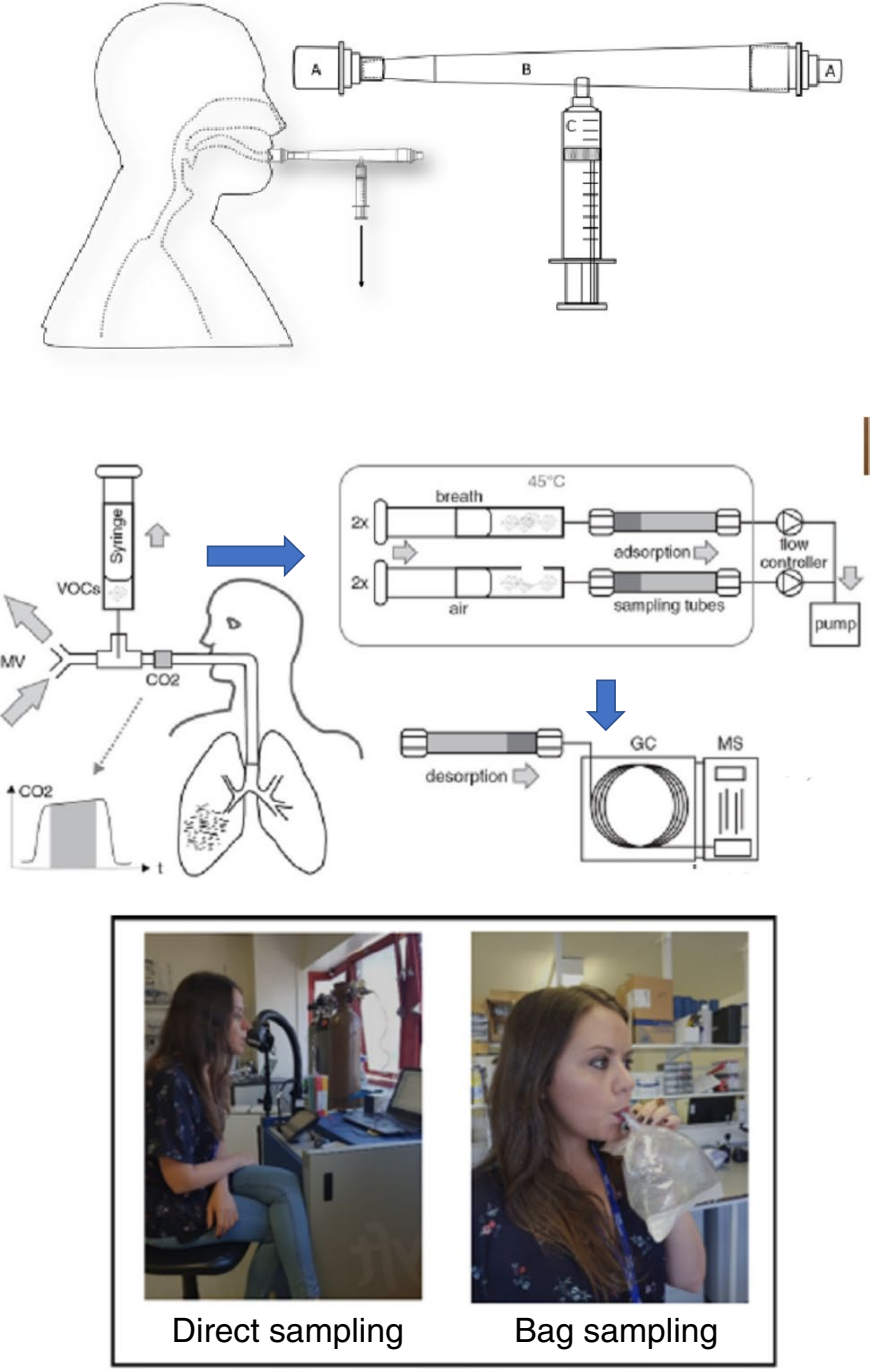
Frequently used in vivo volatilomic breath sampling techniques. *Top*: direct syringe sampling apparatus employed by Ruszkiewicz et al. [54] for GC–MS breath profiling of COVID-19 patients. *Middle*: syringe-sorption tube sampling workflow for breath profiling of ventilator associated pneumonia (VAP) patients followed by GC–MS analysis [37]. *Bottom*: direct breath sampling and bag sampling of breath for direct quantification of target analytes by SIFT-MS [55]

Online and near-patient analyses are a promising prospect for volatilomic profiling investigations. The advantages of direct detection methods (Table 1) include real-time targeted analysis, absolute quantification, and high sensitivity. Samples are analysed immediately on collection, which eliminates potential errors that could arise as a result of sample storage and transportation [53]. Near-patient techniques as demonstrated by Ruszkiewicz et al. [54] (Fig. 1) involve sampling from the patient and analysing on a nearby instrument such as ion mobility spectrometry. This point-of-care analysis is ideal for immediate results for example in emergency triage. Frequently used direct techniques in volatilomics include proton transfer reaction (PTR)-MS, SIFT-MS, ion molecule reaction (IMR)-MS, ion mobility spectrometry (IMS)-MS, and secondary electrospray ionisation (SESI)-MS. Among these techniques, the most frequently employed in volatilomics are SIFT-MS [55] and PTR-MS [56] as these have the advantage of analysing very volatile compounds such as hydrogen cyanide from cystic fibrosis (CF) patients with lung infection or dynamic change in breath VOC concentrations [57, 58]. Workflows based on these techniques have been adapted for both microbial and clinical volatilomics studies (Table 1). However, due to limited reference libraries, these methods are not currently suitable for holistic volatilome profiling. It is important to note the use of e-nose technology for near-patient real-time VOC measurements; however, as this technique is limited in its molecular identification capabilities, the discussion of its mechanics and applications is outside the scope of this review and can be found elsewhere [59, 60].

## Microbial and clinical volatilomics

**Fig. 2:**
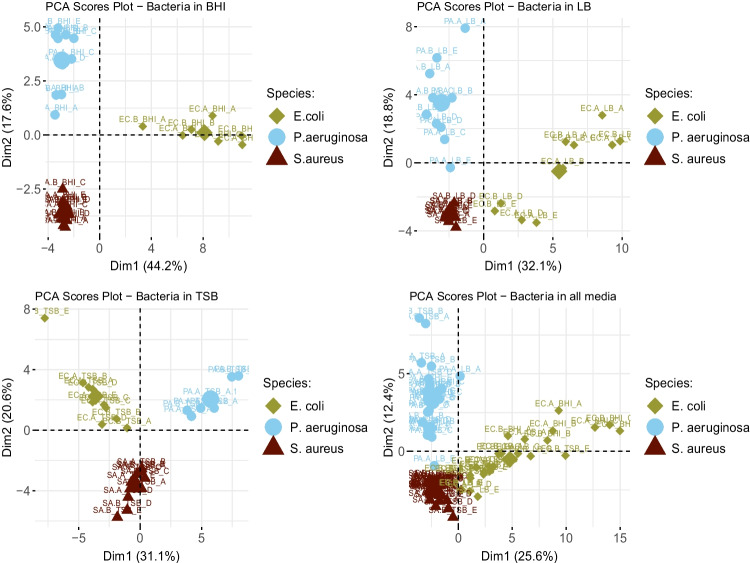
Principal component analysis (PCA) is frequently used in microbial and clinical volatilomics to visualise differences between study groups (e.g. different patient groups or microbial species). In PCA score plots shown here, clear discrimination between three clinically prevalent bacterial pathogens is observed across different nutritional growth media (BHI, brain heart infusion; LB, lysogeny broth; TSB, tryptic soy broth). Despite differences, species retain key metabolic activity across varying nutritional environments and emit many of the same characteristic metabolites — known as the core volatilome [40]

**Fig. 3:**
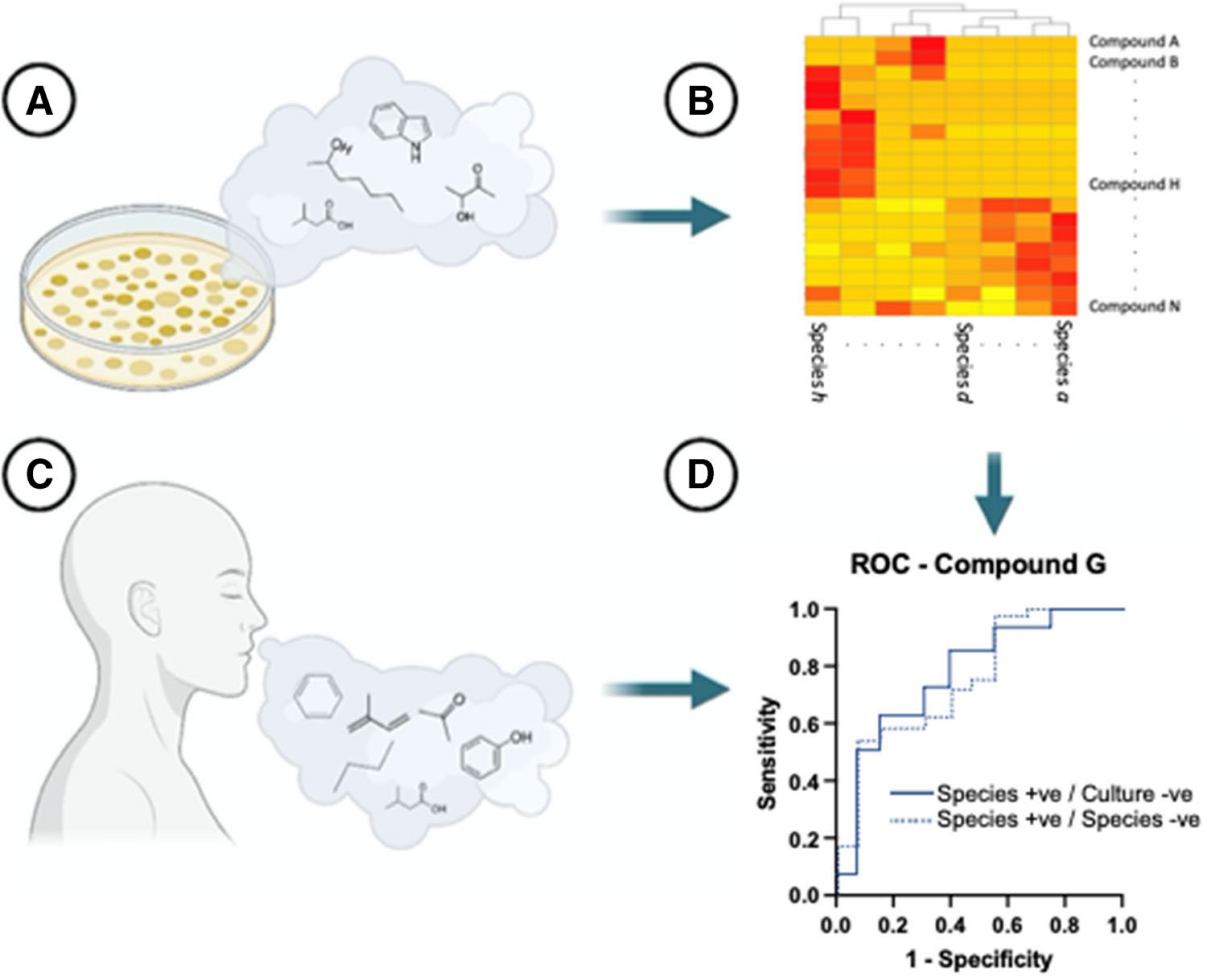
Developing a bottom-up targeted clinical volatilomics workflow for pathogen detection. **A** Pathogenic microbes/human cell lines are cultured under varying environmental conditions from which VOCs are collected and analysed, e.g. using GC–MS. **B** Following peak identification and validation, VOC data are analysed using untargeted techniques such as hierarchical clustering to identify highly discriminant and characteristic pathogen-associated VOCs. **C** Clinical patient sample (e.g. breath) is collected and analysed. **D** In vitro data used to establish a diagnostic model for predicting presence of pathogens in patient samples. For example, receiver operating characteristic (ROC) curves assess the sensitivity indicated by the area under the curve (AUC) of target molecules present in patient samples for identified pathogens

Characterisation of microbial volatilomes involves compiling data from experiments in different environments, growth phases, and experimental systems. The progressive development of the mVOC database has enabled the broad profiling of metabolites emitted from many microbes [83]. Broadly speaking, microbial volatilomic studies have aimed at comprehensively characterising pathogen volatilomes; discriminating pathogen volatilomes from each other (Fig. 2 and previous literature [38, 40, 84]); identifying the chemical origin of novel secondary microbial metabolites [85]; and testing growth conditions that affect metabolite production [86]. Pathogen volatilomes have been screened in vitro to identify potential markers of disease for clinical investigations [36, 37, 39, 87]. Conditions such as growth phase of cells, nutritional media, and temperature influence the resulting volatile emissions from microbial cells [88]. Therefore, the results from these studies cannot be directly translated to clinical applications and careful consideration of these factors is required before planning clinical volatilomic experiments. However, microbial metabolites that have been previously detected in the HS of pure cultures have also been detected in samples taken in humans and animals infected with these pathogens (Fig. 3). These occurrences have been limited to the detection of bacterial and fungal pathogens. Respiratory viral infection has previously demonstrated discriminatory volatilomic shifts in human cells in vitro [89, 90]; however, detecting volatiles specific to a virus is unlikely as they do not produce their own metabolites and instead differential VOCs would originate from altered host metabolism [91].

### Bacterial and fungal lung infection

Several bacterial and fungal infections have been investigated including in patients with CF and VAP. Breath volatile analysis of intensive care patients on mechanical ventilation showed that those with VAP or ventilator-associated lower respiratory tract infection could be distinguished from those without infection [92–94].

Semi-targeted investigations have detected *Escherichia coli*, *Staphylococcus aureus*, *Candida albicans* [37], *Acinetobacter baumannii* [36], and *Aspergillus fumigatus* [95] in the breath of intensive care patients. In these studies, the compounds detected in both in vitro and breath screens included primary, fatty acid, and amino acid metabolites such as acetic acid, 3-methylbutanal, indole, and 1-undecene. The detection of 1-undecene in *A. baumannii* highlights the potential for detecting *Pseudomonas aeruginosa* from clinical samples, as this metabolite is a core component of its volatilome [40]. Most recently, following preliminary in vitro screening of multiple VAP-associated pathogens, 3-methylbutanal and 3-methylbutyric acid were identified in breath of mechanically ventilated patients as strong predictors of *S. aureus–*associated infection [87]. Similarly, studies investigated breath profiling of CF patients with chronic *P. aeruginosa* [96–98] and *S. aureus* [99] infection were able to classify infected and non-infected patients.

### Tuberculosis

Preliminary detection of fatty acid–derived hydrocarbons in *Mycobacterium tuberculosis* cultures allowed semi-targeted in vivo investigations in which the breath of subjects with tuberculosis and non-infected subjects were discriminated [39, 100]. Genomic sequencing of *M. tuberculosis* revealed its fatty acid degradation pathways are disproportionately active compared to other pathogens [101]. Animal models have demonstrated that during infection *M. tuberculosis* heavily relies on host lipids for survival [33] — demonstrating potential of fatty acid metabolites as target chemical groups for future pathogen detection. However, results from a limited number of in vitro investigations have also highlighted amino acid metabolism activity in the *M. tuberculosis* volatilome [102–104]. *M. tuberculosis* has been described to effectively utilise amino acids as a primary source of nitrogen in vitro [105] most likely giving rise to aromatic volatile by-products. For complex pathogens such as *M. tuberculosis*, incorporating lipid substrates such as cholesterol into the nutritional media may increase the specificity and clinical applicability of in vitro studies. Significant developments in infection modelling using organ-on-a-chip technology [106] also increase potential applicability of such models for infection volatilomes in the future.

### Wound infection

Prominent wound-associated pathogens include *Staphylococcus* spp., *Proteobacteria* spp., *Streptococcus* spp., and anaerobic bacteria including *Clostridium* spp. [107]. Volatiles collected from the HS of *Proteobacteria* spp. blood cultures included dimethyl disulfide, dimethyl trisulfide, phenol, and indole [65]. The *S. aureus* volatilome is characterised by the emission of isovaleric acid and 3-methylbutanal (leucine degradation), acetic acid, and butyric acid [38, 40]. These acidic and sulfurous compounds, along with phenol, 3-methylbutanal, and indole, were detected from a HS sampling of wound dressings taken from fungating cancer wounds [66, 67]. Differentiation of volatile signatures from swab samples taken from infected and non-infected wounds using e-nose technology has recently been reported [108]. Due to compound identification limitations, metabolites responsible for that difference were not established. While such studies illustrate infection-specific volatilomic differences potentially exist in wounds, MS-based workflows are required to fully elucidate differential VOCs.

### Gastrointestinal and urinary tract infection

*Helicobacter pylori* infection of the gut causes stomach ulcers. The^13^C urea breath test is routinely used to detect *H. pylori* infection. *H. pylori* has also been linked to development of tumorigenesis [109]. Fatty acids in exhaled breath have been linked to potential partitioning of microbial metabolites across the digestive tract and into the airways [110, 111]. Higher abundances of fatty acids, phenols, and aldehydes in the breath of patients with gastric cancer have also been reported [100, 112] compared to healthy controls. These volatilomic shifts cannot be attributed to any specific microbe(s) but they potentially indicate a microbiome shift associated with disease. Another potential route for detecting volatile biomarkers of disease associated with the gut microbiome could be through the analysis of faecal samples to detect clinically important pathogens such as *Clostridium difficile* [32, 73]. Culturing urine samples to detect urinary tract infection (UTI) can be time-consuming and direct VOC profiling may provide an alternative for detection of common UTI pathogens including *E. coli* and *Proteus* species [113].

### Viral infection

Detecting viral pathogens such as SARS-CoV-2 or influenza virus using VOCs is challenging as these pathogens utilise the host metabolic system to support their growth. Therefore, detecting viruses requires a comprehensive understanding of the “healthy” human volatilome so that any deviations from it could be characterised and correlated with specific infections. Respiratory viral infection has been previously shown to cause discriminatory volatilomic shifts in human cells in vitro [90]. From the limited number of COVID-19 breath studies [54, 114–116], there have been discriminatory patterns between patient groups based on shifts in abundance of regular aldehydes heptanal, octanal, and nonanal. These are common components of the human volatilome and they are associated with oxidative stress and potentially indicate inflammatory response, as have been found in viral infection [117, 118]. Increased breath alkane abundance through the breakdown of lipids has also been associated with oxidative stress [119]. A recent example is decane, reported in breath volatilomic studies in patients infected with COVID-19 [116] and rhinovirus (RV)-A16 [89]. However, while the similarities in discriminative VOCs across these diseases highlight the presence of an inflammatory volatilome, they also illustrate the challenge of characterising disease-specific discrimination (Table 2).

**Table 2:** Overview of discriminative VOCs across microbial and clinical volatilomics studies

Compound	Chemical class/metabolism	Sampling	Analysis	In vitro	In vivo
2-Butyl-1-octanol	Alcohol	SPME/sorption tube [36, 120]	GC–MS [36]	*A. baumannii* [36]	VAP [36] Tuberculosis [120]
2,2-Dimethyl 1-propanol	Alcohol	Sorption tube [115]	GC–MS [115]		COVID-19 [115]
Ethanol	Alcohol/ fermentation	Sorption tube [37, 92, 121]	GCMS [37, 92, 121]	*Many*	VAP [37, 92] CF infection [99]
Isopropyl alcohol	Alcohol	Sorption tube [92]	GC–MS [92]		VAP [92]
1-Propanol	Alcohol	Sorption tube [115]	GC–MS [115]		COVID-19 [115]
Acetaldehyde	Aldehyde	Sorption tube [37, 120]	GC–MS [37, 120]	*S. aureus* [37] *C. albicans* [37] *S. pneumoniae* [122] *H. influenzae*[122]	VAP [37] Tuberculosis [120]
Acrolein (propenal)	Aldehyde	Sorption tube [92]	GC–MS [92]		VAP [92]
Ethanal	Aldehyde	Sorption tube [54, 115]	GC–MS [54, 115]		COVID-19 [54, 115]
Heptanal	Aldehyde	Sorption tube [54, 116, 120]	GC–MS [54, 116, 120] SIFT-MS [112]		COVID-19 [54, 116] Tuberculosis [120] Gastric cancer [112]
3-Methylbutanal	Aldehyde	Sorption tube [37, 87, 120] SPME [40, 41]	GC–MS [37, 40, 41, 87]	*S. aureus* [37, 40, 41, 87] *M. tuberculosis* [120]	VAP [37, 87] Tuberculosis [120]
Methylpent-2-enal	Aldehyde		PTR-MS [114]		COVID-19 [114]
Nonanal	Aldehyde	Sorption tube [36]	GC–MS [36] PTR-MS SIFT-MS[112]	*A. baumannii* [36]	VAP [36] COVID-19 [84, 115, 116] Tuberculosis[120] Gastric cancer[112]
Octanal	Aldehyde	Sorption tube [115]	GC–MS [115] SIFT-MS [112]		COVID-19 [54, 115, 116] Gastric cancer [112]
Propanal	Aldehyde	Sorption tube [37]	GC–MS [37]	*S. aureus* [37] *C. albicans* [37]	VAP [37]
Tetradecanal	Aldehyde	Sorption tube [92]	GC–MS [92]	*E. coli* [40, 41]	VAP [92]
Benzaldehyde	Aromatic	Sorption tube [115]	GC–MS [115]		COVID-19 [115]
4-(1,1-Dimethylpropyl)phenol	Aromatic	Sorption tube [39]	GC–MS [39]	*M. tuberculosis* [39]	Tuberculosis [39]
Ethyl phenol	Aromatic		SIFT-MS [65, 66]		Gastric cancer [65, 66]
Indole	Aromatic/ tryptophan derivative	SPME [66] MCC-IMS [121]	GC–MS [66] MCC-IMS [121]	*E. coli* [40, 41, 121] *P. vulgaris* [65] *P. rettgeri* [65] *P. mirabilis* [65] *K. oxytoca* [65] *P. stuartii* [65]	VAP [121] Wounds [66]
Methyl phenol	Aromatic		SIFT-MS [65, 66]		Gastric cancer [65, 66]
2-Methyl naphthalene	Aromatic	Sorption tube [99]	GC–MS [99]		CF infection
Naphthalene, 1-methyl-	Aromatic	Sorption tube [120]	GC–MS [120]		Tuberculosis [120]
Phenol	Aromatic	SPME [65, 66]	GC–MS SIFT-MS [65, 66]	*P. vulgaris* [65] *P. rettgeri* [65] *P. mirabilis* [65] *K. oxytoca* [65] *P. stuartii* [65]	Wounds [65, 66] Gastric cancer [100, 112, 123]
2,3,6-Trimethylnapthalene	Aromatic	Sorption tube [39]	GC–MS [39]	*M. tuberculosis* [39]	Tuberculosis [39]
Acetic acid	Fatty acid/ fermentation	Sorption tube [37, 123]	GC–MS [37],[40]	*S. aureus* [37, 40, 41]*,* *E. coli* [40, 41] *S. pneumoniae* [122] *H. influenzae* [122]	VAP [37] Gastric cancer [123]
Butyric acid	Fatty acid/ fermentation	Sorption tube [37, 123]	GC–MS [37, 123] PTR-MS [123]	*S. aureus* [37, 38] *C. albicans* [37]	VAP [37] Gastric cancer [123]
Hexanoic acid	Fatty acid	Sorption tube [123]	GC–MS [123] PTR-MS [123] SIFT-MS [100, 112]		Gastric cancer [100, 112, 123]
Pentanoic acid	Fatty acid	Sorption tube [123]	GC–MS [123] PTR-MS [123] SIFT-MS [100, 112]		Gastric cancer [100, 112, 123]
1-Chloroheptane	Halogenated compound		PTR-MS [114]		COVID-19 [114]
1,3-Butadiene	Hydrocarbon	Sorption tube [37]	GC–MS [37]	*S. aureus* [37] *S. pneumoniae* [122]	VAP [37]
Cyclohexene	Hydrocarbon	Sorption tube [115]	GC–MS [115]		COVID-19 [115]
Decane	Hydrocarbon	Sorption tube [116]	GC–MS	*P. aeruginosa* [40, 41] *S. epidermidis* [41] *E. coli* [41]	COVID-19 [109]
4-Ethyl-2,2,6,6-tetramethylheptane	Hydrocarbon	Sorption tube [39, 117]	GC–MS [39]	*M. tuberculosis* [39]	Tuberculosis [39]
Heptane	Hydrocarbon	Sorption tube [86]	GC–MS [86]		VAP [86]
3-Heptene	Hydrocarbon	Sorption tube [108]	GC–MS [108]		COVID-19 [108]
4-Methyl-1-decene	Hydrocarbon	Sorption tube [39]	GC–MS [39]	*M. tuberculosis* [39]	Tuberculosis [39]
2-Methylpropene	Hydrocarbon	Sorption tube [37]	GC–MS [37]	*S. aureus* [37]	VAP [37]
5-Methyl-5-propyl-nonane	Hydrocarbon	SPME [36] Sorption tube [36]	GC–MS [36]	*A. baumannii* [36]	VAP [36]
Nonane	Hydrocarbon	Sorption tube [117]	GC–MS [117]		VAP [117]
Octane	Hydrocarbon	Sorption tube [117]	GC–MS [117]		VAP [117]
2,4-Octadiene	Hydrocarbon		PTR-MS [80]		COVID-19 [80]
1-Octene	Hydrocarbon	Sorption tube [113, 117]	GC–MS [113]		Tuberculosis [113]
Pentadecane	Hydrocarbon	Sorption tube [108, 117]	GC–MS [108, 117]	*P. aeruginosa* [41]	COVID-19 [108] VAP [117]
1,4-Pentadiene	Hydrocarbon	Sorption tube [93]	GC–MS [93]	*S. aureus*	CF infection [93]
Tetradecan**e**	Hydrocarbon	SPME [41] Sorption tube [36, 37, 117]	GC–MS [41]	*A. baumannii* [36] *E. coli* [41]	VAP [36, 37, 117]
Tridecane	Hydrocarbon	Sorption tube [108, 113, 117]	GC–MS [108, 113, 117]	*P. aeruginosa* [40]	COVID-19 [108] Tuberculosis [113] VAP [117]
2,6,10-Trimethyl-dodecane	Hydrocarbon	SPME [36] Sorption tube [36]	GC–MS [36]	*A. baumannii* [36]	VAP [36]
Undecane	Hydrocarbon	Sorption tube [99, 124]	GC–MS [99, 124]		CF infection [99] VAP [124]
1-Undecene	Hydrocarbon	SPME [36] Sorption tube [36]	GC–MS [36]	*A. baumannii* [36] *P. aeruginosa* [36, 41]	VAP [36]
3-Methylbutyric acid	Leucine derivative	SPME [40, 41] Sorption tube [114]	GC–MS [114]	*S. aureus* [38, 40, 41, 114]	VAP [114]
Carane	Monoterpene	Sorption tube [86]	GC–MS [86]		VAP [86]
Longifolene	Sesquiterpene	SPME [36] Sorption tube	GC–MS [36]	*A. baumannii* [36]	VAP [36]

MCC-IMS, multi-capillary column-ion mobility spectrometry; CF, cystic fibrosis; VAP, ventilator-associated pneumonia; H. influenzae, Haemophilus influenzae; P. vulgaris, Proteus vulgaris; P. rettgeri, Proteus rettgeri; P. mirabilis, Proteus mirabilis; P. stuartii, Proteus stuartii; K. oxytoca, Klebsiella oxytoca

## Challenges and emerging directions

Key challenges in clinical volatilomics are associated with upscaling untargeted workflows and developing validated disease-specific targeted assays.

### The “healthy” human volatilome and exogenous volatiles

Eliminating background interference is a complex issue in clinical volatilomics. VOCs are generated from a huge variety of both endogenous and exogenous sources. The various matrices comprising the (healthy) human volatilome collectively comprises 2746 compounds [118] with this number expected to increase in the future [112]. However, it is difficult to validate if this is a true reflection of the human volatilome or if the same compounds are being detected but are being identified inaccurately due to differences in instrumentation or identification criteria. To reliably identify disease-specific volatile biomarkers, the temporal and spatial variations across the healthy human volatilome must be determined. Firstly, understanding the factors influencing the volatilome of healthy individuals day-to-day is critical. All analytical matrices have a background volatilome [119] that must be established. Secondly, setting sufficient controls for interferences introduced from the experimental set-up minimises confounders in the analysis. Background signals will depend on sample collection procedure and must be considered. Factors that influence background during sample collection include direct contact with the sample site; volatilome of the sample collection tool (e.g. cotton swab, PDMS patch, Tedlar bag); relative pre-treatments of sample prior to analysis; and the volatile composition of the (indoor) environment where the sample is taken [120, 121]. Particular caution must be applied to prevent reporting compounds present in indoor air as endogenous.

### Structural identification and validation of volatile biomarkers

As the number of volatile metabolites reported in untargeted studies increases, it is difficult to determine the accuracy of the compound identifications. Wide variation in instrumentation and the compound identification criteria used may result in inaccurate assignments of compounds. This is also prevalent in broader metabolomic research as there are large discrepancies between the number of unique MS features and the effective number of metabolites in biological matrices [122]. For example, in clinical volatilomics, studies (see Table 2) report various branched alkanes as discriminating compounds between disease-associated and non-disease associated volatilomes. Branched alkanes share highly similar mass spectra making accurate manual interpretation and identification difficult. The degree of difficulty in accurately interpreting and identifying these compounds also increases as molecular weight increases due to the higher number of possible structural combinations. This challenge is compounded as branched hydrocarbons with the same number of carbons share similar Kovats retention index values. Due to the low cost, robustness, and sensitivity, single quadrupole mass analysers are the most common mass analysers used for GC–MS analysis. These mass analysers are limited in untargeted screening capabilities by their low resolution and mass accuracy. This means ions of similar masses are poorly differentiated from each other, and that atomic masses and elemental compositions of ions are poorly defined. High-resolution mass spectrometers such as time of flight (TOFs) and orbitraps provide significant improvements in the accurate identification of initially unknown chromatographic peaks as they can potentially determine the number and nature of ionic species present [125]. Universal qualitative identification criteria cover parameters such as signal-to-noise ratios, minimum diagnostic ions, database match scores, retention index windows, and reference standard confirmation for suspected disease-associated biomarkers. Validating untargeted compound screening methods is a challenge as it must ensure robustness and reliability of compound identifications down to specific concentrations. Therefore, if reference materials are available for analytes, screening the LOD is essential to establish the lowest level for which analytes can be reliably and reproducibly identified (95% sensitivity/true positive rate) [126]. Large patient cohort studies are ideally required to validate a threshold concentration of the target VOC that discriminates diseased from non-diseased patients. Understanding matrix effects by comparing chromatographic recovery of target VOCs between spiked matrix samples and pure volatilised analytes is also needed for identifying potential co-eluting interferences and validating future volatilomic assays.

### In vitro volatilomics

**Fig. 4:**
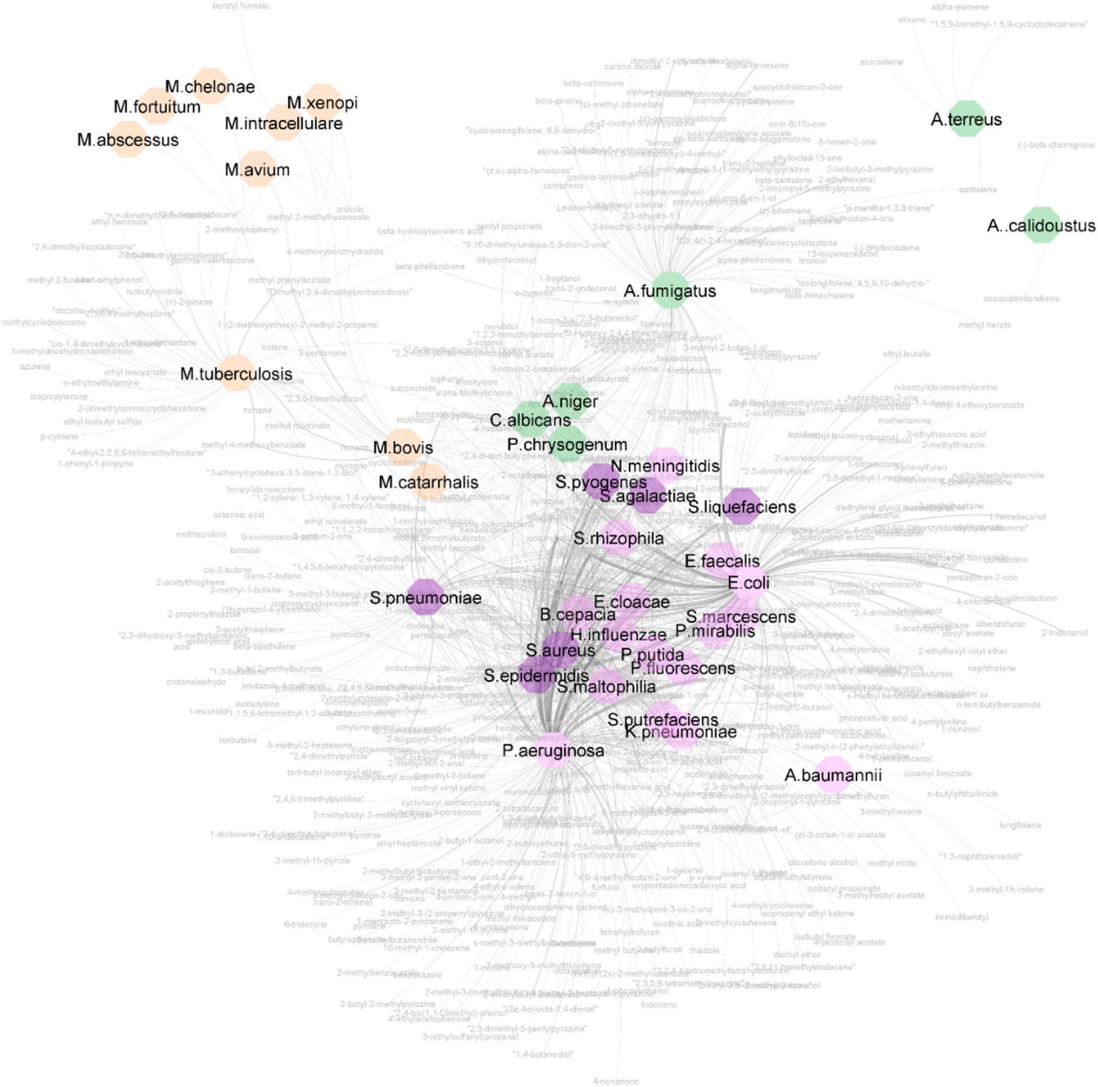
Network graph of microbes based on shared mVOCs. Source nodes are microbes, and target nodes are mVOCs (grey). Microbe colours represent Gram-positive bacteria (purple), Gram-negative bacteria (pink), mycobacteria (orange), and fungi (green). Edges connect microbes to reported mVOCs, where the thickness of the line correlates to the number of times an mVOC is reported for the same microbe (available in high resolution in Electronic Supplementary Materials Fig. S1)

In human hosts, colonised microbes catabolise extracellular sugars, lipids, proteins, amino acids, and metabolites to generate energy for essential cellular processes. In vitro studies demonstrate the chemical diversity of volatile metabolites. The network graph in Fig. 4 (high-resolution version and list of VOCs provided as SI files) compiles microbial culture VOCs reported in literature and illustrates this diversity, where metabolites are shared between microbes with similar characteristics; for example, fungi or mycobacteria share similar volatilomes, as do Gram-negative bacteria. However, the nutrients available to microbes during growth in vivo vary widely in comparison to growth in vitro. Just as in vitro volatile signatures vary across different strains and media composition, resulting infection-associated volatile signatures will be influenced by the site of colonisation due to the site-specific factors such as substrate availability, moisture, pH, oxygen, and temperature.

Human cell lines have been previously used to investigate the volatilomics of cancers [124, 127], inflammation [128], and viruses in vitro [84, 129]. Future in vitro pathogen volatilomics studies should incorporate nutrient-limited media as well as constituents such as keratinocytes, mucus, cholesterol, human cell lines, or blood-based media to achieve a clearer understanding of potential volatile outputs from infected human hosts*.* Organ-on-a-chip technology is currently being investigated as an alternative to animal testing for various applications including infection models [99, 130]. Similar organotypic models have recently been adapted to study volatilomic interactions that occur between pathogens during pulmonary infection [131]. If these models can successfully mimic the pathogenesis of specific microbes in the body, they may provide a route to investigate disease-specific metabolomic and volatilomic trends in the future.

## Conclusion

In the last 15 years, the clinical potential of volatilomics has been demonstrated in numerous studies through the detection of discriminative volatilomic patterns for a variety of infectious diseases. During infection, pathogens metabolise host substrates to generate a diverse set of compounds that contribute to the volatilome of specific diseases. Many studies have utilised untargeted screening of volatiles to discriminate disease-associated and control groups. These disease-associated volatilomic patterns have consisted of abnormal abundances of various normally occurring volatile components of the human volatilome — potentially correlating to inflammation. However, common microbial metabolites have also been consistently detected across various disease-associated groups, and several studies have clearly demonstrated translation of in vitro microbial volatilomics through clinical samples. Screening the volatilomes of potential causative pathogens under varying conditions using the same instrumental workflow being used in the clinical investigation is a clear and simple technique of identifying potential microbial cellular origins of infection-associated compounds. However, microbial volatilomics cannot just simply be translated into targeted clinical volatilomics; standardising instrumental workflows, compound identification, and data processing are critical to ensure that results from the bottom-up are accurate and precise. A collaborative move to address these challenges would significantly promote cross-validation of research and underpin support for large-scale clinical studies to investigate volatilomics of diverse human infections.

### Supplementary Information

Below is the link to the electronic supplementary material.

Fig. S1 (PNG 728 kb)

High resolution image (TIF 116 mb)

Supplementary file2 (PDF 450 KB)
