# Supplementary figures and images for: Volatilomes of human infection
Source: Anal Bioanal Chem. 2023 Oct 16;416(1):37–53. doi: 10.1007/s00216-023-04986-z (PMC10758372; doi:10.1007/s00216-023-04986-z)

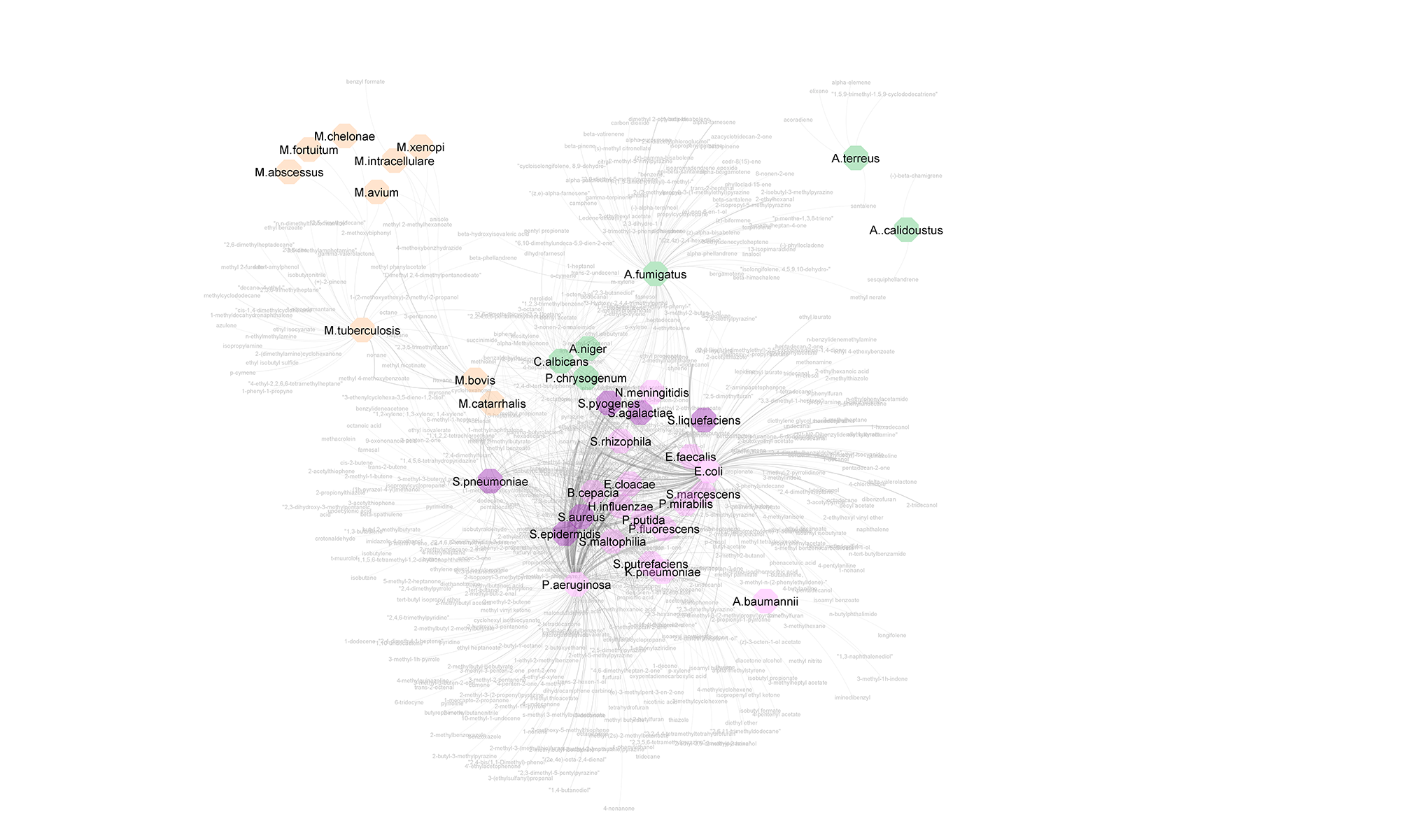

Supplement: Supplementary file 1 — (PNG 728 kb) [file 216_2023_4986_Fig5_ESM.png]
