# Supplementary material for: Volatilomes of human infection
Source: Anal Bioanal Chem. 2023 Oct 16;416(1):37–53. doi: 10.1007/s00216-023-04986-z (PMC10758372; doi:10.1007/s00216-023-04986-z)
Supplement: Supplementary file 3 — Supplementary file2 (PDF 450 KB) [file 216_2023_4986_MOESM2_ESM.pdf]

Supplementary Information
Volatilomes of Human Infection

Shane Fitzgerald,[a] Linda Holland,[b] Waqar Ahmed,[c] Birgit Piechulla,[d] Stephen J Fowler,[c,e] Aoife Morrin[a]

[a]SFI Insight Centre For Data Analytics; National Centre for Sensor Research; School of Chemical Sciences, Dublin City University, Ireland
[b]School of Biotechnology, Dublin City University, Ireland
[c]Division of Infection, Immunity and Respiratory Medicine, School of Biological Sciences, The University of Manchester, UK
[d]Institute of Biological Sciences, University of Rostock, Germany
[e]Respiratory Medicine, Manchester Academic Health Science Centre, Manchester University NHS Foundation Trust, UK

Dataset of literature-reported mVOCs according to genus and species including reference DOI

Dataset used to generate network plot (Figure 4)

Compound Name	Genus	Species	Publication DOI
**(-)-alpha-terpineol**	Aspergillus	fumigatus	10.1128/EC.00074-14
**(-)-beta-chamigrene**	Aspergillus	calidoustus	10.1093/cid/ciu725
**(-)-beta-pinene**	Aspergillus	fumigatus	10.1128/EC.00074-14
**(-)-phyllocladene**	Aspergillus	fumigatus	10.1128/EC.00074-14
**(-)-threo-isodihomocitric acid**	Stenotrophomonas	maltophilia	10.3390/metabo11110773
**(+)-2-pinene**	Mycobacterium	tuberculosis	10.1088/1752-7163/aacd18
**(1h-pyrazol-4-yl)methanol**	Streptococcus	pneumoniae	10.3390/metabo11110773
**(1-methylpentyl)cyclopropane**	Staphylococcus	aureus	10.3390/metabo10090347
**(1-methylpentyl)cyclopropane**	Staphylococcus	epidermidis	10.3390/metabo10090347
**(2e,4e)-octa-2,4-dienal**	Pseudomonas	aeruginosa	10.1128/mSphere.00843-20
**(2z,4z)-2,4-hexadiene**	Aspergillus	fumigatus	10.1128/EC.00074-14
**(e)-3-methylpent-3-en-2-one**	Pseudomonas	aeruginosa	10.1016/j.jchromb.2012.05.038
**(e)-non-6-en-1-ol**	Aspergillus	fumigatus	10.1128/EC.00074-14
**(r)-beta-bisabolene**	Aspergillus	fumigatus	10.1128/EC.00074-14
**(s)-methyl citronellate**	Aspergillus	fumigatus	10.1128/EC.00074-14
**(z)-3-octen-1-ol acetate**	Klebsiella	pneumoniae	10.1088/1752-7155/10/2/027101
**(z)-alpha-bisabolene**	Aspergillus	fumigatus	10.1128/EC.00074-14
**(z)-biformene**	Aspergillus	fumigatus	10.1128/EC.00074-14
**(z)-gamma-bisabolene**	Aspergillus	fumigatus	10.1088/1752-7155/6/1/016002
**(z)-gamma-bisabolene**	Aspergillus	fumigatus	10.1088/1752-7155/6/1/016002
**(z)-gamma-bisabolene**	Aspergillus	fumigatus	10.1088/1752-7155/6/1/016002
**(z,e)-alpha-farnesene**	Aspergillus	fumigatus	10.1128/EC.00074-14
**1-(2-methoxyethoxy)-2-methyl-2-propanol**	Mycobacterium	bovis	10.1088/1752-7163/aa6e06
**1,1,2,2-tetrachloroethane**	Staphylococcus	aureus	10.1088/1752-7155/8/2/027106
**1,1,5,6-tetramethyl-1,2-dihydronaphthalene**	Staphylococcus	epidermidis	10.3390/metabo10090347
**1,10-undecadiene**	Pseudomonas	aeruginosa	10.1186/1471-2180-12-113
**1,2,3-trimethylbenzene**	Candida	albicans	10.1038/srep27441
**1,2,3-trimethylbenzene**	Penicillium	chrysogenum	10.1038/srep27441
**1,2,3-trimethylbenzene**	Aspergillus	niger	10.1038/srep27441
**1,2-xylene; 1,3-xylene; 1,4-xylene**	Staphylococcus	epidermidis	10.3390/metabo10090347
**1,2-xylene; 1,3-xylene; 1,4-xylene**	Staphylococcus	epidermidis	10.3390/metabo10090347

**1,2-xylene; 1,3-xylene; 1,4-xylene**	Mycobacterium	tuberculosis	10.1088/1752-7163/aacd18
**1,3-butadiene**	Streptococcus	pneumoniae	10.1099/mic.0.062687-0
**1,3-di-tert-butylbenzene**	Staphylococcus	aureus	10.3390/metabo10090347
**1,3-di-tert-butylbenzene**	Staphylococcus	epidermidis	10.3390/metabo10090347
**1,3-naphthalenediol**	Acinetobacter	baumannii	10.1088/1752-7155/10/2/027102
**1,4,5,6-tetrahydropyridazine**	Staphylococcus	aureus	10.1109/JSEN.2009.2035671
**1,4,5,6-tetrahydropyridazine**	Staphylococcus	aureus	10.1109/JSEN.2009.2035671
**1,4-butanediol**	Staphylococcus	aureus	10.3389/fmicb.2021.693075
**1,4-butanediol**	Staphylococcus	aureus	10.3389/fmicb.2021.693075
**1,4-dichlorobenzene**	Staphylococcus	aureus	10.1088/1752-7155/8/2/027106
**1,5,9-trimethyl-1,5,9-cyclododecatriene**	Aspergillus	terreus	10.1093/cid/ciu725
**1,6-heptadiene, 2-methyl-6-phenyl-**	Candida	albicans	10.1038/srep27441
**1,6-heptadiene, 2-methyl-6-phenyl-**	Penicillium	chrysogenum	10.1038/srep27441
**1,6-heptadiene, 2-methyl-6-phenyl-**	Aspergillus	niger	10.1038/srep27441
**10-methyl-1-undecene**	Pseudomonas	aeruginosa	10.1186/1471-2180-12-113
**13-isopimaradiene**	Aspergillus	fumigatus	10.1128/EC.00074-14
**1-butanamine, 3-methyl-n-(2-phenylethylidene)-**	Proteus	mirabilis	10.1007/s00253-012-3924-4
**1-butanamine, 3-methyl-n-(2-phenylethylidene)-**	Proteus	mirabilis	10.1007/s00253-012-3924-4
**1-butanol**	Pseudomonas	aeruginosa	10.1016/j.jchromb.2012.05.038
**1-butanol**	Pseudomonas	aeruginosa	10.1016/j.mimet.2010.12.001
**1-butanol**	Pseudomonas	aeruginosa	10.1016/j.mimet.2010.12.001
**1-butanol**	Pseudomonas	aeruginosa	10.1016/S0378-4347(00)80760-4
**1-butanol**	Pseudomonas	aeruginosa	10.1016/S0378-4347(00)80760-4
**1-butanol**	Pseudomonas	aeruginosa	10.1128/JCM.12.4.521-526.1980
**1-butanol**	Pseudomonas	aeruginosa	10.1128/JCM.12.4.521-526.1980
**1-butanol**	Pseudomonas	aeruginosa	10.1128/JCM.12.4.521-526.1980
**1-butanol**	Pseudomonas	aeruginosa	10.1128/JCM.12.4.521-526.1980
**1-butanol**	Pseudomonas	aeruginosa	10.1128/JCM.12.4.521-526.1980
**1-butanol**	Pseudomonas	aeruginosa	10.1128/JCM.12.4.521-526.1980
**1-butanol**	Pseudomonas	aeruginosa	10.1128/JCM.12.4.521-526.1980
**1-butanol**	Pseudomonas	aeruginosa	10.1128/JCM.12.4.521-526.1980
**1-butanol**	Pseudomonas	aeruginosa	10.1128/JCM.12.4.521-526.1980
**1-butanol**	Pseudomonas	aeruginosa	10.1128/JCM.12.4.521-526.1980
**1-butanol**	Pseudomonas	aeruginosa	10.1128/JCM.12.4.521-526.1980
**1-butanol**	Pseudomonas	aeruginosa	10.3389/fmicb.2021.693075
**1-butanol**	Pseudomonas	aeruginosa	10.3389/fmicb.2021.693075
**1-butanol**	Pseudomonas	aeruginosa	10.3389/fmicb.2021.693075
**1-butanol**	Pseudomonas	aeruginosa	10.3389/fmicb.2021.693075
**1-butanol**	Pseudomonas	aeruginosa	10.3389/fmicb.2021.693075
**1-butanol**	Candida	albicans	10.1038/srep27441
**1-butanol**	Staphylococcus	aureus	10.1016/j.mimet.2010.12.001
**1-butanol**	Staphylococcus	aureus	10.1016/j.mimet.2010.12.001
**1-butanol**	Staphylococcus	aureus	10.1109/JSEN.2009.2035671

**1-butanol**	Staphylococcus	aureus	10.1128/JCM.00392-10
**1-butanol**	Staphylococcus	aureus	10.1186/1471-2180-12-113
**1-butanol**	Burkholderia	cepacia	10.1016/j.mimet.2010.12.001
**1-butanol**	Penicillium	chrysogenum	10.1038/srep27441
**1-butanol**	Escherichia	coli	10.1016/j.mimet.2010.12.001
**1-butanol**	Escherichia	coli	10.1016/j.mimet.2010.12.001
**1-butanol**	Escherichia	coli	10.1038/s41598-020-74909-w
**1-butanol**	Escherichia	coli	10.1038/s41598-020-74909-w
**1-butanol**	Escherichia	coli	10.1128/AEM.02069-07
**1-butanol**	Escherichia	coli	10.3389/fmicb.2021.693075
**1-butanol**	Escherichia	coli	10.3389/fmicb.2021.693075
**1-butanol**	Escherichia	coli	10.3389/fmicb.2021.693075
**1-butanol**	Escherichia	coli	10.3389/fmicb.2021.693075
**1-butanol**	Escherichia	coli	10.3389/fmicb.2021.693075
**1-butanol**	Escherichia	coli	10.3389/fmicb.2021.693075
**1-butanol**	Escherichia	coli	10.3390/antibiotics9110797
**1-butanol**	Escherichia	coli	10.3390/antibiotics9110797
**1-butanol**	Staphylococcus	epidermidis	10.1038/s41598-020-74909-w
**1-butanol**	Enterococcus	faecalis	10.1016/j.mimet.2010.12.001
**1-butanol**	Pseudomonas	fluorescens	10.1128/JCM.12.4.521-526.1980
**1-butanol**	Haemophilus	influenzae	10.1099/mic.0.062687-0
**1-butanol**	Stenotrophomonas	maltophilia	10.1128/JCM.12.4.521-526.1980
**1-butanol**	Proteus	mirabilis	10.1016/j.mimet.2010.12.001
**1-butanol**	Proteus	mirabilis	10.1016/j.mimet.2010.12.001
**1-butanol**	Aspergillus	niger	10.1038/srep27441
**1-butanol**	Klebsiella	pneumoniae	10.1111/jam.13372
**1-butanol**	Streptococcus	pneumoniae	10.1099/mic.0.062687-0
**1-butanol**	Pseudomonas	putida	10.1128/JCM.12.4.521-526.1980
**1-butanol**	Shewanella	putrefaciens	10.1128/JCM.12.4.521-526.1980
**1-butanol**	Streptococcus	pyogenes	10.1016/j.mimet.2010.12.001
**1-butoxy-2-propanol**	Pseudomonas	aeruginosa	10.1128/mSphere.00843-20
**1-decanol**	Escherichia	coli	10.1002/jobm.201600505
**1-decanol**	Escherichia	coli	10.1007/s00253-013-4762-8
**1-decanol**	Escherichia	coli	10.1007/s00253-013-4762-8
**1-decanol**	Escherichia	coli	10.1007/s00284-005-4469-x
**1-decanol**	Escherichia	coli	10.1007/s00284-005-4469-x
**1-decanol**	Escherichia	coli	10.1007/s00284-005-4469-x
**1-decanol**	Escherichia	coli	10.1007/s00284-005-4469-x
**1-decanol**	Escherichia	coli	10.1007/s00284-005-4469-x
**1-decanol**	Escherichia	coli	10.1007/s00284-005-4469-x
**1-decanol**	Escherichia	coli	10.1007/s00284-005-4469-x
**1-decanol**	Escherichia	coli	10.1007/s00284-005-4469-x
**1-decanol**	Escherichia	coli	10.1007/s00284-005-4469-x

**1-decanol**	Escherichia	coli	10.1007/s00284-005-4469-x
**1-decanol**	Escherichia	coli	10.1038/s41598-020-74909-w
**1-decanol**	Escherichia	coli	10.1093/chromsci/bmt042
**1-decanol**	Escherichia	coli	10.1093/chromsci/bmt042
**1-decanol**	Escherichia	coli	10.1093/chromsci/bmt042
**1-decanol**	Escherichia	coli	10.3389/fmicb.2021.693075
**1-decanol**	Escherichia	coli	10.3389/fmicb.2021.693075
**1-decanol**	Escherichia	coli	10.3389/fmicb.2021.693075
**1-decanol**	Escherichia	coli	10.3389/fmicb.2021.693075
**1-decanol**	Escherichia	coli	10.3389/fmicb.2021.693075
**1-decanol**	Escherichia	coli	10.3389/fmicb.2021.693075
**1-decene**	Pseudomonas	aeruginosa	10.1186/1471-2180-12-113
**1-dodecanol**	Candida	albicans	10.1128/EC.00252-07
**1-dodecanol**	Escherichia	coli	10.1002/jobm.201600505
**1-dodecanol**	Escherichia	coli	10.1007/s00284-005-4469-x
**1-dodecanol**	Escherichia	coli	10.1007/s00284-005-4469-x
**1-dodecanol**	Escherichia	coli	10.1007/s00284-005-4469-x
**1-dodecanol**	Escherichia	coli	10.1007/s00284-005-4469-x
**1-dodecanol**	Escherichia	coli	10.1007/s00284-005-4469-x
**1-dodecanol**	Escherichia	coli	10.1007/s00284-005-4469-x
**1-dodecanol**	Escherichia	coli	10.1007/s00284-005-4469-x
**1-dodecanol**	Escherichia	coli	10.1007/s00284-005-4469-x
**1-dodecanol**	Escherichia	coli	10.1007/s00284-005-4469-x
**1-dodecanol**	Escherichia	coli	10.1007/s00284-005-4469-x
**1-dodecanol**	Escherichia	coli	10.1038/s41598-020-74909-w
**1-dodecanol**	Escherichia	coli	10.1038/s41598-020-74909-w
**1-dodecanol**	Escherichia	coli	10.1093/chromsci/bmt042
**1-dodecanol**	Escherichia	coli	10.1093/chromsci/bmt042
**1-dodecanol**	Escherichia	coli	10.1093/chromsci/bmt042
**1-dodecanol**	Escherichia	coli	10.3389/fmicb.2021.693075
**1-dodecanol**	Escherichia	coli	10.3389/fmicb.2021.693075
**1-dodecanol**	Escherichia	coli	10.3389/fmicb.2021.693075
**1-dodecanol**	Escherichia	coli	10.3389/fmicb.2021.693075
**1-dodecanol**	Escherichia	coli	10.3389/fmicb.2021.693075
**1-dodecanol**	Escherichia	coli	10.3389/fmicb.2021.693075
**1-dodecene**	Pseudomonas	aeruginosa	10.1186/1471-2180-12-113
**1-ethenylaziridine**	Pseudomonas	aeruginosa	10.1186/1471-2180-12-113
**1-ethenylaziridine**	Klebsiella	pneumoniae	10.1088/1752-7155/10/2/027101
**1-ethyl-2-methylbenzene**	Staphylococcus	aureus	10.3390/metabo10090347
**1-heptanol**	Candida	albicans	10.1038/srep27441
**1-heptanol**	Aspergillus	niger	10.1038/srep27441
**1-hexadecanol**	Escherichia	coli	10.1038/s41598-020-74909-w
**1-hexadecanol**	Escherichia	coli	10.3389/fmicb.2021.693075

**1-hexadecanol**	Escherichia	coli	10.3389/fmicb.2021.693075
**1-hexadecanol**	Escherichia	coli	10.3389/fmicb.2021.693075
**1-hexadecanol**	Escherichia	coli	10.3389/fmicb.2021.693075
**1-hexadecanol**	Escherichia	coli	10.3389/fmicb.2021.693075
**1-hexadecanol**	Escherichia	coli	10.3389/fmicb.2021.693075
**1-hexanol**	Candida	albicans	10.1038/srep27441
**1-hexanol**	Penicillium	chrysogenum	10.1038/srep27441
**1-hexanol**	Escherichia	coli	10.1038/s41598-020-74909-w
**1-hexanol**	Escherichia	coli	10.1038/s41598-020-74909-w
**1-hexanol**	Escherichia	coli	10.1088/1752-7163/aa8efc
**1-hexanol**	Escherichia	coli	10.1111/jam.15716
**1-hexanol**	Escherichia	coli	10.1111/jam.15716
**1-hexanol**	Aspergillus	niger	10.1038/srep27441
**1-mercapto-2-propanone**	Pseudomonas	aeruginosa	10.1186/1471-2180-12-113
**1-methoxy-2-propyl acetate**	Escherichia	coli	10.3390/antibiotics9110797
**1-methyl-2-pyrrolidinone**	Escherichia	coli	10.1111/jam.15716
**1-methyl-2-pyrrolidinone**	Escherichia	coli	10.1111/jam.15716
**1-methyldecahydronaphthalene**	Mycobacterium	tuberculosis	10.1016/j.tube.2006.03.004
**1-methyldecahydronaphthalene**	Mycobacterium	tuberculosis	10.1016/j.tube.2006.03.004
**1-methylnaphthalene**	Enterobacter	cloacae	10.1007/s00253-012-3924-4
**1-methylnaphthalene**	Escherichia	coli	10.1007/s00253-012-3924-4
**1-methylnaphthalene**	Mycobacterium	tuberculosis	10.1016/j.tube.2006.03.004
**1-methylnaphthalene**	Mycobacterium	tuberculosis	10.1016/j.tube.2006.03.004
**1-nitroadamantane**	Mycobacterium	tuberculosis	10.1088/1752-7163/aacd18
**1-nonanol**	Escherichia	coli	10.1111/jam.15716
**1-nonanol**	Escherichia	coli	10.1111/jam.15716
**1-nonene**	Pseudomonas	aeruginosa	10.1186/1471-2180-12-113
**1-octanol**	Pseudomonas	aeruginosa	10.1186/s13568-022-01367-0
**1-octanol**	Candida	albicans	10.1038/srep27441
**1-octanol**	Staphylococcus	aureus	10.3390/metabo10090347
**1-octanol**	Penicillium	chrysogenum	10.1038/srep27441
**1-octanol**	Escherichia	coli	10.1002/jobm.201600505
**1-octanol**	Escherichia	coli	10.1007/s00253-013-4762-8
**1-octanol**	Escherichia	coli	10.1007/s00253-013-4762-8
**1-octanol**	Escherichia	coli	10.1007/s00284-005-4469-x
**1-octanol**	Escherichia	coli	10.1007/s00284-005-4469-x
**1-octanol**	Escherichia	coli	10.1007/s00284-005-4469-x
**1-octanol**	Escherichia	coli	10.1007/s00284-005-4469-x
**1-octanol**	Escherichia	coli	10.1007/s00284-005-4469-x
**1-octanol**	Escherichia	coli	10.1007/s00284-005-4469-x
**1-octanol**	Escherichia	coli	10.1007/s00284-005-4469-x
**1-octanol**	Escherichia	coli	10.1007/s00284-005-4469-x
**1-octanol**	Escherichia	coli	10.1007/s00284-005-4469-x

**1-octanol**	Escherichia	coli	10.1007/s00284-005-4469-x
**1-octanol**	Escherichia	coli	10.1038/s41598-020-74909-w
**1-octanol**	Escherichia	coli	10.1038/s41598-020-74909-w
**1-octanol**	Escherichia	coli	10.1093/chromsci/bmt042
**1-octanol**	Escherichia	coli	10.1093/chromsci/bmt042
**1-octanol**	Escherichia	coli	10.3389/fmicb.2021.693075
**1-octanol**	Escherichia	coli	10.3389/fmicb.2021.693075
**1-octanol**	Escherichia	coli	10.3389/fmicb.2021.693075
**1-octanol**	Escherichia	coli	10.3389/fmicb.2021.693075
**1-octanol**	Escherichia	coli	10.3389/fmicb.2021.693075
**1-octanol**	Escherichia	coli	10.3389/fmicb.2021.693075
**1-octanol**	Aspergillus	niger	10.1038/srep27441
**1-octen-3-ol**	Candida	albicans	10.1038/srep27441
**1-octen-3-ol**	Penicillium	chrysogenum	10.1038/srep27441
**1-octen-3-ol**	Aspergillus	fumigatus	10.1128/EC.00074-14
**1-octen-3-ol**	Aspergillus	niger	10.1038/srep27441
**1-pentadecanol**	Escherichia	coli	10.3389/fmicb.2021.693075
**1-pentadecanol**	Escherichia	coli	10.3389/fmicb.2021.693075
**1-pentadecanol**	Escherichia	coli	10.3389/fmicb.2021.693075
**1-pentanol**	Pseudomonas	aeruginosa	10.1016/j.mimet.2010.12.001
**1-pentanol**	Staphylococcus	aureus	10.1016/j.mimet.2010.12.001
**1-pentanol**	Staphylococcus	aureus	10.1016/j.mimet.2010.12.001
**1-pentanol**	Burkholderia	cepacia	10.1016/j.mimet.2010.12.001
**1-pentanol**	Escherichia	coli	10.1016/j.mimet.2005.09.003
**1-pentanol**	Escherichia	coli	10.1016/j.mimet.2005.09.016
**1-pentanol**	Escherichia	coli	10.1016/j.mimet.2010.12.001
**1-pentanol**	Escherichia	coli	10.1016/j.mimet.2010.12.001
**1-pentanol**	Escherichia	coli	10.3390/antibiotics9110797
**1-pentanol**	Enterococcus	faecalis	10.1016/j.mimet.2010.12.001
**1-pentanol**	Stenotrophomonas	maltophilia	10.1088/1752-7155/9/2/027104
**1-pentanol**	Proteus	mirabilis	10.1016/j.mimet.2010.12.001
**1-pentanol**	Proteus	mirabilis	10.1016/j.mimet.2010.12.001
**1-pentanol**	Streptococcus	pyogenes	10.1016/j.mimet.2010.12.001
**1-pentanol**	Stenotrophomonas	rhizophila	10.1088/1752-7155/9/2/027104
**1-phenyl-1-propyne**	Mycobacterium	tuberculosis	10.1088/1752-7163/aacd18
**1-phenylethanol**	Pseudomonas	aeruginosa	10.1016/j.jchromb.2012.05.038
**1-propanol**	Pseudomonas	aeruginosa	10.1088/1752-7155/10/3/037102
**1-propanol**	Pseudomonas	aeruginosa	10.1088/1752-7155/10/3/037102
**1-propanol**	Pseudomonas	aeruginosa	10.1088/1752-7155/10/3/037102
**1-propanol**	Staphylococcus	aureus	10.1088/1752-7155/10/3/037102
**1-propanol**	Staphylococcus	aureus	10.1088/1752-7155/10/3/037102
**1-propanol**	Staphylococcus	aureus	10.1088/1752-7155/10/3/037102
**1-propanol**	Staphylococcus	aureus	10.1109/JSEN.2009.2035671

**1-propanol**	Staphylococcus	aureus	10.1109/JSEN.2009.2035671
**1-propanol**	Burkholderia	cepacia	10.1088/1752-7155/10/3/037102
**1-propanol**	Burkholderia	cepacia	10.1088/1752-7155/10/3/037102
**1-propanol**	Burkholderia	cepacia	10.1088/1752-7155/10/3/037102
**1-propanol**	Escherichia	coli	10.1016/j.mimet.2005.09.003
**1-propanol**	Escherichia	coli	10.1088/1752-7155/8/2/027106
**1-propanol**	Escherichia	coli	10.1088/1752-7155/8/2/027106
**1-propanol**	Escherichia	coli	10.3389/fmicb.2021.693075
**1-propanol**	Escherichia	coli	10.3389/fmicb.2021.693075
**1-propanol**	Escherichia	coli	10.3389/fmicb.2021.693075
**1-propanol**	Escherichia	coli	10.3389/fmicb.2021.693075
**1-propanol**	Escherichia	coli	10.3390/antibiotics9110797
**1-propanol**	Escherichia	coli	10.3390/antibiotics9110797
**1-propanol**	Escherichia	coli	10.3390/antibiotics9110797
**1-propanol**	Stenotrophomonas	maltophilia	10.1088/1752-7155/10/3/037102
**1-propanol**	Stenotrophomonas	maltophilia	10.1088/1752-7155/10/3/037102
**1-propanol**	Stenotrophomonas	maltophilia	10.1088/1752-7155/10/3/037102
**1-propanol**	Klebsiella	pneumoniae	10.1088/1752-7155/8/2/027106
**1-tetradecanol**	Escherichia	coli	10.1038/s41598-020-74909-w
**1-tetradecanol**	Escherichia	coli	10.1038/s41598-020-74909-w
**1-tetradecanol**	Escherichia	coli	10.1093/chromsci/bmt042
**1-tetradecanol**	Escherichia	coli	10.1093/chromsci/bmt042
**1-tetradecanol**	Escherichia	coli	10.1093/chromsci/bmt042
**1-tetradecanol**	Escherichia	coli	10.3389/fmicb.2021.693075
**1-tetradecanol**	Escherichia	coli	10.3389/fmicb.2021.693075
**1-tetradecanol**	Escherichia	coli	10.3389/fmicb.2021.693075
**1-tetradecanol**	Escherichia	coli	10.3389/fmicb.2021.693075
**1-tetradecanol**	Escherichia	coli	10.3389/fmicb.2021.693075
**1-tetradecanol**	Escherichia	coli	10.3389/fmicb.2021.693075
**1-tridecanol**	Escherichia	coli	10.3389/fmicb.2021.693075
**1-tridecanol**	Escherichia	coli	10.3389/fmicb.2021.693075
**1-tridecanol**	Escherichia	coli	10.3389/fmicb.2021.693075
**1-undecene**	Pseudomonas	aeruginosa	10.1007/s00253-012-3924-4
**1-undecene**	Pseudomonas	aeruginosa	10.1007/s11306-018-1357-5
**1-undecene**	Pseudomonas	aeruginosa	10.1016/j.jchromb.2009.05.028
**1-undecene**	Pseudomonas	aeruginosa	10.1016/S0045-6535(97)00209-9
**1-undecene**	Pseudomonas	aeruginosa	10.1016/S0378-4347(00)80760-4
**1-undecene**	Pseudomonas	aeruginosa	10.1016/S0378-4347(00)80760-4
**1-undecene**	Pseudomonas	aeruginosa	10.1038/s41598-020-74909-w
**1-undecene**	Pseudomonas	aeruginosa	10.1038/s41598-020-74909-w
**1-undecene**	Pseudomonas	aeruginosa	10.1088/1752-7155/10/1/016002
**1-undecene**	Pseudomonas	aeruginosa	10.1088/1752-7155/8/2/027106
**1-undecene**	Pseudomonas	aeruginosa	10.1088/1752-7155/8/2/027106

**1-undecene**	Pseudomonas	aeruginosa	10.1088/1752-7163/aa8efc
**1-undecene**	Pseudomonas	aeruginosa	10.1088/1752-7163/aa8efc
**1-undecene**	Pseudomonas	aeruginosa	10.1128/JCM.12.4.521-526.1980
**1-undecene**	Pseudomonas	aeruginosa	10.1128/JCM.12.4.521-526.1980
**1-undecene**	Pseudomonas	aeruginosa	10.1128/JCM.12.4.521-526.1980
**1-undecene**	Pseudomonas	aeruginosa	10.1128/JCM.12.4.521-526.1980
**1-undecene**	Pseudomonas	aeruginosa	10.1128/JCM.12.4.521-526.1980
**1-undecene**	Pseudomonas	aeruginosa	10.1128/JCM.12.4.521-526.1980
**1-undecene**	Pseudomonas	aeruginosa	10.1128/JCM.12.4.521-526.1980
**1-undecene**	Pseudomonas	aeruginosa	10.1128/JCM.12.4.521-526.1980
**1-undecene**	Pseudomonas	aeruginosa	10.1128/JCM.12.4.521-526.1980
**1-undecene**	Pseudomonas	aeruginosa	10.1128/JCM.12.4.521-526.1980
**1-undecene**	Pseudomonas	aeruginosa	10.1128/JCM.12.4.521-526.1980
**1-undecene**	Pseudomonas	aeruginosa	10.1186/1471-2180-12-113
**1-undecene**	Pseudomonas	aeruginosa	10.3389/fmicb.2021.693075
**1-undecene**	Pseudomonas	aeruginosa	10.3389/fmicb.2021.693075
**1-undecene**	Pseudomonas	aeruginosa	10.3389/fmicb.2021.693075
**1-undecene**	Pseudomonas	aeruginosa	10.3389/fmicb.2021.693075
**1-undecene**	Pseudomonas	aeruginosa	10.3389/fmicb.2021.693075
**1-undecene**	Pseudomonas	aeruginosa	10.3389/fmicb.2021.693075
**1-undecene**	Acinetobacter	baumannii	10.1088/1752-7155/10/2/027102
**1-undecene**	Pseudomonas	fluorescens	10.1016/S0045-6535(97)00209-9
**1-undecene**	Pseudomonas	fluorescens	10.1128/JCM.12.4.521-526.1980
**1-undecene**	Stenotrophomonas	maltophilia	10.1128/JCM.12.4.521-526.1980
**1-undecene**	Pseudomonas	putida	10.1016/S0045-6535(97)00209-9
**1-undecene**	Pseudomonas	putida	10.1128/JCM.12.4.521-526.1980
**1-undecene**	Shewanella	putrefaciens	10.1128/JCM.12.4.521-526.1980
**2 3-dihydro-1 1 3-trimethyl-3-phenyl-1h-indene**	Aspergillus	fumigatus	10.1088/1752-7155/10/1/016002
**2-(2-methylpropyl)-3-(1-methylethyl)pyrazine**	Aspergillus	fumigatus	10.1128/EC.00074-14
**2(3h)-furanone, 5-dodecyldihydro-**	Escherichia	coli	10.1111/jam.15716
**2(3h)-furanone, 5-dodecyldihydro-**	Escherichia	coli	10.1111/jam.15716
**2-(dimethylamino)cyclohexanone**	Mycobacterium	tuberculosis	10.5588/ijtld.11.0576
**2-(methylthio)ethanol**	Streptococcus	agalactiae	10.1007/s00253-012-3924-4
**2-(methylthio)ethanol**	Staphylococcus	aureus	10.1007/s00253-012-3924-4
**2-(methylthio)ethanol**	Enterobacter	cloacae	10.1007/s00253-012-3924-4
**2-(methylthio)ethanol**	Escherichia	coli	10.1002/jssc.201800684
**2-(methylthio)ethanol**	Escherichia	coli	10.1007/s00253-012-3924-4
**2-(methylthio)ethanol**	Serratia	marcescens	10.1007/s00253-012-3924-4
**2-(methylthio)ethanol**	Proteus	mirabilis	10.1007/s00253-012-3924-4
**2-(methylthio)ethanol**	Klebsiella	pneumoniae	10.1007/s00253-012-3924-4
**2,2,4,4-tetramethyltetrahydrofuran**	Klebsiella	pneumoniae	10.1111/jam.13372
**2,2,4,6,6-pentamethylheptane**	Escherichia	coli	10.3390/antibiotics9110797
**2,2,4,6,6-pentamethylheptane**	Mycobacterium	tuberculosis	10.1016/j.tube.2006.03.004

**2,2,4,6,6-pentamethylheptane**	Mycobacterium	tuberculosis	10.1016/j.tube.2006.03.004
**2,3,5,6-tetramethylpyrazine**	Pseudomonas	aeruginosa	10.1128/mSphere.00843-20
**2,3,5-trimethylfuran**	Mycobacterium	bovis	10.1371/journal.pone.0194348
**2,3,5-trimethylpyrazine**	Pseudomonas	aeruginosa	10.1038/s41598-020-74909-w
**2,3,5-trimethylpyrazine**	Pseudomonas	aeruginosa	10.1038/s41598-020-74909-w
**2,3,5-trimethylpyrazine**	Staphylococcus	aureus	10.1038/s41598-020-74909-w
**2,3,5-trimethylpyrazine**	Staphylococcus	aureus	10.1038/s41598-020-74909-w
**2,3,5-trimethylpyrazine**	Staphylococcus	aureus	10.3390/metabo10090347
**2,3,5-trimethylpyrazine**	Staphylococcus	aureus	10.3390/metabo10090347
**2,3,5-trimethylpyrazine**	Staphylococcus	aureus	10.3390/metabo10090347
**2,3,5-trimethylpyrazine**	Enterobacter	cloacae	10.1007/s00253-012-3924-4
**2,3,5-trimethylpyrazine**	Escherichia	coli	10.1007/s00253-012-3924-4
**2,3,5-trimethylpyrazine**	Escherichia	coli	10.1038/s41598-020-74909-w
**2,3,5-trimethylpyrazine**	Escherichia	coli	10.1038/s41598-020-74909-w
**2,3,5-trimethylpyrazine**	Staphylococcus	epidermidis	10.1038/s41598-020-74909-w
**2,3,5-trimethylpyrazine**	Staphylococcus	epidermidis	10.1038/s41598-020-74909-w
**2,3,5-trimethylpyrazine**	Staphylococcus	epidermidis	10.3390/metabo10090347
**2,3,5-trimethylpyrazine**	Staphylococcus	epidermidis	10.3390/metabo10090347
**2,3,5-trimethylpyrazine**	Staphylococcus	epidermidis	10.3390/metabo10090347
**2,3,5-trimethylpyrazine**	Serratia	marcescens	10.1007/s00253-012-3924-4
**2,3,6-trimethylheptane**	Mycobacterium	tuberculosis	10.1088/1752-7163/aacd18
**2,3-butanediol**	Candida	albicans	10.3390/metabo12050432
**2,3-butanedione**	Pseudomonas	aeruginosa	10.1016/j.jchromb.2012.05.038
**2,3-butanedione**	Staphylococcus	aureus	10.1186/1471-2180-12-113
**2,3-butanedione**	Staphylococcus	aureus	10.3390/metabo10090347
**2,3-butanedione**	Staphylococcus	aureus	10.3390/metabo10090347
**2,3-butanedione**	Staphylococcus	epidermidis	10.3390/metabo10090347
**2,3-butanedione**	Haemophilus	influenzae	10.1099/mic.0.062687-0
**2,3-butanedione**	Klebsiella	pneumoniae	10.1088/1752-7155/10/2/027101
**2,3-butanedione**	Klebsiella	pneumoniae	10.1111/jam.13372
**2,3-butanedione**	Streptococcus	pneumoniae	10.1016/j.jchromb.2018.08.032
**2,3-butanedione**	Streptococcus	pneumoniae	10.1016/j.jchromb.2018.08.032
**2,3-butanedione**	Streptococcus	pneumoniae	10.1016/j.jchromb.2018.08.032
**2,3-butanedione**	Streptococcus	pneumoniae	10.1016/j.jchromb.2018.08.032
**2,3-butanedione**	Streptococcus	pneumoniae	10.1016/j.jchromb.2018.08.032
**2,3-butanedione**	Streptococcus	pneumoniae	10.1016/j.jchromb.2018.08.032
**2,3-butanedione**	Streptococcus	pneumoniae	10.1016/j.jchromb.2018.08.032
**2,3-butanedione**	Streptococcus	pneumoniae	10.1099/mic.0.062687-0
**2,3-diethyl-5-methylpyrazine**	Aspergillus	fumigatus	10.1128/EC.00074-14
**2,3-dihydroxy-3-methylpentanoic acid**	Streptococcus	pneumoniae	10.3390/metabo11110773
**2,3-dimethyl-5-pentylpyrazine**	Pseudomonas	aeruginosa	10.1088/1752-7155/10/1/016002
**2,3-dimethylpyrazine**	Staphylococcus	aureus	10.3390/metabo10090347
**2,3-heptanedione**	Pseudomonas	aeruginosa	10.1016/j.jchromb.2012.05.038

**2,3-heptanedione**	Staphylococcus	aureus	10.3390/metabo10090347
**2,3-heptanedione**	Staphylococcus	aureus	10.3390/metabo10090347
**2,3-heptanedione**	Staphylococcus	epidermidis	10.3390/metabo10090347
**2,3-heptanedione**	Staphylococcus	epidermidis	10.3390/metabo10090347
**2,3-heptanedione**	Staphylococcus	epidermidis	10.3390/metabo10090347
**2,3-heptanedione**	Proteus	mirabilis	10.1007/s00253-012-3924-4
**2,3-hexanedione**	Pseudomonas	aeruginosa	10.1088/1752-7155/10/4/047102
**2,3-hexanedione**	Klebsiella	pneumoniae	10.1088/1752-7155/10/2/027101
**2,3-pentanedione**	Pseudomonas	aeruginosa	10.1088/1752-7155/10/4/047102
**2,3-pentanedione**	Streptococcus	pneumoniae	10.1016/j.jchromb.2018.08.032
**2,3-pentanedione**	Streptococcus	pneumoniae	10.1016/j.jchromb.2018.08.032
**2,3-pentanedione**	Streptococcus	pneumoniae	10.1016/j.jchromb.2018.08.032
**2,3-pentanedione**	Streptococcus	pneumoniae	10.1016/j.jchromb.2018.08.032
**2,3-pentanedione**	Streptococcus	pneumoniae	10.1016/j.jchromb.2018.08.032
**2,3-pentanedione**	Streptococcus	pneumoniae	10.1016/j.jchromb.2018.08.032
**2,3-pentanedione**	Streptococcus	pneumoniae	10.1016/j.jchromb.2018.08.032
**2,4,6,8-tetramethyl-1-undecene**	Staphylococcus	aureus	10.3390/metabo10090347
**2,4,6,8-tetramethyl-1-undecene**	Staphylococcus	epidermidis	10.3390/metabo10090347
**2,4,6,8-tetramethyl-1-undecene**	Staphylococcus	epidermidis	10.3390/metabo10090347
**2,4,6-trimethylpyridine**	Pseudomonas	aeruginosa	10.1088/1752-7155/10/4/047102
**2,4-bis(1,1-Dimethyl)-phenol**	Pseudomonas	aeruginosa	10.1128/mSphere.00843-20
**2,4-bis(1,1-Dimethyl)-phenol**	Pseudomonas	aeruginosa	10.1128/mSphere.00843-20
**2,4-diacetylphloroglucinol**	Aspergillus	fumigatus	10.1128/EC.00074-14
**2,4-dimethyl-1-heptene**	Pseudomonas	aeruginosa	10.1088/1752-7155/10/4/047102
**2,4-dimethylbenzaldehyde**	Escherichia	coli	10.1111/jam.15716
**2,4-dimethylbenzaldehyde**	Escherichia	coli	10.1111/jam.15716
**2,4-dimethylfuran**	Pseudomonas	aeruginosa	10.1088/1752-7155/10/4/047102
**2,4-dimethylfuran**	Pseudomonas	aeruginosa	10.1128/mSphere.00843-20
**2,4-dimethylfuran**	Candida	albicans	10.1111/myc.12442
**2,4-dimethylheptan-1-ol**	Pseudomonas	aeruginosa	10.3389/fmicb.2021.693075
**2,4-dimethylheptan-1-ol**	Pseudomonas	aeruginosa	10.3389/fmicb.2021.693075
**2,4-dimethylheptan-1-ol**	Escherichia	coli	10.3389/fmicb.2021.693075
**2,4-dimethylheptan-1-ol**	Escherichia	coli	10.3389/fmicb.2021.693075
**2,4-dimethylheptan-1-ol**	Escherichia	coli	10.3389/fmicb.2021.693075
**2,4-dimethylheptan-1-ol**	Escherichia	coli	10.3389/fmicb.2021.693075
**2,4-dimethylheptan-1-ol**	Escherichia	coli	10.3389/fmicb.2021.693075
**2,4-dimethylheptan-1-ol**	Escherichia	coli	10.3389/fmicb.2021.693075
**2,4-dimethylheptane**	Escherichia	coli	10.3390/antibiotics9110797
**2,4-dimethylpyrrole**	Staphylococcus	epidermidis	10.3390/metabo10090347
**2,4-dimethylpyrrole**	Staphylococcus	epidermidis	10.3390/metabo10090347
**2,4-di-tert-butylphenol**	Candida	albicans	10.1038/srep27441
**2,4-di-tert-butylphenol**	Penicillium	chrysogenum	10.1038/srep27441
**2,4-di-tert-butylphenol**	Escherichia	coli	10.1111/jam.15716

**2,4-di-tert-butylphenol**	Escherichia	coli	10.1111/jam.15716
**2,4-di-tert-butylphenol**	Aspergillus	niger	10.1038/srep27441
**2,5-dimethyl-3-(2-methylpropyl)pyrazine**	Klebsiella	pneumoniae	10.1111/jam.13372
**2,5-dimethyldecane**	Mycobacterium	tuberculosis	10.1088/1752-7163/aacd18
**2,5-dimethylfuran**	Candida	albicans	10.3390/metabo12050432
**2,5-dimethylfuran**	Escherichia	coli	10.3390/antibiotics9110797
**2,5-dimethylpyrazine**	Pseudomonas	aeruginosa	10.1016/j.jchromb.2009.05.028
**2,5-dimethylpyrazine**	Pseudomonas	aeruginosa	10.1016/j.jchromb.2012.05.038
**2,5-dimethylpyrazine**	Pseudomonas	aeruginosa	10.1038/s41598-020-74909-w
**2,5-dimethylpyrazine**	Pseudomonas	aeruginosa	10.1038/s41598-020-74909-w
**2,5-dimethylpyrazine**	Pseudomonas	aeruginosa	10.1128/mSphere.00843-20
**2,5-dimethylpyrazine**	Staphylococcus	aureus	10.1038/s41598-020-74909-w
**2,5-dimethylpyrazine**	Staphylococcus	aureus	10.1038/s41598-020-74909-w
**2,5-dimethylpyrazine**	Staphylococcus	aureus	10.3390/metabo10090347
**2,5-dimethylpyrazine**	Staphylococcus	aureus	10.3390/metabo10090347
**2,5-dimethylpyrazine**	Staphylococcus	aureus	10.3390/metabo10090347
**2,5-dimethylpyrazine**	Acinetobacter	baumannii	10.1088/1752-7155/10/2/027102
**2,5-dimethylpyrazine**	Escherichia	coli	10.1038/s41598-020-74909-w
**2,5-dimethylpyrazine**	Escherichia	coli	10.1038/s41598-020-74909-w
**2,5-dimethylpyrazine**	Escherichia	coli	10.1111/jam.15716
**2,5-dimethylpyrazine**	Escherichia	coli	10.1111/jam.15716
**2,5-dimethylpyrazine**	Escherichia	coli	10.3390/antibiotics9110797
**2,5-dimethylpyrazine**	Staphylococcus	epidermidis	10.1038/s41598-020-74909-w
**2,5-dimethylpyrazine**	Staphylococcus	epidermidis	10.1038/s41598-020-74909-w
**2,5-dimethylpyrazine**	Staphylococcus	epidermidis	10.3390/metabo10090347
**2,5-dimethylpyrazine**	Staphylococcus	epidermidis	10.3390/metabo10090347
**2,5-dimethylpyrazine**	Staphylococcus	epidermidis	10.3390/metabo10090347
**2,5-dimethylpyrazine**	Staphylococcus	epidermidis	10.3390/metabo10090347
**2,6,11-trimethyldodecane**	Klebsiella	pneumoniae	10.1111/jam.13372
**2,6-Bis(1,1-dimethylethyl)-2,5-cyclohexadiene-1,4**	Escherichia	coli	10.1111/jam.15716
**2,6-diethylpyrazine**	Escherichia	coli	10.1111/jam.15716
**2,6-dimethyl-3-isopentylpyrazine**	Pseudomonas	aeruginosa	10.1007/s00253-012-3924-4
**2,6-dimethyl-3-isopentylpyrazine**	Streptococcus	agalactiae	10.1007/s00253-012-3924-4
**2,6-dimethyl-3-isopentylpyrazine**	Staphylococcus	aureus	10.3390/metabo10090347
**2,6-dimethyl-3-isopentylpyrazine**	Staphylococcus	aureus	10.3390/metabo10090347
**2,6-dimethyl-3-isopentylpyrazine**	Enterobacter	cloacae	10.1007/s00253-012-3924-4
**2,6-dimethyl-3-isopentylpyrazine**	Staphylococcus	epidermidis	10.3390/metabo10090347
**2,6-dimethyl-3-isopentylpyrazine**	Proteus	mirabilis	10.1007/s00253-012-3924-4
**2,6-dimethylbicyclo[3.2.1]octane**	Candida	albicans	10.1038/srep27441
**2,6-dimethylbicyclo[3.2.1]octane**	Penicillium	chrysogenum	10.1038/srep27441
**2,6-dimethylbicyclo[3.2.1]octane**	Aspergillus	niger	10.1038/srep27441
**2,6-dimethylheptadecane**	Mycobacterium	tuberculosis	10.1088/1752-7163/aacd18
**2-acetylthiazole**	Aspergillus	fumigatus	10.1088/1752-7155/10/1/016002

**2-acetylthiazole**	Proteus	mirabilis	10.1007/s00253-012-3924-4
**2-acetylthiophene**	Streptococcus	pneumoniae	10.3390/metabo11110773
**2'-aminoacetophenone**	Escherichia	coli	10.1002/jssc.201800684
**2-aminoacetophenone**	Pseudomonas	aeruginosa	10.1016/j.jchromb.2009.05.028
**2-aminoacetophenone**	Pseudomonas	aeruginosa	10.1016/j.jchromb.2012.05.038
**2-aminoacetophenone**	Pseudomonas	aeruginosa	10.1016/j.mimet.2010.12.001
**2-aminoacetophenone**	Pseudomonas	aeruginosa	10.1016/j.mimet.2010.12.001
**2-aminoacetophenone**	Pseudomonas	aeruginosa	10.1128/JCM.00392-10
**2-aminoacetophenone**	Pseudomonas	aeruginosa	10.1128/JCM.12.4.521-526.1980
**2-aminoacetophenone**	Pseudomonas	aeruginosa	10.1128/JCM.12.4.521-526.1980
**2-aminoacetophenone**	Pseudomonas	aeruginosa	10.1128/JCM.12.4.521-526.1980
**2-aminoacetophenone**	Pseudomonas	aeruginosa	10.1128/JCM.12.4.521-526.1980
**2-aminoacetophenone**	Pseudomonas	aeruginosa	10.1128/JCM.12.4.521-526.1980
**2-aminoacetophenone**	Pseudomonas	aeruginosa	10.1128/JCM.12.4.521-526.1980
**2-aminoacetophenone**	Pseudomonas	aeruginosa	10.1128/JCM.12.4.521-526.1980
**2-aminoacetophenone**	Pseudomonas	aeruginosa	10.1128/JCM.12.4.521-526.1980
**2-aminoacetophenone**	Pseudomonas	aeruginosa	10.1128/JCM.12.4.521-526.1980
**2-aminoacetophenone**	Pseudomonas	aeruginosa	10.1128/JCM.12.4.521-526.1980
**2-aminoacetophenone**	Pseudomonas	aeruginosa	10.1128/JCM.12.4.521-526.1980
**2-aminoacetophenone**	Pseudomonas	aeruginosa	10.1186/1471-2466-10-56
**2-aminoacetophenone**	Pseudomonas	aeruginosa	10.1186/1471-2466-10-56
**2-aminoacetophenone**	Pseudomonas	aeruginosa	10.1186/1471-2466-10-56
**2-aminoacetophenone**	Pseudomonas	aeruginosa	10.1186/1471-2466-10-56
**2-aminoacetophenone**	Pseudomonas	aeruginosa	10.1186/1471-2466-10-56
**2-aminoacetophenone**	Pseudomonas	aeruginosa	10.3389/fmicb.2021.693075
**2-aminoacetophenone**	Pseudomonas	aeruginosa	10.3389/fmicb.2021.693075
**2-aminoacetophenone**	Pseudomonas	aeruginosa	10.3389/fmicb.2021.693075
**2-aminoacetophenone**	Pseudomonas	aeruginosa	10.3389/fmicb.2021.693075
**2-aminoacetophenone**	Pseudomonas	aeruginosa	10.3389/fmicb.2021.693075
**2-aminoacetophenone**	Pseudomonas	aeruginosa	10.3389/fmicb.2021.693075
**2-aminoacetophenone**	Staphylococcus	aureus	10.1016/j.mimet.2005.09.003
**2-aminoacetophenone**	Staphylococcus	aureus	10.1016/j.mimet.2010.12.001
**2-aminoacetophenone**	Staphylococcus	aureus	10.1016/j.mimet.2010.12.001
**2-aminoacetophenone**	Burkholderia	cepacia	10.1016/j.mimet.2010.12.001
**2-aminoacetophenone**	Escherichia	coli	10.1016/j.mimet.2005.09.003
**2-aminoacetophenone**	Escherichia	coli	10.1016/j.mimet.2005.09.016
**2-aminoacetophenone**	Escherichia	coli	10.1016/j.mimet.2010.12.001
**2-aminoacetophenone**	Escherichia	coli	10.1016/j.mimet.2010.12.001
**2-aminoacetophenone**	Enterococcus	faecalis	10.1016/j.mimet.2010.12.001
**2-aminoacetophenone**	Proteus	mirabilis	10.1016/j.mimet.2010.12.001
**2-aminoacetophenone**	Proteus	mirabilis	10.1016/j.mimet.2010.12.001
**2-aminoacetophenone**	Streptococcus	pneumoniae	10.1016/j.mimet.2005.09.003
**2-aminoacetophenone**	Streptococcus	pneumoniae	10.1016/j.mimet.2005.09.016

**2-butanol**	Pseudomonas	aeruginosa	10.1016/j.jchromb.2012.05.038
**2-butanol**	Pseudomonas	aeruginosa	10.1016/S0378-4347(00)80760-4
**2-butanol**	Pseudomonas	aeruginosa	10.1016/S0378-4347(00)80760-4
**2-butanol**	Pseudomonas	aeruginosa	10.1038/s41598-020-74909-w
**2-butanol**	Pseudomonas	aeruginosa	10.1038/s41598-020-74909-w
**2-butanol**	Pseudomonas	aeruginosa	10.1186/1471-2180-12-113
**2-butanol**	Staphylococcus	aureus	10.1016/S0378-4347(00)80760-4
**2-butanol**	Stenotrophomonas	maltophilia	10.1088/1752-7155/9/2/027104
**2-butanol**	Klebsiella	pneumoniae	10.1016/S0378-4347(00)80760-4
**2-butanol**	Klebsiella	pneumoniae	10.1016/S0378-4347(00)80760-4
**2-butanol**	Stenotrophomonas	rhizophila	10.1088/1752-7155/9/2/027104
**2-butanone**	Pseudomonas	aeruginosa	10.1016/j.jchromb.2012.05.038
**2-butanone**	Pseudomonas	aeruginosa	10.1016/S0378-4347(00)80760-4
**2-butanone**	Pseudomonas	aeruginosa	10.1016/S0378-4347(00)80760-4
**2-butanone**	Pseudomonas	aeruginosa	10.1088/1752-7155/10/4/047102
**2-butanone**	Pseudomonas	aeruginosa	10.1088/1752-7155/8/2/027106
**2-butanone**	Pseudomonas	aeruginosa	10.1088/1752-7155/8/2/027106
**2-butanone**	Pseudomonas	aeruginosa	10.1128/JCM.12.4.521-526.1980
**2-butanone**	Pseudomonas	aeruginosa	10.1128/JCM.12.4.521-526.1980
**2-butanone**	Pseudomonas	aeruginosa	10.1128/JCM.12.4.521-526.1980
**2-butanone**	Pseudomonas	aeruginosa	10.1128/JCM.12.4.521-526.1980
**2-butanone**	Pseudomonas	aeruginosa	10.1128/JCM.12.4.521-526.1980
**2-butanone**	Pseudomonas	aeruginosa	10.1128/JCM.12.4.521-526.1980
**2-butanone**	Pseudomonas	aeruginosa	10.1128/JCM.12.4.521-526.1980
**2-butanone**	Pseudomonas	aeruginosa	10.1128/JCM.12.4.521-526.1980
**2-butanone**	Pseudomonas	aeruginosa	10.1128/JCM.12.4.521-526.1980
**2-butanone**	Pseudomonas	aeruginosa	10.1128/JCM.12.4.521-526.1980
**2-butanone**	Pseudomonas	aeruginosa	10.1128/JCM.12.4.521-526.1980
**2-butanone**	Pseudomonas	aeruginosa	10.1128/mSphere.00843-20
**2-butanone**	Pseudomonas	aeruginosa	10.1186/1471-2180-12-113
**2-butanone**	Staphylococcus	aureus	10.1016/S0378-4347(00)80760-4
**2-butanone**	Staphylococcus	aureus	10.1038/s41598-020-74909-w
**2-butanone**	Staphylococcus	aureus	10.1038/s41598-020-74909-w
**2-butanone**	Staphylococcus	aureus	10.3390/metabo10090347
**2-butanone**	Mycobacterium	bovis	10.1111/j.1574-6968.2011.02493.[illegible]
**2-butanone**	Mycobacterium	bovis	10.1371/journal.pone.0194348
**2-butanone**	Escherichia	coli	10.1038/s41598-020-74909-w
**2-butanone**	Escherichia	coli	10.1038/s41598-020-74909-w
**2-butanone**	Escherichia	coli	10.3390/antibiotics9110797
**2-butanone**	Escherichia	coli	10.3390/antibiotics9110797
**2-butanone**	Staphylococcus	epidermidis	10.1038/s41598-020-74909-w
**2-butanone**	Stenotrophomonas	maltophilia	10.1088/1752-7155/9/2/027104
**2-butanone**	Klebsiella	pneumoniae	10.1016/S0378-4347(00)80760-4

**2-butanone**	Klebsiella	pneumoniae	10.1016/S0378-4347(00)80760-4
**2-butanone**	Klebsiella	pneumoniae	10.1088/1752-7155/8/2/027106
**2-butanone**	Streptococcus	pneumoniae	10.1099/mic.0.062687-0
**2-butanone**	Stenotrophomonas	rhizophila	10.1088/1752-7155/9/2/027104
**2-butoxyethanol**	Staphylococcus	epidermidis	10.3390/metabo10090347
**2-butoxyethyl acetate**	Escherichia	coli	10.3390/antibiotics9110797
**2-butyl-1-octanol**	Pseudomonas	aeruginosa	10.1128/mSphere.00843-20
**2-butyl-1-octanol**	Staphylococcus	aureus	10.3390/metabo10090347
**2-butyl-3-methylpyrazine**	Pseudomonas	aeruginosa	10.1016/j.jchromb.2012.05.038
**2-butylfuran**	Pseudomonas	aeruginosa	10.1128/mSphere.00843-20
**2-cyclopenten-1-one**	Pseudomonas	aeruginosa	10.1007/s11306-018-1357-5
**2-cyclopenten-1-one**	Pseudomonas	aeruginosa	10.1088/1752-7163/aa8efc
**2-cyclopenten-1-one**	Staphylococcus	aureus	10.1088/1752-7163/aa8efc
**2-cyclopenten-1-one**	Enterobacter	cloacae	10.1007/s11306-018-1357-5
**2-cyclopenten-1-one**	Escherichia	coli	10.1088/1752-7163/aa8efc
**2-decanone**	Pseudomonas	aeruginosa	10.1088/1752-7155/10/4/047102
**2-decanone**	Pseudomonas	aeruginosa	10.1128/mSphere.00843-20
**2-decanone**	Staphylococcus	aureus	10.3390/metabo10090347
**2-decanone**	Staphylococcus	aureus	10.3390/metabo10090347
**2-decanone**	Enterobacter	cloacae	10.1007/s00253-012-3924-4
**2-decanone**	Escherichia	coli	10.1002/jssc.201800684
**2-decanone**	Escherichia	coli	10.1007/s00253-012-3924-4
**2-decanone**	Escherichia	coli	10.1038/s41598-020-74909-w
**2-decanone**	Escherichia	coli	10.1038/s41598-020-74909-w
**2-decanone**	Staphylococcus	epidermidis	10.3390/metabo10090347
**2-decanone**	Staphylococcus	epidermidis	10.3390/metabo10090347
**2-decanone**	Serratia	marcescens	10.1007/s00253-012-3924-4
**2-dodecanone**	Pseudomonas	aeruginosa	10.1016/j.jchromb.2012.05.038
**2-dodecanone**	Pseudomonas	aeruginosa	10.1128/mSphere.00843-20
**2-dodecanone**	Escherichia	coli	10.3389/fmicb.2021.693075
**2-dodecanone**	Escherichia	coli	10.3389/fmicb.2021.693075
**2-dodecanone**	Escherichia	coli	10.3389/fmicb.2021.693075
**2-dodecanone**	Escherichia	coli	10.3390/antibiotics9110797
**2-dodecanone**	Staphylococcus	epidermidis	10.3390/metabo10090347
**2-dodecanone**	Staphylococcus	epidermidis	10.3390/metabo10090347
**2-ethyl-3-(methylthio)pyrazine**	Pseudomonas	aeruginosa	10.1016/j.jchromb.2012.05.038
**2-ethyl-3,5-dimethylpyrazine**	Klebsiella	pneumoniae	10.1111/jam.13372
**2-ethyl-5-methylpyrazine**	Pseudomonas	aeruginosa	10.1038/s41598-020-74909-w
**2-ethyl-5-methylpyrazine**	Pseudomonas	aeruginosa	10.1128/mSphere.00843-20
**2-ethyl-5-methylpyrazine**	Staphylococcus	aureus	10.1038/s41598-020-74909-w
**2-ethyl-5-methylpyrazine**	Staphylococcus	aureus	10.1038/s41598-020-74909-w
**2-ethyl-6-methylpyrazine**	Staphylococcus	aureus	10.1038/s41598-020-74909-w
**2-ethyl-6-methylpyrazine**	Staphylococcus	aureus	10.1038/s41598-020-74909-w

**2-ethyl-6-methylpyrazine**	Staphylococcus	aureus	10.3390/metabo10090347
**2-ethyl-6-methylpyrazine**	Staphylococcus	epidermidis	10.1038/s41598-020-74909-w
**2-ethyl-6-methylpyrazine**	Staphylococcus	epidermidis	10.3390/metabo10090347
**2-ethylacrolein**	Staphylococcus	aureus	10.1186/1471-2180-12-113
**2-ethylacrolein**	Staphylococcus	epidermidis	10.3390/metabo10090347
**2-ethylhexan-1-ol**	Pseudomonas	aeruginosa	10.1007/s00253-013-4762-8
**2-ethylhexan-1-ol**	Pseudomonas	aeruginosa	10.1007/s00253-013-4762-8
**2-ethylhexan-1-ol**	Candida	albicans	10.1038/srep27441
**2-ethylhexan-1-ol**	Penicillium	chrysogenum	10.1038/srep27441
**2-ethylhexan-1-ol**	Escherichia	coli	10.1111/jam.15716
**2-ethylhexan-1-ol**	Escherichia	coli	10.1111/jam.15716
**2-ethylhexan-1-ol**	Escherichia	coli	10.3390/antibiotics9110797
**2-ethylhexan-1-ol**	Aspergillus	niger	10.1038/srep27441
**2-ethylhexanal**	Aspergillus	fumigatus	10.1088/1752-7155/6/1/016002
**2-ethylhexanal**	Aspergillus	fumigatus	10.1088/1752-7155/6/1/016002
**2-ethylhexanal**	Aspergillus	fumigatus	10.1088/1752-7155/6/1/016002
**2-ethylhexanal**	Aspergillus	fumigatus	10.1088/1752-7155/6/1/016002
**2-ethylhexanal**	Aspergillus	fumigatus	10.1088/1752-7155/6/1/016002
**2-ethylhexanal**	Aspergillus	fumigatus	10.1088/1752-7155/6/1/016002
**2-ethylhexanoic acid**	Escherichia	coli	10.1111/jam.15716
**2-ethylhexanoic acid**	Escherichia	coli	10.1111/jam.15716
**2-ethylhexyl acetate**	Aspergillus	fumigatus	10.1088/1752-7155/6/1/016002
**2-ethylhexyl acetate**	Aspergillus	fumigatus	10.1088/1752-7155/6/1/016002
**2-ethylhexyl acetate**	Aspergillus	fumigatus	10.1088/1752-7155/6/1/016002
**2-ethylhexyl acetate**	Aspergillus	fumigatus	10.1088/1752-7155/6/1/016002
**2-ethylhexyl acetate**	Aspergillus	fumigatus	10.1088/1752-7155/6/1/016002
**2-ethylhexyl vinyl ether**	Escherichia	coli	10.1111/jam.15716
**2-ethylhexyl vinyl ether**	Escherichia	coli	10.1111/jam.15716
**2-ethyl-p-xylene**	Candida	albicans	10.1038/srep27441
**2-ethyl-p-xylene**	Penicillium	chrysogenum	10.1038/srep27441
**2-ethyl-p-xylene**	Aspergillus	niger	10.1038/srep27441
**2-heptanol**	Pseudomonas	aeruginosa	10.1186/s13568-022-01367-0
**2-heptanol**	Mycobacterium	bovis	10.1371/journal.pone.0194348
**2-heptanone**	Pseudomonas	aeruginosa	10.1016/j.jchromb.2012.05.038
**2-heptanone**	Pseudomonas	aeruginosa	10.1016/S0378-4347(00)80760-4
**2-heptanone**	Pseudomonas	aeruginosa	10.1016/S0378-4347(00)80760-4
**2-heptanone**	Pseudomonas	aeruginosa	10.1088/1752-7155/10/4/047102
**2-heptanone**	Pseudomonas	aeruginosa	10.1088/1752-7155/8/2/027106
**2-heptanone**	Pseudomonas	aeruginosa	10.1088/1752-7155/8/2/027106
**2-heptanone**	Pseudomonas	aeruginosa	10.1186/1471-2180-12-113
**2-heptanone**	Pseudomonas	aeruginosa	10.3389/fmicb.2021.693075
**2-heptanone**	Pseudomonas	aeruginosa	10.3389/fmicb.2021.693075
**2-heptanone**	Pseudomonas	aeruginosa	10.3389/fmicb.2021.693075

**2-heptanone**	Pseudomonas	aeruginosa	10.3389/fmicb.2021.693075
**2-heptanone**	Pseudomonas	aeruginosa	10.3389/fmicb.2021.693075
**2-heptanone**	Pseudomonas	aeruginosa	10.3389/fmicb.2021.693075
**2-heptanone**	Staphylococcus	aureus	10.1002/jobm.201600505
**2-heptanone**	Staphylococcus	aureus	10.1016/S0378-4347(00)80760-4
**2-heptanone**	Staphylococcus	aureus	10.1038/s41598-020-74909-w
**2-heptanone**	Staphylococcus	aureus	10.1038/s41598-020-74909-w
**2-heptanone**	Staphylococcus	aureus	10.1088/1752-7155/8/2/027106
**2-heptanone**	Staphylococcus	aureus	10.3389/fmicb.2021.693075
**2-heptanone**	Staphylococcus	aureus	10.3389/fmicb.2021.693075
**2-heptanone**	Escherichia	coli	10.1002/jobm.201600505
**2-heptanone**	Escherichia	coli	10.1007/s00216-009-2758-0
**2-heptanone**	Escherichia	coli	10.1038/s41598-020-74909-w
**2-heptanone**	Escherichia	coli	10.1038/s41598-020-74909-w
**2-heptanone**	Escherichia	coli	10.1088/1752-7155/8/2/027106
**2-heptanone**	Escherichia	coli	10.1088/1752-7155/8/2/027106
**2-heptanone**	Escherichia	coli	10.3389/fmicb.2021.693075
**2-heptanone**	Escherichia	coli	10.3389/fmicb.2021.693075
**2-heptanone**	Escherichia	coli	10.3389/fmicb.2021.693075
**2-heptanone**	Escherichia	coli	10.3389/fmicb.2021.693075
**2-heptanone**	Escherichia	coli	10.3389/fmicb.2021.693075
**2-heptanone**	Escherichia	coli	10.3389/fmicb.2021.693075
**2-heptanone**	Escherichia	coli	10.3390/antibiotics9110797
**2-heptanone**	Staphylococcus	epidermidis	10.1038/s41598-020-74909-w
**2-heptanone**	Staphylococcus	epidermidis	10.1038/s41598-020-74909-w
**2-heptanone**	Aspergillus	fumigatus	10.1088/1752-7155/6/1/016002
**2-heptanone**	Aspergillus	fumigatus	10.1088/1752-7155/6/1/016002
**2-heptanone**	Aspergillus	fumigatus	10.1088/1752-7155/6/1/016002
**2-heptanone**	Serratia	liquefaciens	10.1016/S0045-6535(97)00209-9
**2-heptanone**	Klebsiella	pneumoniae	10.1016/S0378-4347(00)80760-4
**2-heptanone**	Klebsiella	pneumoniae	10.1016/S0378-4347(00)80760-4
**2-heptanone**	Klebsiella	pneumoniae	10.1088/1752-7155/10/2/027101
**2-hexanone**	Pseudomonas	aeruginosa	10.1016/j.jchromb.2012.05.038
**2-hexanone**	Staphylococcus	aureus	10.3389/fmicb.2021.693075
**2-hexanone**	Escherichia	coli	10.3389/fmicb.2021.693075
**2-hexanone**	Escherichia	coli	10.3389/fmicb.2021.693075
**2-hexanone**	Escherichia	coli	10.3389/fmicb.2021.693075
**2-hexanone**	Escherichia	coli	10.3389/fmicb.2021.693075
**2-hexanone**	Escherichia	coli	10.3389/fmicb.2021.693075
**2-hexanone**	Escherichia	coli	10.3389/fmicb.2021.693075
**2-hexene**	Mycobacterium	tuberculosis	10.1016/j.tube.2006.03.004
**2-hexene**	Mycobacterium	tuberculosis	10.1016/j.tube.2006.03.004
**2-hydroxy-3-pentanone**	Staphylococcus	aureus	10.1038/s41598-020-74909-w

**2-isobutyl-3-methylpyrazine**	Aspergillus	fumigatus	10.1128/EC.00074-14
**2-isopropyl-3-methylpyrazine**	Pseudomonas	aeruginosa	10.1016/j.jchromb.2012.05.038
**2-isopropyl-5-methylpyrazine**	Aspergillus	fumigatus	10.1128/EC.00074-14
**2-methoxy-5-methylthiophene**	Pseudomonas	aeruginosa	10.1186/1471-2180-12-113
**2-methoxybiphenyl**	Mycobacterium	abscessus	10.1016/j.tube.2008.01.002
**2-methoxybiphenyl**	Mycobacterium	avium	10.1016/j.tube.2008.01.002
**2-methoxybiphenyl**	Mycobacterium	bovis	10.1016/j.tube.2008.01.002
**2-methoxybiphenyl**	Mycobacterium	bovis	10.1016/j.tube.2008.01.002
**2-methoxybiphenyl**	Mycobacterium	chelonae	10.1016/j.tube.2008.01.002
**2-methoxybiphenyl**	Mycobacterium	fortuitum	10.1016/j.tube.2008.01.002
**2-methoxybiphenyl**	Mycobacterium	tuberculosis	10.1016/j.tube.2008.01.002
**2-methoxybiphenyl**	Mycobacterium	tuberculosis	10.1016/j.tube.2008.01.002
**2-methyl-1-butanol**	Candida	albicans	10.1111/myc.12442
**2-methyl-1-butanol**	Mycobacterium	bovis	10.1111/j.1574-6968.2011.02493.[illegible]
**2-methyl-1-butanol**	Escherichia	coli	10.1128/AEM.02069-07
**2-methyl-1-butanol**	Staphylococcus	epidermidis	10.3390/metabo10090347
**2-methyl-1-butanol**	Stenotrophomonas	maltophilia	10.1088/1752-7155/9/2/027104
**2-methyl-1-butanol**	Stenotrophomonas	rhizophila	10.1088/1752-7155/9/2/027104
**2-methyl-1-butene**	Streptococcus	pneumoniae	10.1099/mic.0.062687-0
**2-methyl-1h-pyrrole**	Pseudomonas	aeruginosa	10.1038/s41598-020-74909-w
**2-methyl-1h-pyrrole**	Pseudomonas	aeruginosa	10.3389/fmicb.2021.693075
**2-methyl-1h-pyrrole**	Pseudomonas	aeruginosa	10.3389/fmicb.2021.693075
**2-methyl-1h-pyrrole**	Pseudomonas	aeruginosa	10.3389/fmicb.2021.693075
**2-methyl-1h-pyrrole**	Pseudomonas	aeruginosa	10.3389/fmicb.2021.693075
**2-methyl-1h-pyrrole**	Pseudomonas	aeruginosa	10.3389/fmicb.2021.693075
**2-methyl-1-pentanol**	Pseudomonas	aeruginosa	10.3389/fmicb.2021.693075
**2-methyl-1-pentanol**	Pseudomonas	aeruginosa	10.3389/fmicb.2021.693075
**2-methyl-1-pentanol**	Pseudomonas	aeruginosa	10.3389/fmicb.2021.693075
**2-methyl-1-pentanol**	Pseudomonas	aeruginosa	10.3389/fmicb.2021.693075
**2-methyl-1-pentanol**	Pseudomonas	aeruginosa	10.3389/fmicb.2021.693075
**2-methyl-1-pentanol**	Pseudomonas	aeruginosa	10.3389/fmicb.2021.693075
**2-methyl-1-pentanol**	Candida	albicans	10.3390/metabo12050432
**2-methyl-1-pentanol**	Escherichia	coli	10.3389/fmicb.2021.693075
**2-methyl-1-pentanol**	Escherichia	coli	10.3389/fmicb.2021.693075
**2-methyl-1-pentanol**	Escherichia	coli	10.3389/fmicb.2021.693075
**2-methyl-1-pentanol**	Escherichia	coli	10.3389/fmicb.2021.693075
**2-methyl-1-pentanol**	Escherichia	coli	10.3389/fmicb.2021.693075
**2-methyl-1-pentanol**	Escherichia	coli	10.3389/fmicb.2021.693075
**2-methyl-1-propanol**	Pseudomonas	aeruginosa	10.1016/S0378-4347(00)80760-4
**2-methyl-1-propanol**	Pseudomonas	aeruginosa	10.1016/S0378-4347(00)80760-4
**2-methyl-1-propanol**	Candida	albicans	10.1111/myc.12442
**2-methyl-1-propanol**	Candida	albicans	10.3390/metabo12050432
**2-methyl-1-propanol**	Candida	albicans	10.3390/metabo12050432

**2-methyl-1-propanol**	Staphylococcus	aureus	10.1016/S0378-4347(00)80760-4
**2-methyl-1-propanol**	Staphylococcus	aureus	10.1038/s41598-020-74909-w
**2-methyl-1-propanol**	Staphylococcus	aureus	10.1038/s41598-020-74909-w
**2-methyl-1-propanol**	Staphylococcus	aureus	10.1186/1471-2180-12-113
**2-methyl-1-propanol**	Staphylococcus	aureus	10.3389/fmicb.2021.693075
**2-methyl-1-propanol**	Staphylococcus	aureus	10.3389/fmicb.2021.693075
**2-methyl-1-propanol**	Staphylococcus	aureus	10.3389/fmicb.2021.693075
**2-methyl-1-propanol**	Staphylococcus	aureus	10.3389/fmicb.2021.693075
**2-methyl-1-propanol**	Staphylococcus	aureus	10.3389/fmicb.2021.693075
**2-methyl-1-propanol**	Staphylococcus	aureus	10.3389/fmicb.2021.693075
**2-methyl-1-propanol**	Mycobacterium	bovis	10.1111/j.1574-6968.2011.02493.[illegible]
**2-methyl-1-propanol**	Mycobacterium	bovis	10.1371/journal.pone.0194348
**2-methyl-1-propanol**	Enterobacter	cloacae	10.1007/s11306-018-1357-5
**2-methyl-1-propanol**	Enterobacter	cloacae	10.1016/S0045-6535(97)00209-9
**2-methyl-1-propanol**	Escherichia	coli	10.3389/fmicb.2021.693075
**2-methyl-1-propanol**	Escherichia	coli	10.3389/fmicb.2021.693075
**2-methyl-1-propanol**	Escherichia	coli	10.3389/fmicb.2021.693075
**2-methyl-1-propanol**	Escherichia	coli	10.3389/fmicb.2021.693075
**2-methyl-1-propanol**	Escherichia	coli	10.3389/fmicb.2021.693075
**2-methyl-1-propanol**	Escherichia	coli	10.3389/fmicb.2021.693075
**2-methyl-1-propanol**	Escherichia	coli	10.3390/antibiotics9110797
**2-methyl-1-propanol**	Stenotrophomonas	maltophilia	10.1088/1752-7155/9/2/027104
**2-methyl-1-propanol**	Klebsiella	pneumoniae	10.1016/S0378-4347(00)80760-4
**2-methyl-1-propanol**	Klebsiella	pneumoniae	10.1016/S0378-4347(00)80760-4
**2-methyl-1-propanol**	Stenotrophomonas	rhizophila	10.1088/1752-7155/9/2/027104
**2-methyl-2-butanol**	Pseudomonas	aeruginosa	10.3389/fmicb.2021.693075
**2-methyl-2-butanol**	Pseudomonas	aeruginosa	10.3389/fmicb.2021.693075
**2-methyl-2-butanol**	Pseudomonas	aeruginosa	10.3389/fmicb.2021.693075
**2-methyl-2-butanol**	Staphylococcus	aureus	10.3389/fmicb.2021.693075
**2-methyl-2-butanol**	Staphylococcus	aureus	10.3389/fmicb.2021.693075
**2-methyl-2-butanol**	Staphylococcus	aureus	10.3389/fmicb.2021.693075
**2-methyl-2-butanol**	Staphylococcus	aureus	10.3389/fmicb.2021.693075
**2-methyl-2-butanol**	Staphylococcus	aureus	10.3389/fmicb.2021.693075
**2-methyl-2-butanol**	Staphylococcus	aureus	10.3389/fmicb.2021.693075
**2-methyl-2-butanol**	Escherichia	coli	10.3389/fmicb.2021.693075
**2-methyl-2-butanol**	Escherichia	coli	10.3389/fmicb.2021.693075
**2-methyl-2-butanol**	Escherichia	coli	10.3389/fmicb.2021.693075
**2-methyl-2-butanol**	Escherichia	coli	10.3389/fmicb.2021.693075
**2-methyl-2-butanol**	Escherichia	coli	10.3389/fmicb.2021.693075
**2-methyl-2-butanol**	Escherichia	coli	10.3389/fmicb.2021.693075
**2-methyl-2-butanol**	Staphylococcus	epidermidis	10.3390/metabo10090347
**2-methyl-2-butene**	Pseudomonas	aeruginosa	10.1186/1471-2180-12-113
**2-methyl-2-butene**	Haemophilus	influenzae	10.1099/mic.0.062687-0

**2-methyl-2-hexanol**	Klebsiella	pneumoniae	10.1111/jam.13372
**2-methyl-2-pentanol**	Pseudomonas	aeruginosa	10.3389/fmicb.2021.693075
**2-methyl-2-pentanol**	Pseudomonas	aeruginosa	10.3389/fmicb.2021.693075
**2-methyl-2-pentanol**	Pseudomonas	aeruginosa	10.3389/fmicb.2021.693075
**2-methyl-2-pentanol**	Pseudomonas	aeruginosa	10.3389/fmicb.2021.693075
**2-methyl-2-pentanol**	Pseudomonas	aeruginosa	10.3389/fmicb.2021.693075
**2-methyl-2-pentanol**	Pseudomonas	aeruginosa	10.3389/fmicb.2021.693075
**2-methyl-2-pentanol**	Staphylococcus	aureus	10.3389/fmicb.2021.693075
**2-methyl-2-pentanol**	Staphylococcus	aureus	10.3389/fmicb.2021.693075
**2-methyl-2-pentanol**	Escherichia	coli	10.3389/fmicb.2021.693075
**2-methyl-2-pentanol**	Escherichia	coli	10.3389/fmicb.2021.693075
**2-methyl-2-pentanol**	Escherichia	coli	10.3389/fmicb.2021.693075
**2-methyl-2-pentanol**	Escherichia	coli	10.3389/fmicb.2021.693075
**2-methyl-2-pentanol**	Escherichia	coli	10.3389/fmicb.2021.693075
**2-methyl-2-pentanol**	Escherichia	coli	10.3389/fmicb.2021.693075
**2-methyl-3-(2-propenyl)pyrazine**	Pseudomonas	aeruginosa	10.1088/1752-7155/10/1/016002
**2-methyl-3-(methylthio)furan**	Pseudomonas	aeruginosa	10.1186/s13568-022-01367-0
**2-methyl-3-buten-2-ol**	Pseudomonas	aeruginosa	10.1016/S0378-4347(00)80760-4
**2-methyl-3-buten-2-ol**	Pseudomonas	aeruginosa	10.1016/S0378-4347(00)80760-4
**2-methyl-3-buten-2-ol**	Pseudomonas	aeruginosa	10.1128/mSphere.00843-20
**2-methyl-3-buten-2-ol**	Staphylococcus	aureus	10.1016/S0378-4347(00)80760-4
**2-methyl-3-buten-2-ol**	Klebsiella	pneumoniae	10.1016/S0378-4347(00)80760-4
**2-methyl-3-buten-2-ol**	Klebsiella	pneumoniae	10.1016/S0378-4347(00)80760-4
**2-methyl-5-propylpyrazine**	Staphylococcus	aureus	10.3390/metabo10090347
**2-methyl-5-propylpyrazine**	Staphylococcus	aureus	10.3390/metabo10090347
**2-methyl-5-propylpyrazine**	Staphylococcus	epidermidis	10.3390/metabo10090347
**2-methyl-5-propylpyrazine**	Staphylococcus	epidermidis	10.3390/metabo10090347
**2-methyl-5-propylpyrazine**	Staphylococcus	epidermidis	10.3390/metabo10090347
**2-methyl-5-vinylpyrazine**	Aspergillus	fumigatus	10.1088/1752-7155/10/1/016002
**2-methylbenzoxazole**	Pseudomonas	aeruginosa	10.1016/j.jchromb.2012.05.038
**2-methyl-but-2-enal**	Staphylococcus	aureus	10.1186/1471-2180-12-113
**2-methyl-but-2-enal**	Staphylococcus	aureus	10.1186/1471-2180-12-113
**2-methylbutanenitrile**	Pseudomonas	aeruginosa	10.3390/metabo11110773
**2-methylbutanoic acid**	Staphylococcus	aureus	10.1016/j.jchromb.2009.05.028
**2-methylbutanoic acid**	Staphylococcus	epidermidis	10.3390/metabo10090347
**2-methylbutanoic acid**	Staphylococcus	epidermidis	10.3390/metabo10090347
**2-methylbutanoic acid**	Staphylococcus	epidermidis	10.3390/metabo10090347
**2-methylbutyl 2-methylbutyrate**	Pseudomonas	aeruginosa	10.1186/1471-2180-12-113
**2-methylbutyl acetate**	Staphylococcus	aureus	10.3390/metabo10090347
**2-methylbutyl acetate**	Staphylococcus	epidermidis	10.3390/metabo10090347
**2-methylbutyl isobutyrate**	Pseudomonas	aeruginosa	10.1186/1471-2180-12-113
**2-methylbutyl isovalerate**	Pseudomonas	aeruginosa	10.3389/fmicb.2021.693075
**2-methylbutyl isovalerate**	Pseudomonas	aeruginosa	10.3389/fmicb.2021.693075

**2-methylbutyl isovalerate**	Pseudomonas	aeruginosa	10.3389/fmicb.2021.693075
**2-methylbutyl isovalerate**	Pseudomonas	aeruginosa	10.3389/fmicb.2021.693075
**2-methylbutyl isovalerate**	Pseudomonas	aeruginosa	10.3389/fmicb.2021.693075
**2-methylbutyraldehyde**	Pseudomonas	aeruginosa	10.1088/1752-7155/10/1/016002
**2-methylbutyraldehyde**	Staphylococcus	aureus	10.1016/j.mimet.2010.12.001
**2-methylbutyraldehyde**	Staphylococcus	aureus	10.1016/j.mimet.2010.12.001
**2-methylbutyraldehyde**	Staphylococcus	aureus	10.1109/JSEN.2009.2035671
**2-methylbutyraldehyde**	Proteus	mirabilis	10.1016/j.mimet.2010.12.001
**2-methylbutyraldehyde**	Proteus	mirabilis	10.1016/j.mimet.2010.12.001
**2-methylbutyraldehyde**	Klebsiella	pneumoniae	10.1088/1752-7155/10/2/027101
**2-methylbutyraldehyde**	Streptococcus	pneumoniae	10.1016/j.jchromb.2018.08.032
**2-methylfuran**	Klebsiella	pneumoniae	10.1111/jam.13372
**2-methylnaphthalene**	Enterobacter	cloacae	10.1007/s00253-012-3924-4
**2-methylnaphthalene**	Escherichia	coli	10.1007/s00253-012-3924-4
**2-methylpyrazine**	Pseudomonas	aeruginosa	10.1016/j.jchromb.2012.05.038
**2-methylpyrazine**	Pseudomonas	aeruginosa	10.1038/s41598-020-74909-w
**2-methylpyrazine**	Pseudomonas	aeruginosa	10.1038/s41598-020-74909-w
**2-methylpyrazine**	Staphylococcus	aureus	10.1038/s41598-020-74909-w
**2-methylpyrazine**	Staphylococcus	aureus	10.1038/s41598-020-74909-w
**2-methylpyrazine**	Staphylococcus	aureus	10.3390/metabo10090347
**2-methylpyrazine**	Staphylococcus	aureus	10.3390/metabo10090347
**2-methylpyrazine**	Staphylococcus	aureus	10.3390/metabo10090347
**2-methylpyrazine**	Staphylococcus	aureus	10.3390/metabo10090347
**2-methylpyrazine**	Escherichia	coli	10.1007/s00253-012-3924-4
**2-methylpyrazine**	Escherichia	coli	10.1038/s41598-020-74909-w
**2-methylpyrazine**	Escherichia	coli	10.1038/s41598-020-74909-w
**2-methylpyrazine**	Escherichia	coli	10.3390/antibiotics9110797
**2-methylpyrazine**	Escherichia	coli	10.3390/antibiotics9110797
**2-methylpyrazine**	Staphylococcus	epidermidis	10.1038/s41598-020-74909-w
**2-methylpyrazine**	Staphylococcus	epidermidis	10.1038/s41598-020-74909-w
**2-methylpyrazine**	Staphylococcus	epidermidis	10.3390/metabo10090347
**2-methylpyrazine**	Staphylococcus	epidermidis	10.3390/metabo10090347
**2-methylpyrazine**	Staphylococcus	epidermidis	10.3390/metabo10090347
**2-methylpyrazine**	Aspergillus	fumigatus	10.1039/c8an00841h
**2-methylpyrazine**	Aspergillus	fumigatus	10.1039/c8an00841h
**2-methylthiazole**	Escherichia	coli	10.1111/jam.15716
**2-methylthiolan-3-one**	Mycobacterium	bovis	10.1088/1752-7163/aa6e06
**2-methylthiolan-3-one**	Klebsiella	pneumoniae	10.1088/1752-7155/10/2/027101
**2-methylundecane-2-thiol**	Staphylococcus	aureus	10.3390/metabo10090347
**2-methylundecane-2-thiol**	Staphylococcus	epidermidis	10.3390/metabo10090347
**2-methylundecane-2-thiol**	Staphylococcus	epidermidis	10.3390/metabo10090347
**2-nonanol**	Pseudomonas	aeruginosa	10.1038/s41598-020-74909-w
**2-nonanol**	Pseudomonas	aeruginosa	10.1038/s41598-020-74909-w

**2-nonanol**	Pseudomonas	aeruginosa	10.3389/fmicb.2021.693075
**2-nonanol**	Pseudomonas	aeruginosa	10.3389/fmicb.2021.693075
**2-nonanol**	Pseudomonas	aeruginosa	10.3389/fmicb.2021.693075
**2-nonanol**	Pseudomonas	aeruginosa	10.3389/fmicb.2021.693075
**2-nonanol**	Pseudomonas	aeruginosa	10.3389/fmicb.2021.693075
**2-nonanol**	Pseudomonas	aeruginosa	10.3389/fmicb.2021.693075
**2-nonanol**	Escherichia	coli	10.1038/s41598-020-74909-w
**2-nonanone**	Pseudomonas	aeruginosa	10.1007/s00253-012-3924-4
**2-nonanone**	Pseudomonas	aeruginosa	10.1016/j.jchromb.2012.05.038
**2-nonanone**	Pseudomonas	aeruginosa	10.1016/S0378-4347(00)80760-4
**2-nonanone**	Pseudomonas	aeruginosa	10.1016/S0378-4347(00)80760-4
**2-nonanone**	Pseudomonas	aeruginosa	10.1038/s41598-020-74909-w
**2-nonanone**	Pseudomonas	aeruginosa	10.1088/1752-7155/10/4/047102
**2-nonanone**	Pseudomonas	aeruginosa	10.1088/1752-7155/8/2/027106
**2-nonanone**	Pseudomonas	aeruginosa	10.1088/1752-7155/8/2/027106
**2-nonanone**	Pseudomonas	aeruginosa	10.1128/JCM.12.4.521-526.1980
**2-nonanone**	Pseudomonas	aeruginosa	10.1128/JCM.12.4.521-526.1980
**2-nonanone**	Pseudomonas	aeruginosa	10.1128/JCM.12.4.521-526.1980
**2-nonanone**	Pseudomonas	aeruginosa	10.1128/JCM.12.4.521-526.1980
**2-nonanone**	Pseudomonas	aeruginosa	10.1128/JCM.12.4.521-526.1980
**2-nonanone**	Pseudomonas	aeruginosa	10.1128/JCM.12.4.521-526.1980
**2-nonanone**	Pseudomonas	aeruginosa	10.1128/JCM.12.4.521-526.1980
**2-nonanone**	Pseudomonas	aeruginosa	10.1128/JCM.12.4.521-526.1980
**2-nonanone**	Pseudomonas	aeruginosa	10.1128/JCM.12.4.521-526.1980
**2-nonanone**	Pseudomonas	aeruginosa	10.1128/JCM.12.4.521-526.1980
**2-nonanone**	Pseudomonas	aeruginosa	10.1128/JCM.12.4.521-526.1980
**2-nonanone**	Pseudomonas	aeruginosa	10.1128/mSphere.00843-20
**2-nonanone**	Pseudomonas	aeruginosa	10.1186/1471-2180-12-113
**2-nonanone**	Pseudomonas	aeruginosa	10.3389/fmicb.2021.693075
**2-nonanone**	Pseudomonas	aeruginosa	10.3389/fmicb.2021.693075
**2-nonanone**	Pseudomonas	aeruginosa	10.3389/fmicb.2021.693075
**2-nonanone**	Pseudomonas	aeruginosa	10.3389/fmicb.2021.693075
**2-nonanone**	Pseudomonas	aeruginosa	10.3389/fmicb.2021.693075
**2-nonanone**	Pseudomonas	aeruginosa	10.3389/fmicb.2021.693075
**2-nonanone**	Staphylococcus	aureus	10.1002/jobm.201600505
**2-nonanone**	Staphylococcus	aureus	10.1016/S0378-4347(00)80760-4
**2-nonanone**	Staphylococcus	aureus	10.1128/JCM.00392-10
**2-nonanone**	Staphylococcus	aureus	10.3390/metabo10090347
**2-nonanone**	Staphylococcus	aureus	10.3390/metabo10090347
**2-nonanone**	Staphylococcus	aureus	10.3390/metabo10090347
**2-nonanone**	Staphylococcus	aureus	10.3390/metabo10090347
**2-nonanone**	Enterobacter	cloacae	10.1007/s00253-012-3924-4
**2-nonanone**	Escherichia	coli	10.1002/jobm.201600505

**2-nonanone**	Escherichia	coli	10.1002/jssc.201800684
**2-nonanone**	Escherichia	coli	10.1007/s00216-009-2758-0
**2-nonanone**	Escherichia	coli	10.1007/s00253-012-3924-4
**2-nonanone**	Escherichia	coli	10.1038/s41598-020-74909-w
**2-nonanone**	Escherichia	coli	10.1111/jam.15716
**2-nonanone**	Escherichia	coli	10.1111/jam.15716
**2-nonanone**	Escherichia	coli	10.3389/fmicb.2021.693075
**2-nonanone**	Escherichia	coli	10.3389/fmicb.2021.693075
**2-nonanone**	Escherichia	coli	10.3389/fmicb.2021.693075
**2-nonanone**	Escherichia	coli	10.3389/fmicb.2021.693075
**2-nonanone**	Escherichia	coli	10.3389/fmicb.2021.693075
**2-nonanone**	Staphylococcus	epidermidis	10.3390/metabo10090347
**2-nonanone**	Staphylococcus	epidermidis	10.3390/metabo10090347
**2-nonanone**	Aspergillus	fumigatus	10.1088/1752-7155/10/1/016002
**2-nonanone**	Serratia	liquefaciens	10.1016/S0045-6535(97)00209-9
**2-nonanone**	Serratia	marcescens	10.1007/s00253-012-3924-4
**2-nonanone**	Proteus	mirabilis	10.1007/s00253-012-3924-4
**2-nonanone**	Klebsiella	pneumoniae	10.1007/s00253-012-3924-4
**2-nonanone**	Klebsiella	pneumoniae	10.1016/S0378-4347(00)80760-4
**2-nonanone**	Klebsiella	pneumoniae	10.1016/S0378-4347(00)80760-4
**2-nonanone**	Streptococcus	pneumoniae	10.1099/mic.0.062687-0
**2-octanone**	Staphylococcus	aureus	10.3390/metabo10090347
**2-octanone**	Staphylococcus	aureus	10.3390/metabo10090347
**2-octanone**	Staphylococcus	aureus	10.3390/metabo10090347
**2-octanone**	Staphylococcus	aureus	10.3390/metabo10090347
**2-octanone**	Enterobacter	cloacae	10.1007/s00253-012-3924-4
**2-octanone**	Staphylococcus	epidermidis	10.3390/metabo10090347
**2-octanone**	Staphylococcus	epidermidis	10.3390/metabo10090347
**2-octanone**	Staphylococcus	epidermidis	10.3390/metabo10090347
**2-octanone**	Staphylococcus	epidermidis	10.3390/metabo10090347
**2-octanone**	Aspergillus	fumigatus	10.1088/1752-7155/6/1/016002
**2-octanone**	Aspergillus	fumigatus	10.1088/1752-7155/6/1/016002
**2-octanone**	Aspergillus	fumigatus	10.1088/1752-7155/6/1/016002
**2-octenal**	Staphylococcus	epidermidis	10.3390/metabo10090347
**2-pentanol**	Pseudomonas	aeruginosa	10.3389/fmicb.2021.693075
**2-pentanol**	Pseudomonas	aeruginosa	10.3389/fmicb.2021.693075
**2-pentanol**	Pseudomonas	aeruginosa	10.3389/fmicb.2021.693075
**2-pentanol**	Pseudomonas	aeruginosa	10.3389/fmicb.2021.693075
**2-pentanol**	Pseudomonas	aeruginosa	10.3389/fmicb.2021.693075
**2-pentanol**	Pseudomonas	aeruginosa	10.3389/fmicb.2021.693075
**2-pentanol**	Staphylococcus	aureus	10.3389/fmicb.2021.693075
**2-pentanol**	Staphylococcus	aureus	10.3389/fmicb.2021.693075
**2-pentanol**	Escherichia	coli	10.1038/s41598-020-74909-w

**2-pentanol**	Staphylococcus	epidermidis	10.1038/s41598-020-74909-w
**2-pentanol**	Klebsiella	pneumoniae	10.1111/jam.13372
**2-pentanone**	Pseudomonas	aeruginosa	10.1016/S0378-4347(00)80760-4
**2-pentanone**	Pseudomonas	aeruginosa	10.1016/S0378-4347(00)80760-4
**2-pentanone**	Pseudomonas	aeruginosa	10.1088/1752-7155/10/4/047102
**2-pentanone**	Pseudomonas	aeruginosa	10.1128/JCM.00392-10
**2-pentanone**	Pseudomonas	aeruginosa	10.1186/1471-2180-12-113
**2-pentanone**	Pseudomonas	aeruginosa	10.3389/fmicb.2021.693075
**2-pentanone**	Pseudomonas	aeruginosa	10.3389/fmicb.2021.693075
**2-pentanone**	Pseudomonas	aeruginosa	10.3389/fmicb.2021.693075
**2-pentanone**	Pseudomonas	aeruginosa	10.3389/fmicb.2021.693075
**2-pentanone**	Pseudomonas	aeruginosa	10.3389/fmicb.2021.693075
**2-pentanone**	Pseudomonas	aeruginosa	10.3389/fmicb.2021.693075
**2-pentanone**	Staphylococcus	aureus	10.1016/S0378-4347(00)80760-4
**2-pentanone**	Staphylococcus	aureus	10.1128/JCM.00392-10
**2-pentanone**	Staphylococcus	aureus	10.3389/fmicb.2021.693075
**2-pentanone**	Staphylococcus	aureus	10.3389/fmicb.2021.693075
**2-pentanone**	Staphylococcus	aureus	10.3389/fmicb.2021.693075
**2-pentanone**	Staphylococcus	aureus	10.3389/fmicb.2021.693075
**2-pentanone**	Staphylococcus	aureus	10.3389/fmicb.2021.693075
**2-pentanone**	Staphylococcus	aureus	10.3389/fmicb.2021.693075
**2-pentanone**	Staphylococcus	aureus	10.3390/metabo10090347
**2-pentanone**	Staphylococcus	aureus	10.3390/metabo10090347
**2-pentanone**	Mycobacterium	bovis	10.1371/journal.pone.0194348
**2-pentanone**	Escherichia	coli	10.3389/fmicb.2021.693075
**2-pentanone**	Escherichia	coli	10.3389/fmicb.2021.693075
**2-pentanone**	Escherichia	coli	10.3389/fmicb.2021.693075
**2-pentanone**	Escherichia	coli	10.3389/fmicb.2021.693075
**2-pentanone**	Escherichia	coli	10.3389/fmicb.2021.693075
**2-pentanone**	Escherichia	coli	10.3389/fmicb.2021.693075
**2-pentanone**	Escherichia	coli	10.3390/antibiotics9110797
**2-pentanone**	Haemophilus	influenzae	10.1099/mic.0.062687-0
**2-pentanone**	Serratia	liquefaciens	10.1016/S0045-6535(97)00209-9
**2-pentanone**	Klebsiella	pneumoniae	10.1016/S0378-4347(00)80760-4
**2-pentanone**	Klebsiella	pneumoniae	10.1016/S0378-4347(00)80760-4
**2-pentanone**	Klebsiella	pneumoniae	10.1088/1752-7155/10/2/027101
**2-pentanone**	Streptococcus	pneumoniae	10.1099/mic.0.062687-0
**2-pentylfuran**	Escherichia	coli	10.3390/antibiotics9110797
**2-phenyl-2-propanol**	Staphylococcus	aureus	10.3390/metabo10090347
**2-phenyl-2-propanol**	Staphylococcus	epidermidis	10.3390/metabo10090347
**2-phenylethanol**	Streptococcus	agalactiae	10.1007/s00253-012-3924-4
**2-phenylethanol**	Candida	albicans	10.1038/srep27441
**2-phenylethanol**	Candida	albicans	10.1111/j.1439-0507.2011.02037.[illegible]

**2-phenylethanol**	Candida	albicans	10.1128/EC.00252-07
**2-phenylethanol**	Candida	albicans	10.3390/metabo12050432
**2-phenylethanol**	Candida	albicans	10.3390/metabo12050432
**2-phenylethanol**	Staphylococcus	aureus	10.1016/j.jchromb.2009.05.028
**2-phenylethanol**	Mycobacterium	bovis	10.1111/j.1574-6968.2011.02493.[illegible]
**2-phenylethanol**	Moraxella	catarrhalis	10.1016/j.jchromb.2009.05.028
**2-phenylethanol**	Penicillium	chrysogenum	10.1038/srep27441
**2-phenylethanol**	Enterobacter	cloacae	10.1007/s11306-018-1357-5
**2-phenylethanol**	Escherichia	coli	10.1002/jssc.201800684
**2-phenylethanol**	Escherichia	coli	10.1038/s41598-020-74909-w
**2-phenylethanol**	Escherichia	coli	10.1038/s41598-020-74909-w
**2-phenylethanol**	Escherichia	coli	10.1111/jam.15716
**2-phenylethanol**	Escherichia	coli	10.1111/jam.15716
**2-phenylethanol**	Escherichia	coli	10.3389/fmicb.2021.693075
**2-phenylethanol**	Escherichia	coli	10.3389/fmicb.2021.693075
**2-phenylethanol**	Escherichia	coli	10.3389/fmicb.2021.693075
**2-phenylethanol**	Escherichia	coli	10.3389/fmicb.2021.693075
**2-phenylethanol**	Escherichia	coli	10.3389/fmicb.2021.693075
**2-phenylethanol**	Escherichia	coli	10.3389/fmicb.2021.693075
**2-phenylethanol**	Proteus	mirabilis	10.1007/s00253-012-3924-4
**2-phenylethanol**	Aspergillus	niger	10.1038/srep27441
**2-phenylethanol**	Klebsiella	pneumoniae	10.1111/jam.13372
**2-phenylethanol**	Streptococcus	pneumoniae	10.1016/j.jchromb.2009.05.028
**2-propanone, methylhydrazone**	Candida	albicans	10.1038/srep27441
**2-propanone, methylhydrazone**	Penicillium	chrysogenum	10.1038/srep27441
**2-propanone, methylhydrazone**	Aspergillus	niger	10.1038/srep27441
**2-propionyl-1-pyrroline**	Stenotrophomonas	maltophilia	10.3390/metabo11110773
**2-propionylthiazole**	Streptococcus	pneumoniae	10.3390/metabo11110773
**2-pyrrolidinone**	Candida	albicans	10.3390/metabo12050432
**2-tetradecanone**	Staphylococcus	aureus	10.3390/metabo10090347
**2-tetradecanone**	Staphylococcus	aureus	10.3390/metabo10090347
**2-tetradecanone**	Staphylococcus	aureus	10.3390/metabo10090347
**2-tetradecanone**	Staphylococcus	epidermidis	10.3390/metabo10090347
**2-tridecanol**	Escherichia	coli	10.1038/s41598-020-74909-w
**2-tridecanol**	Escherichia	coli	10.3389/fmicb.2021.693075
**2-tridecanol**	Escherichia	coli	10.3389/fmicb.2021.693075
**2-tridecanol**	Escherichia	coli	10.3389/fmicb.2021.693075
**2-tridecanol**	Escherichia	coli	10.3389/fmicb.2021.693075
**2-tridecanol**	Escherichia	coli	10.3389/fmicb.2021.693075
**2-tridecanol**	Escherichia	coli	10.3389/fmicb.2021.693075
**2-tridecanone**	Pseudomonas	aeruginosa	10.1016/j.jchromb.2012.05.038
**2-tridecanone**	Staphylococcus	aureus	10.3390/metabo10090347
**2-tridecanone**	Staphylococcus	aureus	10.3390/metabo10090347

**2-tridecanone**	Staphylococcus	aureus	10.3390/metabo10090347
**2-tridecanone**	Staphylococcus	aureus	10.3390/metabo10090347
**2-tridecanone**	Escherichia	coli	10.1002/jssc.201800684
**2-tridecanone**	Escherichia	coli	10.1038/s41598-020-74909-w
**2-tridecanone**	Escherichia	coli	10.1038/s41598-020-74909-w
**2-tridecanone**	Escherichia	coli	10.1111/jam.15716
**2-tridecanone**	Escherichia	coli	10.1111/jam.15716
**2-tridecanone**	Escherichia	coli	10.3389/fmicb.2021.693075
**2-tridecanone**	Escherichia	coli	10.3389/fmicb.2021.693075
**2-tridecanone**	Escherichia	coli	10.3389/fmicb.2021.693075
**2-tridecanone**	Escherichia	coli	10.3389/fmicb.2021.693075
**2-tridecanone**	Escherichia	coli	10.3389/fmicb.2021.693075
**2-tridecanone**	Escherichia	coli	10.3389/fmicb.2021.693075
**2-tridecanone**	Aspergillus	fumigatus	10.1088/1752-7155/10/1/016002
**2-undecanol**	Pseudomonas	aeruginosa	10.1038/s41598-020-74909-w
**2-undecanol**	Pseudomonas	aeruginosa	10.3389/fmicb.2021.693075
**2-undecanol**	Pseudomonas	aeruginosa	10.3389/fmicb.2021.693075
**2-undecanol**	Pseudomonas	aeruginosa	10.3389/fmicb.2021.693075
**2-undecanol**	Pseudomonas	aeruginosa	10.3389/fmicb.2021.693075
**2-undecanol**	Pseudomonas	aeruginosa	10.3389/fmicb.2021.693075
**2-undecanol**	Pseudomonas	aeruginosa	10.3389/fmicb.2021.693075
**2-undecanol**	Escherichia	coli	10.1038/s41598-020-74909-w
**2-undecanol**	Escherichia	coli	10.1038/s41598-020-74909-w
**2-undecanol**	Escherichia	coli	10.3389/fmicb.2021.693075
**2-undecanol**	Escherichia	coli	10.3389/fmicb.2021.693075
**2-undecanone**	Pseudomonas	aeruginosa	10.1016/j.jchromb.2012.05.038
**2-undecanone**	Pseudomonas	aeruginosa	10.1016/S0378-4347(00)80760-4
**2-undecanone**	Pseudomonas	aeruginosa	10.1016/S0378-4347(00)80760-4
**2-undecanone**	Pseudomonas	aeruginosa	10.1038/s41598-020-74909-w
**2-undecanone**	Pseudomonas	aeruginosa	10.1038/s41598-020-74909-w
**2-undecanone**	Pseudomonas	aeruginosa	10.1128/JCM.12.4.521-526.1980
**2-undecanone**	Pseudomonas	aeruginosa	10.1128/JCM.12.4.521-526.1980
**2-undecanone**	Pseudomonas	aeruginosa	10.1128/JCM.12.4.521-526.1980
**2-undecanone**	Pseudomonas	aeruginosa	10.1128/JCM.12.4.521-526.1980
**2-undecanone**	Pseudomonas	aeruginosa	10.1128/JCM.12.4.521-526.1980
**2-undecanone**	Pseudomonas	aeruginosa	10.1128/JCM.12.4.521-526.1980
**2-undecanone**	Pseudomonas	aeruginosa	10.1128/JCM.12.4.521-526.1980
**2-undecanone**	Pseudomonas	aeruginosa	10.1128/JCM.12.4.521-526.1980
**2-undecanone**	Pseudomonas	aeruginosa	10.1128/JCM.12.4.521-526.1980
**2-undecanone**	Pseudomonas	aeruginosa	10.1128/JCM.12.4.521-526.1980
**2-undecanone**	Pseudomonas	aeruginosa	10.1128/JCM.12.4.521-526.1980
**2-undecanone**	Pseudomonas	aeruginosa	10.3389/fmicb.2021.693075
**2-undecanone**	Pseudomonas	aeruginosa	10.3389/fmicb.2021.693075

**2-undecanone**	Pseudomonas	aeruginosa	10.3389/fmicb.2021.693075
**2-undecanone**	Pseudomonas	aeruginosa	10.3389/fmicb.2021.693075
**2-undecanone**	Pseudomonas	aeruginosa	10.3389/fmicb.2021.693075
**2-undecanone**	Pseudomonas	aeruginosa	10.3389/fmicb.2021.693075
**2-undecanone**	Streptococcus	agalactiae	10.1007/s00253-012-3924-4
**2-undecanone**	Staphylococcus	aureus	10.1002/jobm.201600505
**2-undecanone**	Staphylococcus	aureus	10.3390/metabo10090347
**2-undecanone**	Staphylococcus	aureus	10.3390/metabo10090347
**2-undecanone**	Staphylococcus	aureus	10.3390/metabo10090347
**2-undecanone**	Escherichia	coli	10.1002/jobm.201600505
**2-undecanone**	Escherichia	coli	10.1007/s00253-012-3924-4
**2-undecanone**	Escherichia	coli	10.1038/s41598-020-74909-w
**2-undecanone**	Escherichia	coli	10.1038/s41598-020-74909-w
**2-undecanone**	Escherichia	coli	10.3389/fmicb.2021.693075
**2-undecanone**	Escherichia	coli	10.3389/fmicb.2021.693075
**2-undecanone**	Escherichia	coli	10.3389/fmicb.2021.693075
**2-undecanone**	Escherichia	coli	10.3389/fmicb.2021.693075
**2-undecanone**	Escherichia	coli	10.3389/fmicb.2021.693075
**2-undecanone**	Escherichia	coli	10.3389/fmicb.2021.693075
**2-undecanone**	Aspergillus	fumigatus	10.1088/1752-7155/10/1/016002
**2-undecanone**	Stenotrophomonas	maltophilia	10.1128/JCM.12.4.521-526.1980
**2-undecanone**	Serratia	marcescens	10.1007/s00253-012-3924-4
**2-undecanone**	Proteus	mirabilis	10.1007/s00253-012-3924-4
**2-undecanone**	Klebsiella	pneumoniae	10.1016/S0378-4347(00)80760-4
**2-undecanone**	Klebsiella	pneumoniae	10.1016/S0378-4347(00)80760-4
**2-undecanone**	Pseudomonas	putida	10.1128/JCM.12.4.521-526.1980
**3-(ethylsulfanyl)propanal**	Pseudomonas	aeruginosa	10.1186/1471-2180-12-113
**3,3-dimethyl-1-heptene**	Escherichia	coli	10.3390/antibiotics9110797
**3,3-dimethyloctane**	Staphylococcus	aureus	10.3390/metabo10090347
**3,3-dimethyloctane**	Staphylococcus	aureus	10.3390/metabo10090347
**3,3-dimethyloctane**	Staphylococcus	epidermidis	10.3390/metabo10090347
**3,3-dimethyloctane**	Staphylococcus	epidermidis	10.3390/metabo10090347
**3,3-dimethyloctane**	Staphylococcus	epidermidis	10.3390/metabo10090347
**3,3-dimethyloctane**	Staphylococcus	epidermidis	10.3390/metabo10090347
**3,5-dimethylamphetamine**	Mycobacterium	tuberculosis	10.1016/j.tube.2006.03.004
**3,5-dimethylamphetamine**	Mycobacterium	tuberculosis	10.1016/j.tube.2006.03.004
**3,7-dimethyl-1-octene**	Staphylococcus	aureus	10.3390/metabo10090347
**3,7-dimethyl-1-octene**	Staphylococcus	aureus	10.3390/metabo10090347
**3,7-dimethyl-1-octene**	Staphylococcus	aureus	10.3390/metabo10090347
**3,7-dimethyl-1-octene**	Staphylococcus	aureus	10.3390/metabo10090347
**3,7-dimethyl-1-octene**	Staphylococcus	epidermidis	10.3390/metabo10090347
**3,7-dimethyl-1-octene**	Staphylococcus	epidermidis	10.3390/metabo10090347
**3,7-dimethyl-1-octene**	Staphylococcus	epidermidis	10.3390/metabo10090347

**3,7-dimethyl-1-octene**	Staphylococcus	epidermidis	10.3390/metabo10090347
**3-acetylpyrrole**	Escherichia	coli	10.1111/jam.15716
**3-acetylthiophene**	Streptococcus	pneumoniae	10.3390/metabo11110773
**3-butyl-2,5-dimethylpyrazine**	Staphylococcus	aureus	10.3390/metabo10090347
**3-butyl-2,5-dimethylpyrazine**	Staphylococcus	aureus	10.3390/metabo10090347
**3-butyl-2,5-dimethylpyrazine**	Staphylococcus	aureus	10.3390/metabo10090347
**3-butyl-2,5-dimethylpyrazine**	Escherichia	coli	10.1111/jam.15716
**3-decanone**	Pseudomonas	aeruginosa	10.1016/j.jchromb.2012.05.038
**3-decanone**	Pseudomonas	aeruginosa	10.1128/mSphere.00843-20
**3-ethenylcyclohexa-3,5-diene-1,2-diol**	Streptococcus	pneumoniae	10.3390/metabo11110773
**3-ethyl-2,5-dimethylpyrazine**	Pseudomonas	aeruginosa	10.1038/s41598-020-74909-w
**3-ethyl-2,5-dimethylpyrazine**	Staphylococcus	aureus	10.1038/s41598-020-74909-w
**3-ethyl-2,5-dimethylpyrazine**	Staphylococcus	aureus	10.1038/s41598-020-74909-w
**3-ethyl-2,5-dimethylpyrazine**	Staphylococcus	aureus	10.3390/metabo10090347
**3-ethyl-2,5-dimethylpyrazine**	Staphylococcus	aureus	10.3390/metabo10090347
**3-ethyl-2,5-dimethylpyrazine**	Escherichia	coli	10.1038/s41598-020-74909-w
**3-ethyl-2,5-dimethylpyrazine**	Escherichia	coli	10.1111/jam.15716
**3-ethyl-2,5-dimethylpyrazine**	Staphylococcus	epidermidis	10.3390/metabo10090347
**3-ethylidenecycloheptene**	Aspergillus	fumigatus	10.1128/EC.00074-14
**3-heptanone**	Pseudomonas	aeruginosa	10.1088/1752-7155/10/4/047102
**3-heptanone**	Candida	albicans	10.1038/srep27441
**3-heptanone**	Penicillium	chrysogenum	10.1038/srep27441
**3-heptanone**	Aspergillus	niger	10.1038/srep27441
**3-heptanone**	Klebsiella	pneumoniae	10.1111/jam.13372
**3-heptanone**	Mycobacterium	tuberculosis	10.1016/j.tube.2006.03.004
**3-heptanone**	Mycobacterium	tuberculosis	10.1016/j.tube.2006.03.004
**3-Hydroxy-2,4,4-trimethylpentyl 2-methyl-propan**	Candida	albicans	10.1038/srep27441
**3-Hydroxy-2,4,4-trimethylpentyl 2-methyl-propan**	Penicillium	chrysogenum	10.1038/srep27441
**3-Hydroxy-2,4,4-trimethylpentyl 2-methyl-propan**	Aspergillus	niger	10.1038/srep27441
**3-methyl-1-butene**	Haemophilus	influenzae	10.1099/mic.0.062687-0
**3-methyl-1-butene**	Klebsiella	pneumoniae	10.1111/jam.13372
**3-methyl-1h-indene**	Acinetobacter	baumannii	10.1088/1752-7155/10/2/027102
**3-methyl-1h-pyrrole**	Pseudomonas	aeruginosa	10.1088/1752-7155/10/1/016002
**3-methyl-1h-pyrrole**	Pseudomonas	aeruginosa	10.1186/1471-2180-12-113
**3-methyl-2-butanone**	Pseudomonas	aeruginosa	10.1088/1752-7155/10/4/047102
**3-methyl-2-butanone**	Staphylococcus	aureus	10.1038/s41598-020-74909-w
**3-methyl-2-butanone**	Staphylococcus	aureus	10.1038/s41598-020-74909-w
**3-methyl-2-butanone**	Staphylococcus	aureus	10.3390/metabo10090347
**3-methyl-2-butanone**	Staphylococcus	aureus	10.3390/metabo10090347
**3-methyl-2-butanone**	Staphylococcus	aureus	10.3390/metabo10090347
**3-methyl-2-butanone**	Escherichia	coli	10.1038/s41598-020-74909-w
**3-methyl-2-butanone**	Staphylococcus	epidermidis	10.1038/s41598-020-74909-w
**3-methyl-2-butanone**	Staphylococcus	epidermidis	10.3390/metabo10090347

**3-methyl-2-buten-1-ol**	Streptococcus	agalactiae	10.1007/s00253-012-3924-4
**3-methyl-2-buten-1-ol**	Aspergillus	fumigatus	10.1128/EC.00074-14
**3-methyl-2-butenal**	Streptococcus	agalactiae	10.1007/s00253-012-3924-4
**3-methyl-2-butenal**	Staphylococcus	aureus	10.1186/1471-2180-12-113
**3-methyl-2-butenal**	Escherichia	coli	10.3390/antibiotics9110797
**3-methyl-2-butenal**	Aspergillus	fumigatus	10.1128/EC.00074-14
**3-methyl-2-butenal**	Streptococcus	pneumoniae	10.1099/mic.0.062687-0
**3-methyl-2-pentanone**	Pseudomonas	aeruginosa	10.1016/j.jchromb.2012.05.038
**3-methyl-2-pentanone**	Pseudomonas	aeruginosa	10.1088/1752-7155/10/4/047102
**3-methyl-3-buten-1-ol**	Pseudomonas	aeruginosa	10.1038/s41598-020-74909-w
**3-methyl-3-buten-1-ol**	Pseudomonas	aeruginosa	10.1128/mSphere.00843-20
**3-methyl-3-buten-1-ol**	Pseudomonas	aeruginosa	10.3389/fmicb.2021.693075
**3-methyl-3-buten-1-ol**	Pseudomonas	aeruginosa	10.3389/fmicb.2021.693075
**3-methyl-3-buten-1-ol**	Pseudomonas	aeruginosa	10.3389/fmicb.2021.693075
**3-methyl-3-buten-1-ol**	Pseudomonas	aeruginosa	10.3389/fmicb.2021.693075
**3-methyl-3-buten-1-ol**	Pseudomonas	aeruginosa	10.3389/fmicb.2021.693075
**3-methyl-3-buten-1-ol**	Pseudomonas	aeruginosa	10.3389/fmicb.2021.693075
**3-methyl-3-buten-1-ol**	Candida	albicans	10.3390/metabo12050432
**3-methyl-3-buten-1-ol**	Staphylococcus	aureus	10.1038/s41598-020-74909-w
**3-methyl-3-buten-1-ol**	Staphylococcus	aureus	10.1038/s41598-020-74909-w
**3-methyl-3-buten-1-ol**	Staphylococcus	aureus	10.3389/fmicb.2021.693075
**3-methyl-3-buten-1-ol**	Staphylococcus	aureus	10.3389/fmicb.2021.693075
**3-methyl-3-buten-1-ol**	Staphylococcus	aureus	10.3389/fmicb.2021.693075
**3-methyl-3-buten-1-ol**	Staphylococcus	aureus	10.3389/fmicb.2021.693075
**3-methyl-3-buten-1-ol**	Escherichia	coli	10.1038/s41598-020-74909-w
**3-methyl-3-buten-1-ol**	Escherichia	coli	10.3389/fmicb.2021.693075
**3-methyl-3-buten-1-ol**	Escherichia	coli	10.3389/fmicb.2021.693075
**3-methyl-3-buten-1-ol**	Escherichia	coli	10.3389/fmicb.2021.693075
**3-methyl-3-buten-1-ol**	Escherichia	coli	10.3389/fmicb.2021.693075
**3-methyl-3-buten-1-ol**	Escherichia	coli	10.3389/fmicb.2021.693075
**3-methyl-3-buten-1-ol**	Escherichia	coli	10.3389/fmicb.2021.693075
**3-methyl-3-buten-1-ol**	Staphylococcus	epidermidis	10.1038/s41598-020-74909-w
**3-methyl-3-buten-1-ol**	Staphylococcus	epidermidis	10.1038/s41598-020-74909-w
**3-methyl-3-buten-1-ol**	Staphylococcus	epidermidis	10.3390/metabo10090347
**3-methyl-3-buten-1-ol**	Aspergillus	fumigatus	10.1128/EC.00074-14
**3-methyl-3-buten-1-ol**	Klebsiella	pneumoniae	10.1111/jam.13372
**3-methyl-3-buten-2-one**	Pseudomonas	aeruginosa	10.1088/1752-7155/10/4/047102
**3-methyl-3-butenyl acetate**	Staphylococcus	aureus	10.3390/metabo10090347
**3-methyl-3-butenyl acetate**	Staphylococcus	aureus	10.3390/metabo10090347
**3-methyl-3-penten-2-one**	Pseudomonas	aeruginosa	10.1088/1752-7155/10/4/047102
**3-methylbutanal**	Pseudomonas	aeruginosa	10.1007/s00253-012-3924-4
**3-methylbutanal**	Pseudomonas	aeruginosa	10.1016/S0378-4347(00)80760-4
**3-methylbutanal**	Pseudomonas	aeruginosa	10.1016/S0378-4347(00)80760-4

**3-methylbutanal**	Pseudomonas	aeruginosa	10.1128/mSphere.00843-20
**3-methylbutanal**	Streptococcus	agalactiae	10.1007/s00253-012-3924-4
**3-methylbutanal**	Candida	albicans	10.1038/srep27441
**3-methylbutanal**	Staphylococcus	aureus	10.1002/jobm.201600505
**3-methylbutanal**	Staphylococcus	aureus	10.1007/s00253-012-3924-4
**3-methylbutanal**	Staphylococcus	aureus	10.1016/S0378-4347(00)80760-4
**3-methylbutanal**	Staphylococcus	aureus	10.1038/s41598-020-74909-w
**3-methylbutanal**	Staphylococcus	aureus	10.1038/s41598-020-74909-w
**3-methylbutanal**	Staphylococcus	aureus	10.1088/1752-7163/aa8efc
**3-methylbutanal**	Staphylococcus	aureus	10.1093/chromsci/bmt042
**3-methylbutanal**	Staphylococcus	aureus	10.1093/chromsci/bmt042
**3-methylbutanal**	Staphylococcus	aureus	10.1109/JSEN.2009.2035671
**3-methylbutanal**	Staphylococcus	aureus	10.1109/JSEN.2009.2035671
**3-methylbutanal**	Staphylococcus	aureus	10.1186/1471-2180-12-113
**3-methylbutanal**	Penicillium	chrysogenum	10.1038/srep27441
**3-methylbutanal**	Escherichia	coli	10.1007/s00253-012-3924-4
**3-methylbutanal**	Escherichia	coli	10.3390/antibiotics9110797
**3-methylbutanal**	Escherichia	coli	10.3390/antibiotics9110797
**3-methylbutanal**	Escherichia	coli	10.3390/antibiotics9110797
**3-methylbutanal**	Staphylococcus	epidermidis	10.1007/s00253-012-3924-4
**3-methylbutanal**	Staphylococcus	epidermidis	10.1038/s41598-020-74909-w
**3-methylbutanal**	Staphylococcus	epidermidis	10.1038/s41598-020-74909-w
**3-methylbutanal**	Staphylococcus	epidermidis	10.3390/metabo10090347
**3-methylbutanal**	Aspergillus	fumigatus	10.1039/c8an00841h
**3-methylbutanal**	Serratia	marcescens	10.1007/s00253-012-3924-4
**3-methylbutanal**	Aspergillus	niger	10.1038/srep27441
**3-methylbutanal**	Klebsiella	pneumoniae	10.1007/s00253-012-3924-4
**3-methylbutanal**	Klebsiella	pneumoniae	10.1016/S0378-4347(00)80760-4
**3-methylbutanal**	Klebsiella	pneumoniae	10.1016/S0378-4347(00)80760-4
**3-methylbutanal**	Klebsiella	pneumoniae	10.1088/1752-7163/aa8efc
**3-methylbutanal**	Streptococcus	pneumoniae	10.1007/s00253-012-3924-4
**3-methylbutanal**	Mycobacterium	tuberculosis	10.1016/j.tube.2006.03.004
**3-methylbutanal**	Mycobacterium	tuberculosis	10.1016/j.tube.2006.03.004
**3-methylcyclohexene**	Klebsiella	pneumoniae	10.1088/1752-7155/8/2/027106
**3-methylheptan-4-one**	Aspergillus	fumigatus	10.1088/1752-7155/6/1/016002
**3-methylheptan-4-one**	Aspergillus	fumigatus	10.1088/1752-7155/6/1/016002
**3-methylheptane**	Escherichia	coli	10.1088/1752-7155/8/2/027106
**3-methylheptane**	Escherichia	coli	10.1088/1752-7155/8/2/027106
**3-methylheptyl acetate**	Klebsiella	pneumoniae	10.1088/1752-7155/10/2/027101
**3-methylhexane**	Klebsiella	pneumoniae	10.1111/jam.13372
**3-methylindole**	Escherichia	coli	10.1002/jssc.201800684
**3-methylindole**	Escherichia	coli	10.1007/s00253-012-3924-4
**3-nonen-2-one**	Candida	albicans	10.1038/srep27441

**3-nonen-2-one**	Penicillium	chrysogenum	10.1038/srep27441
**3-nonen-2-one**	Aspergillus	niger	10.1038/srep27441
**3-octanol**	Candida	albicans	10.1038/srep27441
**3-octanol**	Mycobacterium	bovis	10.1371/journal.pone.0194348
**3-octanol**	Penicillium	chrysogenum	10.1038/srep27441
**3-octanol**	Aspergillus	niger	10.1038/srep27441
**3-octanone**	Pseudomonas	aeruginosa	10.1088/1752-7155/10/4/047102
**3-octanone**	Pseudomonas	aeruginosa	10.1128/mSphere.00843-20
**3-octanone**	Pseudomonas	aeruginosa	10.1186/1471-2180-12-113
**3-octanone**	Staphylococcus	aureus	10.3390/metabo10090347
**3-octanone**	Staphylococcus	aureus	10.3390/metabo10090347
**3-octanone**	Mycobacterium	bovis	10.1371/journal.pone.0194348
**3-octanone**	Staphylococcus	epidermidis	10.3390/metabo10090347
**3-octanone**	Staphylococcus	epidermidis	10.3390/metabo10090347
**3-octanone**	Staphylococcus	epidermidis	10.3390/metabo10090347
**3-octanone**	Staphylococcus	epidermidis	10.3390/metabo10090347
**3-octanone**	Aspergillus	fumigatus	10.1088/1752-7155/6/1/016002
**3-octanone**	Aspergillus	fumigatus	10.1088/1752-7155/6/1/016002
**3-octanone**	Aspergillus	fumigatus	10.1088/1752-7155/6/1/016002
**3-octanone**	Aspergillus	fumigatus	10.1111/j.1439-0507.2011.02037.
**3-octanone**	Aspergillus	fumigatus	10.1128/EC.00074-14
**3-pentanone**	Mycobacterium	bovis	10.1371/journal.pone.0194348
**3-penten-2-one**	Pseudomonas	aeruginosa	10.1128/mSphere.00843-20
**3-penten-2-one**	Mycobacterium	tuberculosis	10.5588/ijtld.11.0576
**3-phenylfuran**	Escherichia	coli	10.1111/jam.15716
**3-phenylundecane**	Escherichia	coli	10.1111/jam.15716
**4,6-dimethylheptan-2-one**	Pseudomonas	aeruginosa	10.1128/mSphere.00843-20
**4,6-dimethylheptan-2-one**	Pseudomonas	aeruginosa	10.3389/fmicb.2021.693075
**4,6-dimethylheptan-2-one**	Pseudomonas	aeruginosa	10.3389/fmicb.2021.693075
**4,6-dimethylheptan-2-one**	Pseudomonas	aeruginosa	10.3389/fmicb.2021.693075
**4,6-dimethylheptan-2-one**	Pseudomonas	aeruginosa	10.3389/fmicb.2021.693075
**4,6-dimethylheptan-2-one**	Pseudomonas	aeruginosa	10.3389/fmicb.2021.693075
**4,6-dimethylheptan-2-one**	Staphylococcus	aureus	10.3390/metabo10090347
**4,6-dimethylheptan-2-one**	Escherichia	coli	10.3389/fmicb.2021.693075
**4,6-dimethylheptan-2-one**	Escherichia	coli	10.3389/fmicb.2021.693075
**4,6-dimethylheptan-2-one**	Escherichia	coli	10.3389/fmicb.2021.693075
**4,6-dimethylheptan-2-one**	Escherichia	coli	10.3389/fmicb.2021.693075
**4-butylaniline**	Proteus	mirabilis	10.1007/s00253-012-3924-4
**4-chloroindole**	Escherichia	coli	10.1007/s00253-012-3924-4
**4-ethyl-2,2,6,6-tetramethylheptane**	Mycobacterium	tuberculosis	10.1088/1752-7163/aacd18
**4'-ethylacetophenone**	Pseudomonas	aeruginosa	10.1128/mSphere.00843-20
**4-ethyl-o-xylene**	Pseudomonas	aeruginosa	10.1128/mSphere.00843-20
**4-ethyltoluene**	Candida	albicans	10.1038/srep27441

**4-ethyltoluene**	Penicillium	chrysogenum	10.1038/srep27441
**4-ethyltoluene**	Aspergillus	niger	10.1038/srep27441
**4-heptanone**	Pseudomonas	aeruginosa	10.1186/1471-2180-12-113
**4-heptanone**	Aspergillus	fumigatus	10.1088/1752-7155/6/1/016002
**4-heptanone**	Aspergillus	fumigatus	10.1088/1752-7155/6/1/016002
**4-heptanone**	Haemophilus	influenzae	10.1099/mic.0.062687-0
**4-methoxybenzhydrazide**	Mycobacterium	bovis	10.1088/1752-7163/aa6e06
**4-methoxybenzhydrazide**	Mycobacterium	intracellulare	10.1088/1752-7163/aa6e06
**4-methyl-2-heptanol**	Pseudomonas	aeruginosa	10.3389/fmicb.2021.693075
**4-methyl-2-heptanol**	Pseudomonas	aeruginosa	10.3389/fmicb.2021.693075
**4-methyl-2-heptanone**	Pseudomonas	aeruginosa	10.1038/s41598-020-74909-w
**4-methyl-2-heptanone**	Pseudomonas	aeruginosa	10.1038/s41598-020-74909-w
**4-methyl-2-heptanone**	Staphylococcus	aureus	10.1038/s41598-020-74909-w
**4-methyl-2-heptanone**	Staphylococcus	aureus	10.1038/s41598-020-74909-w
**4-methyl-2-heptanone**	Staphylococcus	aureus	10.3390/metabo10090347
**4-methyl-2-heptanone**	Staphylococcus	aureus	10.3390/metabo10090347
**4-methyl-2-heptanone**	Staphylococcus	aureus	10.3390/metabo10090347
**4-methyl-2-heptanone**	Staphylococcus	aureus	10.3390/metabo10090347
**4-methyl-2-heptanone**	Escherichia	coli	10.1038/s41598-020-74909-w
**4-methyl-2-heptanone**	Staphylococcus	epidermidis	10.1038/s41598-020-74909-w
**4-methyl-2-heptanone**	Staphylococcus	epidermidis	10.3390/metabo10090347
**4-methyl-2-heptanone**	Staphylococcus	epidermidis	10.3390/metabo10090347
**4-methyl-2-pentanone**	Pseudomonas	aeruginosa	10.1038/s41598-020-74909-w
**4-methyl-2-pentanone**	Pseudomonas	aeruginosa	10.1088/1752-7155/10/4/047102
**4-methyl-2-pentanone**	Pseudomonas	aeruginosa	10.1186/1471-2180-12-113
**4-methyl-2-pentanone**	Pseudomonas	aeruginosa	10.3389/fmicb.2021.693075
**4-methyl-2-pentanone**	Pseudomonas	aeruginosa	10.3389/fmicb.2021.693075
**4-methyl-2-pentanone**	Staphylococcus	aureus	10.1038/s41598-020-74909-w
**4-methyl-2-pentanone**	Staphylococcus	aureus	10.3389/fmicb.2021.693075
**4-methyl-2-pentanone**	Staphylococcus	aureus	10.3389/fmicb.2021.693075
**4-methyl-2-pentanone**	Staphylococcus	aureus	10.3389/fmicb.2021.693075
**4-methyl-2-pentanone**	Staphylococcus	aureus	10.3389/fmicb.2021.693075
**4-methyl-2-pentanone**	Staphylococcus	aureus	10.3390/metabo10090347
**4-methyl-2-pentanone**	Staphylococcus	aureus	10.3390/metabo10090347
**4-methyl-2-pentanone**	Escherichia	coli	10.1038/s41598-020-74909-w
**4-methyl-2-pentanone**	Staphylococcus	epidermidis	10.1038/s41598-020-74909-w
**4-methyl-2-pentanone**	Staphylococcus	epidermidis	10.3390/metabo10090347
**4-methyl-2-pentanone**	Staphylococcus	epidermidis	10.3390/metabo10090347
**4-methylanisole**	Serratia	marcescens	10.1007/s00253-012-3924-4
**4-methylcyclohexene**	Klebsiella	pneumoniae	10.1088/1752-7155/8/2/027106
**4-methylheptane**	Staphylococcus	epidermidis	10.3390/metabo10090347
**4-methylhexanoic acid**	Staphylococcus	aureus	10.1016/j.jchromb.2009.05.028
**4-methylquinazoline**	Pseudomonas	aeruginosa	10.1016/j.jchromb.2012.05.038

**4-nonanone**	Pseudomonas	aeruginosa	10.1128/mSphere.00843-20
**4-penten-2-one, 4-methyl-**	Pseudomonas	aeruginosa	10.1016/j.jchromb.2012.05.038
**4-pentenyl acetate**	Klebsiella	pneumoniae	10.1111/jam.13372
**4-pentylaniline**	Proteus	mirabilis	10.1007/s00253-012-3924-4
**4-tert-amylphenol**	Mycobacterium	tuberculosis	10.1088/1752-7163/aacd18
**4-undecanone**	Pseudomonas	aeruginosa	10.1128/mSphere.00843-20
**5-methyl-2-heptanone**	Staphylococcus	epidermidis	10.1038/s41598-020-74909-w
**5-methylheptan-3-one**	Pseudomonas	aeruginosa	10.1007/s00253-013-4762-8
**5-methylheptan-3-one**	Pseudomonas	aeruginosa	10.1007/s00253-013-4762-8
**5-methylheptan-3-one**	Escherichia	coli	10.1007/s00253-013-4762-8
**5-methylheptan-3-one**	Escherichia	coli	10.1007/s00253-013-4762-8
**6,10-dimethylundeca-5,9-dien-2-one**	Candida	albicans	10.1038/srep27441
**6,10-dimethylundeca-5,9-dien-2-one**	Penicillium	chrysogenum	10.1038/srep27441
**6,10-dimethylundeca-5,9-dien-2-one**	Aspergillus	niger	10.1038/srep27441
**6-methyl-1-heptanol**	Staphylococcus	aureus	10.3390/metabo10090347
**6-methyl-1-heptanol**	Staphylococcus	aureus	10.3390/metabo10090347
**6-methyl-5-hepten-2-one**	Candida	albicans	10.1038/srep27441
**6-methyl-5-hepten-2-one**	Staphylococcus	aureus	10.3390/metabo10090347
**6-methyl-5-hepten-2-one**	Penicillium	chrysogenum	10.1038/srep27441
**6-methyl-5-hepten-2-one**	Aspergillus	fumigatus	10.1128/EC.00074-14
**6-methyl-5-hepten-2-one**	Aspergillus	niger	10.1038/srep27441
**6-methylheptan-2-one**	Pseudomonas	aeruginosa	10.1128/mSphere.00843-20
**6-methylheptan-2-one**	Staphylococcus	aureus	10.3390/metabo10090347
**6-methylheptan-2-one**	Staphylococcus	aureus	10.3390/metabo10090347
**6-methylheptan-2-one**	Staphylococcus	epidermidis	10.3390/metabo10090347
**6-methylheptan-2-one**	Staphylococcus	epidermidis	10.3390/metabo10090347
**6-phenylundecane**	Escherichia	coli	10.1111/jam.15716
**6-phenylundecane**	Escherichia	coli	10.1111/jam.15716
**6-tridecyne**	Pseudomonas	aeruginosa	10.1088/1752-7155/10/1/016002
**8-nonen-2-one**	Aspergillus	fumigatus	10.1088/1752-7155/10/1/016002
**9-decen-1-ol**	Escherichia	coli	10.1093/chromsci/bmt042
**9-oxononanoic acid**	Streptococcus	pneumoniae	10.3390/metabo11110773
**acetaldehyde**	Pseudomonas	aeruginosa	10.1016/j.diagmicrobio.2006.01.0[illegible]
**acetaldehyde**	Pseudomonas	aeruginosa	10.1088/1752-7155/10/3/037102
**acetaldehyde**	Pseudomonas	aeruginosa	10.1088/1752-7155/10/3/037102
**acetaldehyde**	Pseudomonas	aeruginosa	10.1088/1752-7155/10/3/037102
**acetaldehyde**	Staphylococcus	aureus	10.1016/j.diagmicrobio.2006.01.0[illegible]
**acetaldehyde**	Staphylococcus	aureus	10.1016/j.mimet.2005.09.003
**acetaldehyde**	Staphylococcus	aureus	10.1016/j.mimet.2010.12.001
**acetaldehyde**	Staphylococcus	aureus	10.1088/1752-7155/10/3/037102
**acetaldehyde**	Staphylococcus	aureus	10.1088/1752-7155/10/3/037102
**acetaldehyde**	Staphylococcus	aureus	10.1088/1752-7155/10/3/037102
**acetaldehyde**	Staphylococcus	aureus	10.1109/JSEN.2009.2035671

**acetaldehyde**	Staphylococcus	aureus	10.1109/JSEN.2009.2035671
**acetaldehyde**	Staphylococcus	aureus	10.1186/1471-2180-12-113
**acetaldehyde**	Mycobacterium	bovis	10.1111/j.1574-6968.2011.02493.[illegible]
**acetaldehyde**	Burkholderia	cepacia	10.1088/1752-7155/10/3/037102
**acetaldehyde**	Burkholderia	cepacia	10.1088/1752-7155/10/3/037102
**acetaldehyde**	Burkholderia	cepacia	10.1088/1752-7155/10/3/037102
**acetaldehyde**	Escherichia	coli	10.1016/j.diagmicrobio.2006.01.0[illegible]
**acetaldehyde**	Escherichia	coli	10.1016/j.mimet.2005.09.003
**acetaldehyde**	Escherichia	coli	10.1016/j.mimet.2005.09.016
**acetaldehyde**	Escherichia	coli	10.1128/AEM.02069-07
**acetaldehyde**	Haemophilus	influenzae	10.1099/mic.0.062687-0
**acetaldehyde**	Stenotrophomonas	maltophilia	10.1088/1752-7155/10/3/037102
**acetaldehyde**	Stenotrophomonas	maltophilia	10.1088/1752-7155/10/3/037102
**acetaldehyde**	Stenotrophomonas	maltophilia	10.1088/1752-7155/10/3/037102
**acetaldehyde**	Neisseria	meningitidis	10.1016/j.diagmicrobio.2006.01.0[illegible]
**acetaldehyde**	Streptococcus	pneumoniae	10.1016/j.diagmicrobio.2006.01.0[illegible]
**acetaldehyde**	Streptococcus	pneumoniae	10.1016/j.mimet.2005.09.003
**acetaldehyde**	Streptococcus	pneumoniae	10.1016/j.mimet.2005.09.016
**acetaldehyde**	Streptococcus	pneumoniae	10.1099/mic.0.062687-0
**acetic acid**	Pseudomonas	aeruginosa	10.1016/j.diagmicrobio.2006.01.0[illegible]
**acetic acid**	Pseudomonas	aeruginosa	10.1016/j.jchromb.2012.05.038
**acetic acid**	Pseudomonas	aeruginosa	10.1088/1752-7155/10/3/037102
**acetic acid**	Pseudomonas	aeruginosa	10.1088/1752-7155/10/3/037102
**acetic acid**	Pseudomonas	aeruginosa	10.1088/1752-7155/10/3/037102
**acetic acid**	Pseudomonas	aeruginosa	10.1128/JCM.00392-10
**acetic acid**	Pseudomonas	aeruginosa	10.1128/mSphere.00843-20
**acetic acid**	Staphylococcus	aureus	10.1016/j.diagmicrobio.2006.01.0[illegible]
**acetic acid**	Staphylococcus	aureus	10.1016/j.mimet.2010.12.001
**acetic acid**	Staphylococcus	aureus	10.1016/j.mimet.2010.12.001
**acetic acid**	Staphylococcus	aureus	10.1038/s41598-020-74909-w
**acetic acid**	Staphylococcus	aureus	10.1038/s41598-020-74909-w
**acetic acid**	Staphylococcus	aureus	10.1088/1752-7155/10/3/037102
**acetic acid**	Staphylococcus	aureus	10.1088/1752-7155/10/3/037102
**acetic acid**	Staphylococcus	aureus	10.1088/1752-7155/10/3/037102
**acetic acid**	Staphylococcus	aureus	10.1128/JCM.00392-10
**acetic acid**	Staphylococcus	aureus	10.1186/1471-2180-12-113
**acetic acid**	Staphylococcus	aureus	10.3389/fmicb.2021.693075
**acetic acid**	Staphylococcus	aureus	10.3389/fmicb.2021.693075
**acetic acid**	Staphylococcus	aureus	10.3389/fmicb.2021.693075
**acetic acid**	Staphylococcus	aureus	10.3389/fmicb.2021.693075
**acetic acid**	Staphylococcus	aureus	10.3389/fmicb.2021.693075
**acetic acid**	Staphylococcus	aureus	10.3389/fmicb.2021.693075
**acetic acid**	Staphylococcus	aureus	10.3390/metabo10090347

**acetic acid**	Staphylococcus	aureus	10.3390/metabo10090347
**acetic acid**	Burkholderia	cepacia	10.1016/j.mimet.2010.12.001
**acetic acid**	Burkholderia	cepacia	10.1088/1752-7155/10/3/037102
**acetic acid**	Burkholderia	cepacia	10.1088/1752-7155/10/3/037102
**acetic acid**	Burkholderia	cepacia	10.1088/1752-7155/10/3/037102
**acetic acid**	Escherichia	coli	10.1016/j.diagmicrobio.2006.01.03
**acetic acid**	Escherichia	coli	10.1016/j.mimet.2005.09.003
**acetic acid**	Escherichia	coli	10.1016/j.mimet.2010.12.001
**acetic acid**	Escherichia	coli	10.1038/s41598-020-74909-w
**acetic acid**	Escherichia	coli	10.1038/s41598-020-74909-w
**acetic acid**	Escherichia	coli	10.1088/1752-7155/8/2/027106
**acetic acid**	Escherichia	coli	10.1088/1752-7155/8/2/027106
**acetic acid**	Escherichia	coli	10.1128/AEM.02069-07
**acetic acid**	Escherichia	coli	10.3389/fmicb.2021.693075
**acetic acid**	Escherichia	coli	10.3389/fmicb.2021.693075
**acetic acid**	Escherichia	coli	10.3389/fmicb.2021.693075
**acetic acid**	Escherichia	coli	10.3389/fmicb.2021.693075
**acetic acid**	Escherichia	coli	10.3389/fmicb.2021.693075
**acetic acid**	Escherichia	coli	10.3389/fmicb.2021.693075
**acetic acid**	Staphylococcus	epidermidis	10.1038/s41598-020-74909-w
**acetic acid**	Staphylococcus	epidermidis	10.1038/s41598-020-74909-w
**acetic acid**	Aspergillus	fumigatus	10.1088/1752-7155/6/1/016002
**acetic acid**	Haemophilus	influenzae	10.1016/j.jchromb.2009.05.028
**acetic acid**	Haemophilus	influenzae	10.1099/mic.0.062687-0
**acetic acid**	Stenotrophomonas	maltophilia	10.1088/1752-7155/10/3/037102
**acetic acid**	Stenotrophomonas	maltophilia	10.1088/1752-7155/10/3/037102
**acetic acid**	Stenotrophomonas	maltophilia	10.1088/1752-7155/10/3/037102
**acetic acid**	Neisseria	meningitidis	10.1016/j.diagmicrobio.2006.01.03
**acetic acid**	Neisseria	meningitidis	10.1016/j.mimet.2005.09.016
**acetic acid**	Proteus	mirabilis	10.1016/j.mimet.2010.12.001
**acetic acid**	Proteus	mirabilis	10.1016/j.mimet.2010.12.001
**acetic acid**	Klebsiella	pneumoniae	10.1088/1752-7155/10/2/027101
**acetic acid**	Streptococcus	pneumoniae	10.1016/j.diagmicrobio.2006.01.03
**acetic acid**	Streptococcus	pneumoniae	10.1016/j.jchromb.2009.05.028
**acetic acid**	Streptococcus	pneumoniae	10.1099/mic.0.062687-0
**acetoin**	Pseudomonas	aeruginosa	10.1088/1752-7155/10/3/037102
**acetoin**	Pseudomonas	aeruginosa	10.1088/1752-7155/10/3/037102
**acetoin**	Pseudomonas	aeruginosa	10.1088/1752-7155/10/3/037102
**acetoin**	Pseudomonas	aeruginosa	10.1128/mSphere.00843-20
**acetoin**	Pseudomonas	aeruginosa	10.3389/fmicb.2021.693075
**acetoin**	Pseudomonas	aeruginosa	10.3389/fmicb.2021.693075
**acetoin**	Staphylococcus	aureus	10.1002/jobm.201600505
**acetoin**	Staphylococcus	aureus	10.1016/j.jchromb.2009.05.028

**acetoin**	Staphylococcus	aureus	10.1016/S0378-4347(00)80760-4
**acetoin**	Staphylococcus	aureus	10.1038/s41598-020-74909-w
**acetoin**	Staphylococcus	aureus	10.1038/s41598-020-74909-w
**acetoin**	Staphylococcus	aureus	10.1088/1752-7155/10/3/037102
**acetoin**	Staphylococcus	aureus	10.1088/1752-7155/10/3/037102
**acetoin**	Staphylococcus	aureus	10.1088/1752-7155/10/3/037102
**acetoin**	Staphylococcus	aureus	10.1186/1471-2180-12-113
**acetoin**	Staphylococcus	aureus	10.3389/fmicb.2021.693075
**acetoin**	Staphylococcus	aureus	10.3389/fmicb.2021.693075
**acetoin**	Staphylococcus	aureus	10.3389/fmicb.2021.693075
**acetoin**	Staphylococcus	aureus	10.3389/fmicb.2021.693075
**acetoin**	Staphylococcus	aureus	10.3389/fmicb.2021.693075
**acetoin**	Burkholderia	cepacia	10.1016/j.mimet.2010.12.001
**acetoin**	Burkholderia	cepacia	10.1088/1752-7155/10/3/037102
**acetoin**	Burkholderia	cepacia	10.1088/1752-7155/10/3/037102
**acetoin**	Burkholderia	cepacia	10.1088/1752-7155/10/3/037102
**acetoin**	Escherichia	coli	10.1002/jobm.201600505
**acetoin**	Escherichia	coli	10.1016/j.mimet.2010.12.001
**acetoin**	Escherichia	coli	10.1016/j.mimet.2010.12.001
**acetoin**	Escherichia	coli	10.1038/s41598-020-74909-w
**acetoin**	Escherichia	coli	10.1038/s41598-020-74909-w
**acetoin**	Escherichia	coli	10.1111/jam.15716
**acetoin**	Escherichia	coli	10.1111/jam.15716
**acetoin**	Escherichia	coli	10.3389/fmicb.2021.693075
**acetoin**	Escherichia	coli	10.3389/fmicb.2021.693075
**acetoin**	Escherichia	coli	10.3389/fmicb.2021.693075
**acetoin**	Staphylococcus	epidermidis	10.1038/s41598-020-74909-w
**acetoin**	Stenotrophomonas	maltophilia	10.1088/1752-7155/10/3/037102
**acetoin**	Stenotrophomonas	maltophilia	10.1088/1752-7155/10/3/037102
**acetoin**	Stenotrophomonas	maltophilia	10.1088/1752-7155/10/3/037102
**acetoin**	Proteus	mirabilis	10.1016/j.mimet.2010.12.001
**acetoin**	Proteus	mirabilis	10.1016/j.mimet.2010.12.001
**acetoin**	Klebsiella	pneumoniae	10.1088/1752-7155/10/2/027101
**acetoin**	Streptococcus	pneumoniae	10.1016/j.jchromb.2018.08.032
**acetoin**	Streptococcus	pneumoniae	10.1016/j.jchromb.2018.08.032
**acetoin**	Streptococcus	pneumoniae	10.1016/j.jchromb.2018.08.032
**acetoin**	Streptococcus	pneumoniae	10.1016/j.jchromb.2018.08.032
**acetoin**	Streptococcus	pneumoniae	10.1016/j.jchromb.2018.08.032
**acetoin**	Streptococcus	pneumoniae	10.1016/j.jchromb.2018.08.032
**acetoin**	Streptococcus	pneumoniae	10.1016/j.jchromb.2018.08.032
**acetoin**	Streptococcus	pyogenes	10.1016/j.mimet.2010.12.001
**acetone**	Pseudomonas	aeruginosa	10.1007/s00253-013-4762-8
**acetone**	Pseudomonas	aeruginosa	10.1007/s00253-013-4762-8

**acetone**	Pseudomonas	aeruginosa	10.1016/j.diagmicrobio.2006.01.03
**acetone**	Pseudomonas	aeruginosa	10.1088/1752-7155/10/3/037102
**acetone**	Pseudomonas	aeruginosa	10.1088/1752-7155/10/3/037102
**acetone**	Pseudomonas	aeruginosa	10.1088/1752-7155/10/3/037102
**acetone**	Pseudomonas	aeruginosa	10.1088/1752-7155/10/4/047102
**acetone**	Pseudomonas	aeruginosa	10.1128/JCM.00392-10
**acetone**	Staphylococcus	aureus	10.1016/j.diagmicrobio.2006.01.03
**acetone**	Staphylococcus	aureus	10.1016/j.mimet.2005.09.016
**acetone**	Staphylococcus	aureus	10.1088/1752-7155/10/3/037102
**acetone**	Staphylococcus	aureus	10.1088/1752-7155/10/3/037102
**acetone**	Staphylococcus	aureus	10.1088/1752-7155/10/3/037102
**acetone**	Staphylococcus	aureus	10.1088/1752-7155/8/2/027106
**acetone**	Staphylococcus	aureus	10.1128/JCM.00392-10
**acetone**	Mycobacterium	bovis	10.1371/journal.pone.0194348
**acetone**	Burkholderia	cepacia	10.1088/1752-7155/10/3/037102
**acetone**	Burkholderia	cepacia	10.1088/1752-7155/10/3/037102
**acetone**	Burkholderia	cepacia	10.1088/1752-7155/10/3/037102
**acetone**	Escherichia	coli	10.1007/s00216-009-2758-0
**acetone**	Escherichia	coli	10.1016/j.diagmicrobio.2006.01.03
**acetone**	Escherichia	coli	10.1016/j.mimet.2005.09.016
**acetone**	Escherichia	coli	10.1016/j.mimet.2010.12.001
**acetone**	Escherichia	coli	10.1128/AEM.02069-07
**acetone**	Escherichia	coli	10.3390/antibiotics9110797
**acetone**	Escherichia	coli	10.3390/antibiotics9110797
**acetone**	Escherichia	coli	10.3390/antibiotics9110797
**acetone**	Staphylococcus	epidermidis	10.3390/metabo10090347
**acetone**	Enterococcus	faecalis	10.1016/j.mimet.2010.12.001
**acetone**	Aspergillus	fumigatus	10.1039/C4AY01217H
**acetone**	Stenotrophomonas	maltophilia	10.1088/1752-7155/10/3/037102
**acetone**	Stenotrophomonas	maltophilia	10.1088/1752-7155/10/3/037102
**acetone**	Stenotrophomonas	maltophilia	10.1088/1752-7155/10/3/037102
**acetone**	Neisseria	meningitidis	10.1016/j.diagmicrobio.2006.01.03
**acetone**	Streptococcus	pneumoniae	10.1016/j.diagmicrobio.2006.01.03
**acetone**	Streptococcus	pneumoniae	10.1016/j.mimet.2005.09.016
**acetone**	Streptococcus	pneumoniae	10.1099/mic.0.062687-0
**acetonitrile**	Pseudomonas	aeruginosa	10.1002/ppul.20170
**acetonitrile**	Pseudomonas	aeruginosa	10.1002/ppul.20170
**acetonitrile**	Pseudomonas	aeruginosa	10.1128/JCM.00392-10
**acetonitrile**	Staphylococcus	aureus	10.1128/JCM.00392-10
**acetonitrile**	Escherichia	coli	10.1128/JCM.00392-10
**acetonitrile**	Klebsiella	pneumoniae	10.1111/jam.13372
**acetophenone**	Pseudomonas	aeruginosa	10.1016/j.jchromb.2012.05.038
**acetophenone**	Pseudomonas	aeruginosa	10.1088/1752-7155/10/4/047102

**acetophenone**	Escherichia	coli	10.1111/jam.15716
**acetophenone**	Escherichia	coli	10.1111/jam.15716
**acetophenone**	Escherichia	coli	10.3390/antibiotics9110797
**acoradiene**	Aspergillus	terreus	10.1093/cid/ciu725
**allyl butyrate**	Escherichia	coli	10.3390/antibiotics9110797
**alpha-bergamotene**	Aspergillus	fumigatus	10.1093/cid/ciu725
**alpha-bergamotene**	Aspergillus	fumigatus	10.1093/cid/ciu725
**alpha-bergamotene**	Aspergillus	fumigatus	10.1128/EC.00074-14
**alpha-curcumene**	Aspergillus	fumigatus	10.1128/EC.00074-14
**alpha-elemene**	Aspergillus	terreus	10.1093/cid/ciu725
**alpha-farnesene**	Aspergillus	fumigatus	10.1088/1752-7155/6/1/016002
**alpha-farnesene**	Aspergillus	fumigatus	10.1088/1752-7155/6/1/016002
**alpha-farnesene**	Aspergillus	fumigatus	10.1088/1752-7155/6/1/016002
**alpha-farnesene**	Aspergillus	fumigatus	10.1088/1752-7155/6/1/016002
**alpha-farnesene**	Aspergillus	fumigatus	10.1088/1752-7155/6/1/016002
**alpha-farnesene**	Aspergillus	fumigatus	10.1088/1752-7155/6/1/016002
**alpha-Methylionone**	Candida	albicans	10.1038/srep27441
**alpha-Methylionone**	Penicillium	chrysogenum	10.1038/srep27441
**alpha-Methylionone**	Aspergillus	niger	10.1038/srep27441
**alpha-methylstyrene**	Klebsiella	pneumoniae	10.1111/jam.13372
**alpha-patchoulene**	Aspergillus	fumigatus	10.1128/EC.00074-14
**alpha-phellandrene**	Aspergillus	fumigatus	10.1128/EC.00074-14
**alpha-pinene**	Aspergillus	fumigatus	10.1039/c8an00841h
**alpha-pinene**	Aspergillus	fumigatus	10.1039/c8an00841h
**alpha-pinene**	Aspergillus	fumigatus	10.1093/cid/ciu725
**alpha-pinene**	Aspergillus	fumigatus	10.1093/cid/ciu725
**alpha-pinene**	Aspergillus	fumigatus	10.1128/EC.00074-14
**ammonia**	Pseudomonas	aeruginosa	10.1002/ppul.20170
**ammonia**	Pseudomonas	aeruginosa	10.1002/ppul.20170
**ammonia**	Pseudomonas	aeruginosa	10.1007/s00253-013-4762-8
**ammonia**	Pseudomonas	aeruginosa	10.1007/s00253-013-4762-8
**ammonia**	Pseudomonas	aeruginosa	10.1016/j.diagmicrobio.2006.01.03[illegible]
**ammonia**	Pseudomonas	aeruginosa	10.1016/j.mimet.2010.12.001
**ammonia**	Pseudomonas	aeruginosa	10.1016/j.mimet.2010.12.001
**ammonia**	Pseudomonas	aeruginosa	10.1088/1752-7155/10/3/037102
**ammonia**	Pseudomonas	aeruginosa	10.1088/1752-7155/10/3/037102
**ammonia**	Pseudomonas	aeruginosa	10.1088/1752-7155/10/3/037102
**ammonia**	Pseudomonas	aeruginosa	10.1111/j.1365-2672.2012.05414.[illegible]
**ammonia**	Staphylococcus	aureus	10.1016/j.diagmicrobio.2006.01.03[illegible]
**ammonia**	Staphylococcus	aureus	10.1016/j.mimet.2005.09.016
**ammonia**	Staphylococcus	aureus	10.1088/1752-7155/10/3/037102
**ammonia**	Staphylococcus	aureus	10.1088/1752-7155/10/3/037102
**ammonia**	Staphylococcus	aureus	10.1088/1752-7155/10/3/037102

**ammonia**	Burkholderia	cepacia	10.1016/j.mimet.2010.12.001
**ammonia**	Burkholderia	cepacia	10.1088/1752-7155/10/3/037102
**ammonia**	Burkholderia	cepacia	10.1088/1752-7155/10/3/037102
**ammonia**	Burkholderia	cepacia	10.1088/1752-7155/10/3/037102
**ammonia**	Escherichia	coli	10.1007/s00253-013-4762-8
**ammonia**	Escherichia	coli	10.1007/s00253-013-4762-8
**ammonia**	Escherichia	coli	10.1016/j.diagmicrobio.2006.01.03
**ammonia**	Escherichia	coli	10.1111/j.1365-2672.2012.05414.x
**ammonia**	Aspergillus	fumigatus	10.1039/C4AY01217H
**ammonia**	Stenotrophomonas	maltophilia	10.1088/1752-7155/10/3/037102
**ammonia**	Stenotrophomonas	maltophilia	10.1088/1752-7155/10/3/037102
**ammonia**	Stenotrophomonas	maltophilia	10.1088/1752-7155/10/3/037102
**ammonia**	Neisseria	meningitidis	10.1016/j.diagmicrobio.2006.01.03
**ammonia**	Neisseria	meningitidis	10.1016/j.mimet.2005.09.016
**ammonia**	Streptococcus	pneumoniae	10.1016/j.diagmicrobio.2006.01.03
**anisole**	Mycobacterium	avium	10.1088/1752-7163/aa6e06
**anisole**	Mycobacterium	bovis	10.1088/1752-7163/aa6e06
**anisole**	Mycobacterium	intracellulare	10.1088/1752-7163/aa6e06
**anisole**	Mycobacterium	xenopi	10.1088/1752-7163/aa6e06
**azacyclotridecan-2-one**	Aspergillus	fumigatus	10.1088/1752-7155/10/1/016002
**azulene**	Mycobacterium	tuberculosis	10.1088/1752-7163/aacd18
**benzaldehyde**	Candida	albicans	10.1038/srep27441
**benzaldehyde**	Staphylococcus	aureus	10.1186/1471-2180-12-113
**benzaldehyde**	Moraxella	catarrhalis	10.1016/j.jchromb.2009.05.028
**benzaldehyde**	Penicillium	chrysogenum	10.1038/srep27441
**benzaldehyde**	Escherichia	coli	10.1038/s41598-020-74909-w
**benzaldehyde**	Escherichia	coli	10.3390/antibiotics9110797
**benzaldehyde**	Escherichia	coli	10.3390/antibiotics9110797
**benzaldehyde**	Staphylococcus	epidermidis	10.3390/metabo10090347
**benzaldehyde**	Haemophilus	influenzae	10.1016/j.jchromb.2009.05.028
**benzaldehyde**	Aspergillus	niger	10.1038/srep27441
**benzaldehyde**	Streptococcus	pneumoniae	10.1016/j.jchromb.2009.05.028
**benzaldehyde**	Streptococcus	pneumoniae	10.1016/j.jchromb.2018.08.032
**benzaldehyde**	Streptococcus	pneumoniae	10.1016/j.jchromb.2018.08.032
**benzaldehyde**	Streptococcus	pneumoniae	10.1016/j.jchromb.2018.08.032
**benzaldehyde**	Streptococcus	pneumoniae	10.1016/j.jchromb.2018.08.032
**benzaldehyde**	Streptococcus	pneumoniae	10.1016/j.jchromb.2018.08.032
**benzaldehyde**	Streptococcus	pneumoniae	10.1016/j.jchromb.2018.08.032
**benzaldehyde**	Streptococcus	pneumoniae	10.1016/j.jchromb.2018.08.032
**benzene**	Candida	albicans	10.1038/srep27441
**benzene**	Staphylococcus	aureus	10.1109/JSEN.2009.2035671
**benzene**	Staphylococcus	aureus	10.1109/JSEN.2009.2035671
**benzene**	Penicillium	chrysogenum	10.1038/srep27441

**benzene**	Aspergillus	niger	10.1038/srep27441
**benzene**	Klebsiella	pneumoniae	10.1111/jam.13372
**benzene**	Streptococcus	pneumoniae	10.1016/j.jchromb.2018.08.032
**benzene**	Streptococcus	pneumoniae	10.1016/j.jchromb.2018.08.032
**benzene**	Streptococcus	pneumoniae	10.1016/j.jchromb.2018.08.032
**benzene**	Streptococcus	pneumoniae	10.1016/j.jchromb.2018.08.032
**benzene**	Streptococcus	pneumoniae	10.1016/j.jchromb.2018.08.032
**benzene**	Streptococcus	pneumoniae	10.1016/j.jchromb.2018.08.032
**benzene**	Streptococcus	pneumoniae	10.1016/j.jchromb.2018.08.032
**benzene, (1-methyldecyl)-**	Escherichia	coli	10.1111/jam.15716
**benzene, 1-(1,5-dimethylhexyl)-4-methyl-**	Aspergillus	fumigatus	10.1128/EC.00074-14
**benzonitrile**	Pseudomonas	aeruginosa	10.1007/s00253-012-3924-4
**benzonitrile**	Pseudomonas	aeruginosa	10.1016/j.jchromb.2012.05.038
**benzonitrile**	Staphylococcus	aureus	10.3390/metabo10090347
**benzonitrile**	Enterobacter	cloacae	10.1007/s00253-012-3924-4
**benzonitrile**	Escherichia	coli	10.1007/s00253-012-3924-4
**benzonitrile**	Staphylococcus	epidermidis	10.1007/s00253-012-3924-4
**benzonitrile**	Staphylococcus	epidermidis	10.3390/metabo10090347
**benzonitrile**	Staphylococcus	epidermidis	10.3390/metabo10090347
**benzonitrile**	Serratia	marcescens	10.1007/s00253-012-3924-4
**benzonitrile**	Proteus	mirabilis	10.1007/s00253-012-3924-4
**benzonitrile**	Streptococcus	pneumoniae	10.1007/s00253-012-3924-4
**benzothiazole**	Escherichia	coli	10.1111/jam.15716
**benzothiazole**	Stenotrophomonas	maltophilia	10.3390/metabo11110773
**benzoxazole**	Pseudomonas	aeruginosa	10.1016/j.jchromb.2012.05.038
**benzyl alcohol**	Candida	albicans	10.3390/metabo12050432
**benzyl alcohol**	Candida	albicans	10.3390/metabo12050432
**benzyl alcohol**	Staphylococcus	aureus	10.1038/s41598-020-74909-w
**benzyl alcohol**	Moraxella	catarrhalis	10.1016/j.jchromb.2009.05.028
**benzyl alcohol**	Escherichia	coli	10.1002/jssc.201800684
**benzyl alcohol**	Escherichia	coli	10.1038/s41598-020-74909-w
**benzyl alcohol**	Escherichia	coli	10.1038/s41598-020-74909-w
**benzyl alcohol**	Escherichia	coli	10.1111/jam.15716
**benzyl alcohol**	Escherichia	coli	10.1111/jam.15716
**benzyl alcohol**	Escherichia	coli	10.3390/antibiotics9110797
**benzyl alcohol**	Haemophilus	influenzae	10.1016/j.jchromb.2009.05.028
**benzyl alcohol**	Streptococcus	pneumoniae	10.1016/j.jchromb.2009.05.028
**benzyl formate**	Mycobacterium	intracellulare	10.1088/1752-7163/aa6e06
**benzyl methyl sulfide**	Proteus	mirabilis	10.1007/s00253-012-3924-4
**benzylideneacetone**	Streptococcus	pneumoniae	10.3390/metabo11110773
**bergamotene**	Aspergillus	fumigatus	10.1093/cid/ciu725
**bergamotene**	Aspergillus	fumigatus	10.1093/cid/ciu725
**bergamotene**	Aspergillus	fumigatus	10.1128/EC.00074-14

**beta-himachalene**	Aspergillus	fumigatus	10.1039/c8an00841h
**beta-hydroxyisovaleric acid**	Aspergillus	fumigatus	10.1128/EC.00074-14
**beta-hydroxyisovaleric acid**	Mycobacterium	tuberculosis	10.1088/1752-7163/aacd18
**beta-phellandrene**	Aspergillus	fumigatus	10.1088/1752-7155/6/1/016002
**beta-phellandrene**	Aspergillus	fumigatus	10.1088/1752-7155/6/1/016002
**beta-phellandrene**	Aspergillus	fumigatus	10.1088/1752-7155/6/1/016002
**beta-phellandrene**	Mycobacterium	tuberculosis	10.1088/1752-7163/aacd18
**beta-pinene**	Aspergillus	fumigatus	10.1093/cid/ciu725
**beta-pinene**	Aspergillus	fumigatus	10.1093/cid/ciu725
**beta-santalene**	Aspergillus	fumigatus	10.1128/EC.00074-14
**beta-spathulene**	Streptococcus	pneumoniae	10.3390/metabo11110773
**beta-vatirenene**	Aspergillus	fumigatus	10.1128/EC.00074-14
**beta-vatirenene**	Aspergillus	fumigatus	10.1128/EC.00074-14
**biphenyl**	Candida	albicans	10.1038/srep27441
**biphenyl**	Penicillium	chrysogenum	10.1038/srep27441
**biphenyl**	Aspergillus	niger	10.1038/srep27441
**bornyl acetate**	Candida	albicans	10.1038/srep27441
**bornyl acetate**	Penicillium	chrysogenum	10.1038/srep27441
**bornyl acetate**	Aspergillus	niger	10.1038/srep27441
**butane**	Pseudomonas	aeruginosa	10.1186/1471-2180-12-113
**butane**	Streptococcus	pneumoniae	10.1099/mic.0.062687-0
**butenal**	Pseudomonas	aeruginosa	10.1088/1752-7155/10/3/037102
**butenal**	Pseudomonas	aeruginosa	10.1088/1752-7155/10/3/037102
**butenal**	Pseudomonas	aeruginosa	10.1088/1752-7155/10/3/037102
**butenal**	Staphylococcus	aureus	10.1088/1752-7155/10/3/037102
**butenal**	Staphylococcus	aureus	10.1088/1752-7155/10/3/037102
**butenal**	Staphylococcus	aureus	10.1088/1752-7155/10/3/037102
**butenal**	Burkholderia	cepacia	10.1088/1752-7155/10/3/037102
**butenal**	Burkholderia	cepacia	10.1088/1752-7155/10/3/037102
**butenal**	Burkholderia	cepacia	10.1088/1752-7155/10/3/037102
**butenal**	Stenotrophomonas	maltophilia	10.1088/1752-7155/10/3/037102
**butenal**	Stenotrophomonas	maltophilia	10.1088/1752-7155/10/3/037102
**butenal**	Stenotrophomonas	maltophilia	10.1088/1752-7155/10/3/037102
**butyl 2-methylbutyrate**	Staphylococcus	aureus	10.1093/chromsci/bmt042
**butyl 2-methylbutyrate**	Staphylococcus	aureus	10.3390/metabo10090347
**butyl acetate**	Staphylococcus	aureus	10.1186/1471-2180-12-113
**butyl acetate**	Staphylococcus	aureus	10.3390/metabo10090347
**butyl acetate**	Escherichia	coli	10.3390/antibiotics9110797
**butyl isobutyrate**	Staphylococcus	aureus	10.3390/metabo10090347
**butyl isobutyrate**	Escherichia	coli	10.3390/antibiotics9110797
**butyl propionate**	Staphylococcus	aureus	10.3390/metabo10090347
**butyl propionate**	Escherichia	coli	10.3390/antibiotics9110797
**butyraldehyde**	Pseudomonas	aeruginosa	10.1088/1752-7155/10/1/016002

**butyraldehyde**	Pseudomonas	aeruginosa	10.1088/1752-7155/10/3/037102
**butyraldehyde**	Pseudomonas	aeruginosa	10.1088/1752-7155/10/3/037102
**butyraldehyde**	Pseudomonas	aeruginosa	10.1088/1752-7155/10/3/037102
**butyraldehyde**	Staphylococcus	aureus	10.1088/1752-7155/10/3/037102
**butyraldehyde**	Staphylococcus	aureus	10.1088/1752-7155/10/3/037102
**butyraldehyde**	Staphylococcus	aureus	10.1088/1752-7155/10/3/037102
**butyraldehyde**	Burkholderia	cepacia	10.1088/1752-7155/10/3/037102
**butyraldehyde**	Burkholderia	cepacia	10.1088/1752-7155/10/3/037102
**butyraldehyde**	Burkholderia	cepacia	10.1088/1752-7155/10/3/037102
**butyraldehyde**	Escherichia	coli	10.3390/antibiotics9110797
**butyraldehyde**	Haemophilus	influenzae	10.1099/mic.0.062687-0
**butyraldehyde**	Stenotrophomonas	maltophilia	10.1088/1752-7155/10/3/037102
**butyraldehyde**	Stenotrophomonas	maltophilia	10.1088/1752-7155/10/3/037102
**butyraldehyde**	Stenotrophomonas	maltophilia	10.1088/1752-7155/10/3/037102
**butyric acid**	Pseudomonas	aeruginosa	10.1088/1752-7155/10/3/037102
**butyric acid**	Pseudomonas	aeruginosa	10.1088/1752-7155/10/3/037102
**butyric acid**	Pseudomonas	aeruginosa	10.1088/1752-7155/10/3/037102
**butyric acid**	Pseudomonas	aeruginosa	10.1186/s13568-022-01367-0
**butyric acid**	Staphylococcus	aureus	10.1016/j.jchromb.2009.05.028
**butyric acid**	Staphylococcus	aureus	10.1016/j.mimet.2010.12.001
**butyric acid**	Staphylococcus	aureus	10.1016/j.mimet.2010.12.001
**butyric acid**	Staphylococcus	aureus	10.1088/1752-7155/10/3/037102
**butyric acid**	Staphylococcus	aureus	10.1088/1752-7155/10/3/037102
**butyric acid**	Staphylococcus	aureus	10.1088/1752-7155/10/3/037102
**butyric acid**	Staphylococcus	aureus	10.1093/chromsci/bmt042
**butyric acid**	Burkholderia	cepacia	10.1016/j.mimet.2010.12.001
**butyric acid**	Burkholderia	cepacia	10.1088/1752-7155/10/3/037102
**butyric acid**	Burkholderia	cepacia	10.1088/1752-7155/10/3/037102
**butyric acid**	Burkholderia	cepacia	10.1088/1752-7155/10/3/037102
**butyric acid**	Escherichia	coli	10.1016/j.mimet.2010.12.001
**butyric acid**	Escherichia	coli	10.1016/j.mimet.2010.12.001
**butyric acid**	Escherichia	coli	10.1093/chromsci/bmt042
**butyric acid**	Escherichia	coli	10.3390/antibiotics9110797
**butyric acid**	Stenotrophomonas	maltophilia	10.1088/1752-7155/10/3/037102
**butyric acid**	Stenotrophomonas	maltophilia	10.1088/1752-7155/10/3/037102
**butyric acid**	Stenotrophomonas	maltophilia	10.1088/1752-7155/10/3/037102
**butyric acid**	Proteus	mirabilis	10.1016/j.mimet.2010.12.001
**butyric acid**	Proteus	mirabilis	10.1016/j.mimet.2010.12.001
**butyric acid**	Streptococcus	pyogenes	10.1016/j.mimet.2010.12.001
**butyrophenone**	Pseudomonas	aeruginosa	10.1016/j.jchromb.2012.05.038
**camphene**	Aspergillus	fumigatus	10.1039/c8an00841h
**camphene**	Aspergillus	fumigatus	10.1039/c8an00841h
**camphene**	Aspergillus	fumigatus	10.1128/EC.00074-14

**carbon dioxide**	Aspergillus	fumigatus	10.1128/EC.00074-14
**carbon disulfide**	Pseudomonas	aeruginosa	10.1088/1752-7155/10/3/037102
**carbon disulfide**	Pseudomonas	aeruginosa	10.1088/1752-7155/10/3/037102
**carbon disulfide**	Pseudomonas	aeruginosa	10.1088/1752-7155/10/3/037102
**carbon disulfide**	Staphylococcus	aureus	10.1088/1752-7155/10/3/037102
**carbon disulfide**	Staphylococcus	aureus	10.1088/1752-7155/10/3/037102
**carbon disulfide**	Staphylococcus	aureus	10.1088/1752-7155/10/3/037102
**carbon disulfide**	Burkholderia	cepacia	10.1088/1752-7155/10/3/037102
**carbon disulfide**	Burkholderia	cepacia	10.1088/1752-7155/10/3/037102
**carbon disulfide**	Burkholderia	cepacia	10.1088/1752-7155/10/3/037102
**carbon disulfide**	Haemophilus	influenzae	10.1099/mic.0.062687-0
**carbon disulfide**	Stenotrophomonas	maltophilia	10.1088/1752-7155/10/3/037102
**carbon disulfide**	Stenotrophomonas	maltophilia	10.1088/1752-7155/10/3/037102
**carbon disulfide**	Stenotrophomonas	maltophilia	10.1088/1752-7155/10/3/037102
**carbon disulfide**	Klebsiella	pneumoniae	10.1088/1752-7155/10/2/027101
**carbon disulfide**	Streptococcus	pneumoniae	10.1099/mic.0.062687-0
**carbon disulfide**	Pseudomonas	putida	10.1016/S0045-6535(97)00209-9
**cedr-8(15)-ene**	Aspergillus	fumigatus	10.1128/EC.00074-14
**cis-1,4-dimethylcyclohexane**	Mycobacterium	tuberculosis	10.1016/j.tube.2006.03.004
**cis-1,4-dimethylcyclohexane**	Mycobacterium	tuberculosis	10.1016/j.tube.2006.03.004
**cis-2-butene**	Streptococcus	pneumoniae	10.1099/mic.0.062687-0
**citral**	Aspergillus	fumigatus	10.1128/EC.00074-14
**crotonaldehyde**	Streptococcus	pneumoniae	10.1099/mic.0.062687-0
**cumene**	Pseudomonas	aeruginosa	10.1128/mSphere.00843-20
**cyclohexanone**	Candida	albicans	10.1111/j.1439-0507.2011.02037.:
**cyclohexanone**	Staphylococcus	epidermidis	10.3390/metabo10090347
**cyclohexanone**	Aspergillus	fumigatus	10.1111/j.1439-0507.2011.02037.:
**cyclohexanone**	Mycobacterium	tuberculosis	10.5588/ijtld.11.0576
**cyclohexyl isothiocyanate**	Staphylococcus	aureus	10.3390/metabo10090347
**cyclohexyl isothiocyanate**	Staphylococcus	aureus	10.3390/metabo10090347
**cyclohexyl isothiocyanate**	Staphylococcus	epidermidis	10.3390/metabo10090347
**cycloisolongifolene, 8,9-dehydro-**	Aspergillus	fumigatus	10.1128/EC.00074-14
**cyclopentanol**	Pseudomonas	aeruginosa	10.1007/s11306-018-1357-5
**cyclopentanol**	Pseudomonas	aeruginosa	10.1088/1752-7163/aa8efc
**cyclopentanol**	Enterobacter	cloacae	10.1007/s11306-018-1357-5
**cyclopentanone**	Pseudomonas	aeruginosa	10.1007/s11306-018-1357-5
**cyclopentanone**	Pseudomonas	aeruginosa	10.1088/1752-7163/aa8efc
**cyclopentanone**	Enterobacter	cloacae	10.1007/s11306-018-1357-5
**cyclopentanone**	Escherichia	coli	10.1088/1752-7163/aa8efc
**dec-5-en-1-ol acetic acid**	Staphylococcus	aureus	10.3390/metabo10090347
**dec-5-en-1-ol acetic acid**	Staphylococcus	epidermidis	10.3390/metabo10090347
**decanal**	Pseudomonas	aeruginosa	10.1128/mSphere.00843-20
**decanal**	Candida	albicans	10.1038/srep27441

**decanal**	Acinetobacter	baumannii	10.1088/1752-7155/10/2/027102
**decanal**	Penicillium	chrysogenum	10.1038/srep27441
**decanal**	Escherichia	coli	10.1038/s41598-020-74909-w
**decanal**	Escherichia	coli	10.1038/s41598-020-74909-w
**decanal**	Escherichia	coli	10.1111/jam.15716
**decanal**	Aspergillus	niger	10.1038/srep27441
**decane**	Pseudomonas	aeruginosa	10.1038/s41598-020-74909-w
**decane**	Pseudomonas	aeruginosa	10.1088/1752-7155/10/4/047102
**decane**	Pseudomonas	aeruginosa	10.3389/fmicb.2021.693075
**decane**	Pseudomonas	aeruginosa	10.3389/fmicb.2021.693075
**decane**	Pseudomonas	aeruginosa	10.3389/fmicb.2021.693075
**decane**	Pseudomonas	aeruginosa	10.3389/fmicb.2021.693075
**decane**	Pseudomonas	aeruginosa	10.3389/fmicb.2021.693075
**decane**	Staphylococcus	aureus	10.3389/fmicb.2021.693075
**decane**	Staphylococcus	aureus	10.3389/fmicb.2021.693075
**decane**	Staphylococcus	aureus	10.3389/fmicb.2021.693075
**decane**	Staphylococcus	aureus	10.3389/fmicb.2021.693075
**decane**	Staphylococcus	aureus	10.3389/fmicb.2021.693075
**decane**	Staphylococcus	aureus	10.3390/metabo10090347
**decane**	Staphylococcus	aureus	10.3390/metabo10090347
**decane**	Staphylococcus	aureus	10.3390/metabo10090347
**decane**	Escherichia	coli	10.3389/fmicb.2021.693075
**decane**	Escherichia	coli	10.3389/fmicb.2021.693075
**decane**	Escherichia	coli	10.3389/fmicb.2021.693075
**decane**	Escherichia	coli	10.3389/fmicb.2021.693075
**decane**	Escherichia	coli	10.3389/fmicb.2021.693075
**decane**	Staphylococcus	epidermidis	10.1038/s41598-020-74909-w
**decane**	Staphylococcus	epidermidis	10.1038/s41598-020-74909-w
**decane**	Staphylococcus	epidermidis	10.3390/metabo10090347
**decane**	Staphylococcus	epidermidis	10.3390/metabo10090347
**decane**	Staphylococcus	epidermidis	10.3390/metabo10090347
**decane**	Staphylococcus	epidermidis	10.3390/metabo10090347
**decane, 4-ethyl-**	Mycobacterium	tuberculosis	10.1088/1752-7163/aacd18
**decyl acetate**	Escherichia	coli	10.1093/chromsci/bmt042
**decyl acetate**	Escherichia	coli	10.1093/chromsci/bmt042
**decyl acetate**	Escherichia	coli	10.3389/fmicb.2021.693075
**decyl acetate**	Escherichia	coli	10.3389/fmicb.2021.693075
**decyl acetate**	Escherichia	coli	10.3389/fmicb.2021.693075
**delta-valerolactone**	Escherichia	coli	10.1111/jam.15716
**diacetone alcohol**	Klebsiella	pneumoniae	10.1111/jam.13372
**dibenzofuran**	Escherichia	coli	10.1111/jam.15716
**dibenzofuran**	Escherichia	coli	10.1111/jam.15716
**diethanolamine**	Staphylococcus	aureus	10.3390/metabo11110773

**diethyl ether**	Klebsiella	pneumoniae	10.1111/jam.13372
**diethylene glycol monoethyl ether**	Escherichia	coli	10.1002/jssc.201800684
**dihydrocamphene carbinol**	Pseudomonas	aeruginosa	10.1128/mSphere.00843-20
**dihydrofarnesol**	Candida	albicans	10.3390/metabo12050432
**dihydrofarnesol**	Candida	albicans	10.3390/metabo12050432
**Dimethyl 2,4-dimethylpentanedioate**	Mycobacterium	bovis	10.1088/1752-7163/aa6e06
**dimethyl 2-octyladipate**	Aspergillus	fumigatus	10.1088/1752-7155/6/1/016002
**dimethyl 2-octyladipate**	Aspergillus	fumigatus	10.1088/1752-7155/6/1/016002
**dimethyl 2-octyladipate**	Aspergillus	fumigatus	10.1088/1752-7155/6/1/016002
**dimethyl 2-octyladipate**	Aspergillus	fumigatus	10.1088/1752-7155/6/1/016002
**dimethyl 2-octyladipate**	Aspergillus	fumigatus	10.1088/1752-7155/6/1/016002
**dimethyl 2-octyladipate**	Aspergillus	fumigatus	10.1088/1752-7155/6/1/016002
**dimethyl disulfide**	Pseudomonas	aeruginosa	10.1002/ppul.20170
**dimethyl disulfide**	Pseudomonas	aeruginosa	10.1002/ppul.20170
**dimethyl disulfide**	Pseudomonas	aeruginosa	10.1007/s00253-012-3924-4
**dimethyl disulfide**	Pseudomonas	aeruginosa	10.1016/j.diagmicrobio.2006.01.03
**dimethyl disulfide**	Pseudomonas	aeruginosa	10.1016/j.jchromb.2009.05.028
**dimethyl disulfide**	Pseudomonas	aeruginosa	10.1016/j.mimet.2010.12.001
**dimethyl disulfide**	Pseudomonas	aeruginosa	10.1016/S0045-6535(97)00209-9
**dimethyl disulfide**	Pseudomonas	aeruginosa	10.1016/S0378-4347(00)80760-4
**dimethyl disulfide**	Pseudomonas	aeruginosa	10.1016/S0378-4347(00)80760-4
**dimethyl disulfide**	Pseudomonas	aeruginosa	10.1038/s41598-020-74909-w
**dimethyl disulfide**	Pseudomonas	aeruginosa	10.1038/s41598-020-74909-w
**dimethyl disulfide**	Pseudomonas	aeruginosa	10.1088/1752-7155/10/1/016002
**dimethyl disulfide**	Pseudomonas	aeruginosa	10.1088/1752-7155/10/3/037102
**dimethyl disulfide**	Pseudomonas	aeruginosa	10.1088/1752-7155/10/3/037102
**dimethyl disulfide**	Pseudomonas	aeruginosa	10.1088/1752-7155/10/3/037102
**dimethyl disulfide**	Pseudomonas	aeruginosa	10.1088/1752-7155/10/4/047102
**dimethyl disulfide**	Pseudomonas	aeruginosa	10.1088/1752-7163/aa8efc
**dimethyl disulfide**	Pseudomonas	aeruginosa	10.1088/1752-7163/aa8efc
**dimethyl disulfide**	Pseudomonas	aeruginosa	10.1128/JCM.12.4.521-526.1980
**dimethyl disulfide**	Pseudomonas	aeruginosa	10.1128/JCM.12.4.521-526.1980
**dimethyl disulfide**	Pseudomonas	aeruginosa	10.1128/JCM.12.4.521-526.1980
**dimethyl disulfide**	Pseudomonas	aeruginosa	10.1128/JCM.12.4.521-526.1980
**dimethyl disulfide**	Pseudomonas	aeruginosa	10.1128/JCM.12.4.521-526.1980
**dimethyl disulfide**	Pseudomonas	aeruginosa	10.1128/JCM.12.4.521-526.1980
**dimethyl disulfide**	Pseudomonas	aeruginosa	10.1128/JCM.12.4.521-526.1980
**dimethyl disulfide**	Pseudomonas	aeruginosa	10.1128/JCM.12.4.521-526.1980
**dimethyl disulfide**	Pseudomonas	aeruginosa	10.1128/JCM.12.4.521-526.1980
**dimethyl disulfide**	Pseudomonas	aeruginosa	10.1128/JCM.12.4.521-526.1980
**dimethyl disulfide**	Pseudomonas	aeruginosa	10.1128/JCM.12.4.521-526.1980
**dimethyl disulfide**	Pseudomonas	aeruginosa	10.1128/mSphere.00843-20
**dimethyl disulfide**	Pseudomonas	aeruginosa	10.1186/1471-2180-12-113

**dimethyl disulfide**	Candida	albicans	10.1111/myc.12442
**dimethyl disulfide**	Staphylococcus	aureus	10.1016/j.diagmicrobio.2006.01.03
**dimethyl disulfide**	Staphylococcus	aureus	10.1016/S0378-4347(00)80760-4
**dimethyl disulfide**	Staphylococcus	aureus	10.1038/s41598-020-74909-w
**dimethyl disulfide**	Staphylococcus	aureus	10.1038/s41598-020-74909-w
**dimethyl disulfide**	Staphylococcus	aureus	10.1088/1752-7155/10/3/037102
**dimethyl disulfide**	Staphylococcus	aureus	10.1088/1752-7155/10/3/037102
**dimethyl disulfide**	Staphylococcus	aureus	10.1088/1752-7155/10/3/037102
**dimethyl disulfide**	Staphylococcus	aureus	10.1088/1752-7155/8/2/027106
**dimethyl disulfide**	Staphylococcus	aureus	10.1088/1752-7163/aa8efc
**dimethyl disulfide**	Staphylococcus	aureus	10.1088/1752-7163/aa8efc
**dimethyl disulfide**	Burkholderia	cepacia	10.1088/1752-7155/10/3/037102
**dimethyl disulfide**	Burkholderia	cepacia	10.1088/1752-7155/10/3/037102
**dimethyl disulfide**	Burkholderia	cepacia	10.1088/1752-7155/10/3/037102
**dimethyl disulfide**	Burkholderia	cepacia	10.1128/JCM.12.4.521-526.1980
**dimethyl disulfide**	Enterobacter	cloacae	10.1007/s00253-012-3924-4
**dimethyl disulfide**	Enterobacter	cloacae	10.1016/S0045-6535(97)00209-9
**dimethyl disulfide**	Escherichia	coli	10.1007/s00253-012-3924-4
**dimethyl disulfide**	Escherichia	coli	10.1016/j.diagmicrobio.2006.01.03
**dimethyl disulfide**	Escherichia	coli	10.1016/j.mimet.2005.09.003
**dimethyl disulfide**	Escherichia	coli	10.1016/j.mimet.2010.12.001
**dimethyl disulfide**	Escherichia	coli	10.1038/s41598-020-74909-w
**dimethyl disulfide**	Escherichia	coli	10.1038/s41598-020-74909-w
**dimethyl disulfide**	Escherichia	coli	10.1088/1752-7163/aa8efc
**dimethyl disulfide**	Escherichia	coli	10.1088/1752-7163/aa8efc
**dimethyl disulfide**	Escherichia	coli	10.1111/jam.15716
**dimethyl disulfide**	Escherichia	coli	10.1111/jam.15716
**dimethyl disulfide**	Escherichia	coli	10.3390/antibiotics9110797
**dimethyl disulfide**	Escherichia	coli	10.3390/antibiotics9110797
**dimethyl disulfide**	Staphylococcus	epidermidis	10.1038/s41598-020-74909-w
**dimethyl disulfide**	Staphylococcus	epidermidis	10.1038/s41598-020-74909-w
**dimethyl disulfide**	Staphylococcus	epidermidis	10.3390/metabo10090347
**dimethyl disulfide**	Staphylococcus	epidermidis	10.3390/metabo10090347
**dimethyl disulfide**	Enterococcus	faecalis	10.1016/j.mimet.2010.12.001
**dimethyl disulfide**	Pseudomonas	fluorescens	10.1016/S0045-6535(97)00209-9
**dimethyl disulfide**	Aspergillus	fumigatus	10.1039/C4AY01217H
**dimethyl disulfide**	Haemophilus	influenzae	10.1099/mic.0.062687-0
**dimethyl disulfide**	Serratia	liquefaciens	10.1016/S0045-6535(97)00209-9
**dimethyl disulfide**	Stenotrophomonas	maltophilia	10.1088/1752-7155/10/3/037102
**dimethyl disulfide**	Stenotrophomonas	maltophilia	10.1088/1752-7155/10/3/037102
**dimethyl disulfide**	Stenotrophomonas	maltophilia	10.1088/1752-7155/10/3/037102
**dimethyl disulfide**	Stenotrophomonas	maltophilia	10.1088/1752-7155/9/2/027104
**dimethyl disulfide**	Stenotrophomonas	maltophilia	10.1128/JCM.12.4.521-526.1980

**dimethyl disulfide**	Serratia	marcescens	10.1007/s00253-012-3924-4
**dimethyl disulfide**	Neisseria	meningitidis	10.1016/j.diagmicrobio.2006.01.0[illegible]
**dimethyl disulfide**	Proteus	mirabilis	10.1007/s00253-012-3924-4
**dimethyl disulfide**	Proteus	mirabilis	10.1016/j.mimet.2010.12.001
**dimethyl disulfide**	Proteus	mirabilis	10.1016/j.mimet.2010.12.001
**dimethyl disulfide**	Klebsiella	pneumoniae	10.1016/S0378-4347(00)80760-4
**dimethyl disulfide**	Klebsiella	pneumoniae	10.1016/S0378-4347(00)80760-4
**dimethyl disulfide**	Streptococcus	pneumoniae	10.1016/j.diagmicrobio.2006.01.0[illegible]
**dimethyl disulfide**	Streptococcus	pneumoniae	10.1016/j.mimet.2005.09.016
**dimethyl disulfide**	Streptococcus	pneumoniae	10.1099/mic.0.062687-0
**dimethyl disulfide**	Pseudomonas	putida	10.1016/S0045-6535(97)00209-9
**dimethyl disulfide**	Pseudomonas	putida	10.1128/JCM.12.4.521-526.1980
**dimethyl disulfide**	Shewanella	putrefaciens	10.1128/JCM.12.4.521-526.1980
**dimethyl disulfide**	Stenotrophomonas	rhizophila	10.1088/1752-7155/9/2/027104
**dimethyl sulfide**	Pseudomonas	aeruginosa	10.1016/j.diagmicrobio.2006.01.0[illegible]
**dimethyl sulfide**	Pseudomonas	aeruginosa	10.1016/j.jchromb.2009.05.028
**dimethyl sulfide**	Pseudomonas	aeruginosa	10.1016/j.jchromb.2012.05.038
**dimethyl sulfide**	Pseudomonas	aeruginosa	10.1016/j.mimet.2005.09.003
**dimethyl sulfide**	Pseudomonas	aeruginosa	10.1088/1752-7155/10/3/037102
**dimethyl sulfide**	Pseudomonas	aeruginosa	10.1088/1752-7155/10/3/037102
**dimethyl sulfide**	Pseudomonas	aeruginosa	10.1088/1752-7155/10/3/037102
**dimethyl sulfide**	Pseudomonas	aeruginosa	10.1088/1752-7155/10/4/047102
**dimethyl sulfide**	Pseudomonas	aeruginosa	10.1128/mSphere.00843-20
**dimethyl sulfide**	Pseudomonas	aeruginosa	10.1186/1471-2180-12-113
**dimethyl sulfide**	Staphylococcus	aureus	10.1016/j.diagmicrobio.2006.01.0[illegible]
**dimethyl sulfide**	Staphylococcus	aureus	10.1088/1752-7155/10/3/037102
**dimethyl sulfide**	Staphylococcus	aureus	10.1088/1752-7155/10/3/037102
**dimethyl sulfide**	Staphylococcus	aureus	10.1088/1752-7155/10/3/037102
**dimethyl sulfide**	Mycobacterium	bovis	10.1111/j.1574-6968.2011.02493.[illegible]
**dimethyl sulfide**	Burkholderia	cepacia	10.1088/1752-7155/10/3/037102
**dimethyl sulfide**	Burkholderia	cepacia	10.1088/1752-7155/10/3/037102
**dimethyl sulfide**	Burkholderia	cepacia	10.1088/1752-7155/10/3/037102
**dimethyl sulfide**	Escherichia	coli	10.1016/j.diagmicrobio.2006.01.0[illegible]
**dimethyl sulfide**	Escherichia	coli	10.1016/j.mimet.2005.09.003
**dimethyl sulfide**	Escherichia	coli	10.1111/jam.15716
**dimethyl sulfide**	Escherichia	coli	10.1111/jam.15716
**dimethyl sulfide**	Escherichia	coli	10.3390/antibiotics9110797
**dimethyl sulfide**	Aspergillus	fumigatus	10.1039/C4AY01217H
**dimethyl sulfide**	Haemophilus	influenzae	10.1099/mic.0.062687-0
**dimethyl sulfide**	Stenotrophomonas	maltophilia	10.1088/1752-7155/10/3/037102
**dimethyl sulfide**	Stenotrophomonas	maltophilia	10.1088/1752-7155/10/3/037102
**dimethyl sulfide**	Stenotrophomonas	maltophilia	10.1088/1752-7155/10/3/037102
**dimethyl sulfide**	Neisseria	meningitidis	10.1016/j.diagmicrobio.2006.01.0[illegible]

**dimethyl sulfide**	Klebsiella	pneumoniae	10.1088/1752-7155/10/2/027101
**dimethyl sulfide**	Streptococcus	pneumoniae	10.1016/j.diagmicrobio.2006.01.0[illegible]
**dimethyl sulfide**	Streptococcus	pneumoniae	10.1016/j.mimet.2005.09.003
**dimethyl sulfide**	Streptococcus	pneumoniae	10.1099/mic.0.062687-0
**dimethyl sulfide**	Pseudomonas	putida	10.1016/S0045-6535(97)00209-9
**dimethyl trisulfide**	Pseudomonas	aeruginosa	10.1016/S0045-6535(97)00209-9
**dimethyl trisulfide**	Pseudomonas	aeruginosa	10.1038/s41598-020-74909-w
**dimethyl trisulfide**	Pseudomonas	aeruginosa	10.1088/1752-7155/10/1/016002
**dimethyl trisulfide**	Pseudomonas	aeruginosa	10.1088/1752-7155/10/3/037102
**dimethyl trisulfide**	Pseudomonas	aeruginosa	10.1088/1752-7155/10/3/037102
**dimethyl trisulfide**	Pseudomonas	aeruginosa	10.1088/1752-7155/10/3/037102
**dimethyl trisulfide**	Pseudomonas	aeruginosa	10.1088/1752-7155/10/4/047102
**dimethyl trisulfide**	Pseudomonas	aeruginosa	10.1088/1752-7163/aa8efc
**dimethyl trisulfide**	Pseudomonas	aeruginosa	10.1088/1752-7163/aa8efc
**dimethyl trisulfide**	Pseudomonas	aeruginosa	10.1128/JCM.12.4.521-526.1980
**dimethyl trisulfide**	Pseudomonas	aeruginosa	10.1128/JCM.12.4.521-526.1980
**dimethyl trisulfide**	Pseudomonas	aeruginosa	10.1128/JCM.12.4.521-526.1980
**dimethyl trisulfide**	Pseudomonas	aeruginosa	10.1128/JCM.12.4.521-526.1980
**dimethyl trisulfide**	Pseudomonas	aeruginosa	10.1128/JCM.12.4.521-526.1980
**dimethyl trisulfide**	Pseudomonas	aeruginosa	10.1128/JCM.12.4.521-526.1980
**dimethyl trisulfide**	Pseudomonas	aeruginosa	10.1128/JCM.12.4.521-526.1980
**dimethyl trisulfide**	Pseudomonas	aeruginosa	10.1128/JCM.12.4.521-526.1980
**dimethyl trisulfide**	Pseudomonas	aeruginosa	10.1128/JCM.12.4.521-526.1980
**dimethyl trisulfide**	Pseudomonas	aeruginosa	10.1128/JCM.12.4.521-526.1980
**dimethyl trisulfide**	Pseudomonas	aeruginosa	10.1128/JCM.12.4.521-526.1980
**dimethyl trisulfide**	Pseudomonas	aeruginosa	10.1186/1471-2180-12-113
**dimethyl trisulfide**	Staphylococcus	aureus	10.1038/s41598-020-74909-w
**dimethyl trisulfide**	Staphylococcus	aureus	10.1088/1752-7155/10/3/037102
**dimethyl trisulfide**	Staphylococcus	aureus	10.1088/1752-7155/10/3/037102
**dimethyl trisulfide**	Staphylococcus	aureus	10.1088/1752-7155/10/3/037102
**dimethyl trisulfide**	Staphylococcus	aureus	10.1088/1752-7155/8/2/027106
**dimethyl trisulfide**	Staphylococcus	aureus	10.1088/1752-7163/aa8efc
**dimethyl trisulfide**	Staphylococcus	aureus	10.1088/1752-7163/aa8efc
**dimethyl trisulfide**	Staphylococcus	aureus	10.3390/metabo10090347
**dimethyl trisulfide**	Staphylococcus	aureus	10.3390/metabo10090347
**dimethyl trisulfide**	Burkholderia	cepacia	10.1088/1752-7155/10/3/037102
**dimethyl trisulfide**	Burkholderia	cepacia	10.1088/1752-7155/10/3/037102
**dimethyl trisulfide**	Burkholderia	cepacia	10.1088/1752-7155/10/3/037102
**dimethyl trisulfide**	Burkholderia	cepacia	10.1128/JCM.12.4.521-526.1980
**dimethyl trisulfide**	Enterobacter	cloacae	10.1016/S0045-6535(97)00209-9
**dimethyl trisulfide**	Escherichia	coli	10.1007/s00253-012-3924-4
**dimethyl trisulfide**	Escherichia	coli	10.1038/s41598-020-74909-w
**dimethyl trisulfide**	Escherichia	coli	10.1088/1752-7163/aa8efc

**dimethyl trisulfide**	Escherichia	coli	10.1088/1752-7163/aa8efc
**dimethyl trisulfide**	Escherichia	coli	10.1111/jam.15716
**dimethyl trisulfide**	Escherichia	coli	10.1111/jam.15716
**dimethyl trisulfide**	Escherichia	coli	10.3390/antibiotics9110797
**dimethyl trisulfide**	Staphylococcus	epidermidis	10.3390/metabo10090347
**dimethyl trisulfide**	Staphylococcus	epidermidis	10.3390/metabo10090347
**dimethyl trisulfide**	Staphylococcus	epidermidis	10.3390/metabo10090347
**dimethyl trisulfide**	Pseudomonas	fluorescens	10.1128/JCM.12.4.521-526.1980
**dimethyl trisulfide**	Haemophilus	influenzae	10.1099/mic.0.062687-0
**dimethyl trisulfide**	Stenotrophomonas	maltophilia	10.1088/1752-7155/10/3/037102
**dimethyl trisulfide**	Stenotrophomonas	maltophilia	10.1088/1752-7155/10/3/037102
**dimethyl trisulfide**	Stenotrophomonas	maltophilia	10.1088/1752-7155/10/3/037102
**dimethyl trisulfide**	Stenotrophomonas	maltophilia	10.1128/JCM.12.4.521-526.1980
**dimethyl trisulfide**	Serratia	marcescens	10.1007/s00253-012-3924-4
**dimethyl trisulfide**	Proteus	mirabilis	10.1007/s00253-012-3924-4
**dimethyl trisulfide**	Streptococcus	pneumoniae	10.1099/mic.0.062687-0
**dimethyl trisulfide**	Pseudomonas	putida	10.1016/S0045-6535(97)00209-9
**dimethyl trisulfide**	Pseudomonas	putida	10.1128/JCM.12.4.521-526.1980
**dimethyl trisulfide**	Shewanella	putrefaciens	10.1128/JCM.12.4.521-526.1980
**d-limonene**	Escherichia	coli	10.3390/antibiotics9110797
**d-limonene**	Aspergillus	fumigatus	10.1128/EC.00074-14
**dodecanal**	Candida	albicans	10.1038/srep27441
**dodecanal**	Penicillium	chrysogenum	10.1038/srep27441
**dodecanal**	Aspergillus	niger	10.1038/srep27441
**dodecane**	Pseudomonas	aeruginosa	10.1007/s00253-013-4762-8
**dodecane**	Pseudomonas	aeruginosa	10.1007/s00253-013-4762-8
**dodecane**	Pseudomonas	aeruginosa	10.3389/fmicb.2021.693075
**dodecane**	Staphylococcus	aureus	10.3390/metabo10090347
**dodecane**	Escherichia	coli	10.1111/jam.15716
**dodecane**	Escherichia	coli	10.3389/fmicb.2021.693075
**dodecane**	Staphylococcus	epidermidis	10.1038/s41598-020-74909-w
**dodecane**	Mycobacterium	tuberculosis	10.5588/ijtld.11.0576
**elixene**	Aspergillus	terreus	10.1093/cid/ciu725
**epi-beta-santalene**	Aspergillus	fumigatus	10.1128/EC.00074-14
**ethanol**	Pseudomonas	aeruginosa	10.1016/j.diagmicrobio.2006.01.0[illegible]
**ethanol**	Pseudomonas	aeruginosa	10.1016/j.mimet.2010.12.001
**ethanol**	Pseudomonas	aeruginosa	10.1016/j.mimet.2010.12.001
**ethanol**	Pseudomonas	aeruginosa	10.1088/1752-7155/10/3/037102
**ethanol**	Pseudomonas	aeruginosa	10.1088/1752-7155/10/3/037102
**ethanol**	Pseudomonas	aeruginosa	10.1088/1752-7155/10/3/037102
**ethanol**	Pseudomonas	aeruginosa	10.1128/JCM.00392-10
**ethanol**	Pseudomonas	aeruginosa	10.1186/1471-2180-12-113
**ethanol**	Pseudomonas	aeruginosa	10.1186/s13568-022-01367-0

**ethanol**	Pseudomonas	aeruginosa	10.3389/fmicb.2021.693075
**ethanol**	Pseudomonas	aeruginosa	10.3389/fmicb.2021.693075
**ethanol**	Pseudomonas	aeruginosa	10.3389/fmicb.2021.693075
**ethanol**	Pseudomonas	aeruginosa	10.3389/fmicb.2021.693075
**ethanol**	Pseudomonas	aeruginosa	10.3389/fmicb.2021.693075
**ethanol**	Pseudomonas	aeruginosa	10.3389/fmicb.2021.693075
**ethanol**	Candida	albicans	10.3390/metabo12050432
**ethanol**	Candida	albicans	10.3390/metabo12050432
**ethanol**	Staphylococcus	aureus	10.1016/j.diagmicrobio.2006.01.03
**ethanol**	Staphylococcus	aureus	10.1016/j.mimet.2005.09.003
**ethanol**	Staphylococcus	aureus	10.1016/j.mimet.2010.12.001
**ethanol**	Staphylococcus	aureus	10.1016/j.mimet.2010.12.001
**ethanol**	Staphylococcus	aureus	10.1088/1752-7155/10/3/037102
**ethanol**	Staphylococcus	aureus	10.1088/1752-7155/10/3/037102
**ethanol**	Staphylococcus	aureus	10.1088/1752-7155/10/3/037102
**ethanol**	Staphylococcus	aureus	10.1109/JSEN.2009.2035671
**ethanol**	Staphylococcus	aureus	10.1109/JSEN.2009.2035671
**ethanol**	Staphylococcus	aureus	10.1128/JCM.00392-10
**ethanol**	Staphylococcus	aureus	10.1186/1471-2180-12-113
**ethanol**	Staphylococcus	aureus	10.1186/s13568-022-01367-0
**ethanol**	Staphylococcus	aureus	10.3389/fmicb.2021.693075
**ethanol**	Staphylococcus	aureus	10.3389/fmicb.2021.693075
**ethanol**	Staphylococcus	aureus	10.3389/fmicb.2021.693075
**ethanol**	Staphylococcus	aureus	10.3389/fmicb.2021.693075
**ethanol**	Staphylococcus	aureus	10.3389/fmicb.2021.693075
**ethanol**	Burkholderia	cepacia	10.1016/j.mimet.2010.12.001
**ethanol**	Burkholderia	cepacia	10.1088/1752-7155/10/3/037102
**ethanol**	Burkholderia	cepacia	10.1088/1752-7155/10/3/037102
**ethanol**	Burkholderia	cepacia	10.1088/1752-7155/10/3/037102
**ethanol**	Escherichia	coli	10.1007/s00216-009-2758-0
**ethanol**	Escherichia	coli	10.1007/s00253-013-4762-8
**ethanol**	Escherichia	coli	10.1007/s00253-013-4762-8
**ethanol**	Escherichia	coli	10.1016/j.diagmicrobio.2006.01.03
**ethanol**	Escherichia	coli	10.1016/j.mimet.2005.09.003
**ethanol**	Escherichia	coli	10.1016/j.mimet.2005.09.016
**ethanol**	Escherichia	coli	10.1016/j.mimet.2010.12.001
**ethanol**	Escherichia	coli	10.1016/j.mimet.2010.12.001
**ethanol**	Escherichia	coli	10.1128/AEM.02069-07
**ethanol**	Escherichia	coli	10.1128/JCM.00392-10
**ethanol**	Escherichia	coli	10.1186/s13568-022-01367-0
**ethanol**	Escherichia	coli	10.3389/fmicb.2021.693075
**ethanol**	Escherichia	coli	10.3389/fmicb.2021.693075
**ethanol**	Escherichia	coli	10.3389/fmicb.2021.693075

**ethanol**	Escherichia	coli	10.3389/fmicb.2021.693075
**ethanol**	Escherichia	coli	10.3389/fmicb.2021.693075
**ethanol**	Escherichia	coli	10.3390/antibiotics9110797
**ethanol**	Escherichia	coli	10.3390/antibiotics9110797
**ethanol**	Enterococcus	faecalis	10.1016/j.mimet.2010.12.001
**ethanol**	Aspergillus	fumigatus	10.1039/C4AY01217H
**ethanol**	Aspergillus	fumigatus	10.1128/EC.00074-14
**ethanol**	Stenotrophomonas	maltophilia	10.1088/1752-7155/10/3/037102
**ethanol**	Stenotrophomonas	maltophilia	10.1088/1752-7155/10/3/037102
**ethanol**	Stenotrophomonas	maltophilia	10.1088/1752-7155/10/3/037102
**ethanol**	Neisseria	meningitidis	10.1016/j.diagmicrobio.2006.01.0[illegible]
**ethanol**	Proteus	mirabilis	10.1016/j.mimet.2010.12.001
**ethanol**	Proteus	mirabilis	10.1016/j.mimet.2010.12.001
**ethanol**	Klebsiella	pneumoniae	10.1088/1752-7155/10/2/027101
**ethanol**	Streptococcus	pneumoniae	10.1016/j.diagmicrobio.2006.01.0[illegible]
**ethanol**	Streptococcus	pneumoniae	10.1016/j.mimet.2005.09.003
**ethanol**	Streptococcus	pneumoniae	10.1016/j.mimet.2005.09.016
**ethanol**	Streptococcus	pneumoniae	10.1099/mic.0.062687-0
**ethanol**	Streptococcus	pyogenes	10.1016/j.mimet.2010.12.001
**ethyl 2-methylbutyrate**	Pseudomonas	aeruginosa	10.1186/1471-2180-12-113
**ethyl 2-methylbutyrate**	Candida	albicans	10.1111/myc.12442
**ethyl 2-methylbutyrate**	Staphylococcus	aureus	10.1038/s41598-020-74909-w
**ethyl 2-methylbutyrate**	Staphylococcus	aureus	10.1038/s41598-020-74909-w
**ethyl 2-methylbutyrate**	Staphylococcus	aureus	10.1093/chromsci/bmt042
**ethyl 2-methylbutyrate**	Staphylococcus	aureus	10.1093/chromsci/bmt042
**ethyl 2-methylbutyrate**	Staphylococcus	aureus	10.3389/fmicb.2021.693075
**ethyl 2-methylbutyrate**	Staphylococcus	aureus	10.3389/fmicb.2021.693075
**ethyl 2-methylbutyrate**	Staphylococcus	aureus	10.3389/fmicb.2021.693075
**ethyl 2-methylbutyrate**	Staphylococcus	aureus	10.3389/fmicb.2021.693075
**ethyl 2-methylbutyrate**	Staphylococcus	aureus	10.3389/fmicb.2021.693075
**ethyl 2-methylbutyrate**	Staphylococcus	epidermidis	10.1038/s41598-020-74909-w
**ethyl 2-methylbutyrate**	Staphylococcus	epidermidis	10.1038/s41598-020-74909-w
**ethyl 4-ethoxybenzoate**	Escherichia	coli	10.1002/jssc.201800684
**ethyl acetate**	Pseudomonas	aeruginosa	10.1186/1471-2180-12-113
**ethyl acetate**	Pseudomonas	aeruginosa	10.3389/fmicb.2021.693075
**ethyl acetate**	Pseudomonas	aeruginosa	10.3389/fmicb.2021.693075
**ethyl acetate**	Pseudomonas	aeruginosa	10.3389/fmicb.2021.693075
**ethyl acetate**	Pseudomonas	aeruginosa	10.3389/fmicb.2021.693075
**ethyl acetate**	Candida	albicans	10.1111/myc.12442
**ethyl acetate**	Candida	albicans	10.3390/metabo12050432
**ethyl acetate**	Staphylococcus	aureus	10.1186/1471-2180-12-113
**ethyl acetate**	Staphylococcus	aureus	10.3389/fmicb.2021.693075
**ethyl acetate**	Staphylococcus	aureus	10.3389/fmicb.2021.693075

**ethyl acetate**	Staphylococcus	aureus	10.3389/fmicb.2021.693075
**ethyl acetate**	Burkholderia	cepacia	10.1016/j.mimet.2010.12.001
**ethyl acetate**	Escherichia	coli	10.1016/j.mimet.2010.12.001
**ethyl acetate**	Escherichia	coli	10.1016/j.mimet.2010.12.001
**ethyl acetate**	Escherichia	coli	10.3389/fmicb.2021.693075
**ethyl acetate**	Escherichia	coli	10.3389/fmicb.2021.693075
**ethyl acetate**	Escherichia	coli	10.3389/fmicb.2021.693075
**ethyl acetate**	Escherichia	coli	10.3389/fmicb.2021.693075
**ethyl acetate**	Escherichia	coli	10.3389/fmicb.2021.693075
**ethyl acetate**	Escherichia	coli	10.3389/fmicb.2021.693075
**ethyl acetate**	Aspergillus	fumigatus	10.1039/c8an00841h
**ethyl acetate**	Aspergillus	fumigatus	10.1039/c8an00841h
**ethyl acetate**	Haemophilus	influenzae	10.1099/mic.0.062687-0
**ethyl acetate**	Proteus	mirabilis	10.1016/j.mimet.2010.12.001
**ethyl acetate**	Proteus	mirabilis	10.1016/j.mimet.2010.12.001
**ethyl acetate**	Streptococcus	pneumoniae	10.1099/mic.0.062687-0
**ethyl acetate**	Streptococcus	pyogenes	10.1016/j.mimet.2010.12.001
**ethyl benzoate**	Mycobacterium	tuberculosis	10.3762/bjoc.8.31
**ethyl butyrate**	Pseudomonas	aeruginosa	10.1016/j.mimet.2010.12.001
**ethyl butyrate**	Candida	albicans	10.3390/metabo12050432
**ethyl butyrate**	Staphylococcus	aureus	10.1016/j.mimet.2010.12.001
**ethyl butyrate**	Staphylococcus	aureus	10.1016/j.mimet.2010.12.001
**ethyl butyrate**	Burkholderia	cepacia	10.1016/j.mimet.2010.12.001
**ethyl butyrate**	Escherichia	coli	10.1016/j.mimet.2010.12.001
**ethyl butyrate**	Escherichia	coli	10.1016/j.mimet.2010.12.001
**ethyl butyrate**	Enterococcus	faecalis	10.1016/j.mimet.2010.12.001
**ethyl butyrate**	Proteus	mirabilis	10.1016/j.mimet.2010.12.001
**ethyl butyrate**	Proteus	mirabilis	10.1016/j.mimet.2010.12.001
**ethyl butyrate**	Streptococcus	pyogenes	10.1016/j.mimet.2010.12.001
**ethyl decanoate**	Escherichia	coli	10.3389/fmicb.2021.693075
**ethyl decanoate**	Escherichia	coli	10.3389/fmicb.2021.693075
**ethyl decanoate**	Escherichia	coli	10.3389/fmicb.2021.693075
**ethyl decanoate**	Escherichia	coli	10.3389/fmicb.2021.693075
**ethyl formate**	Staphylococcus	aureus	10.1186/1471-2180-12-113
**ethyl formate**	Klebsiella	pneumoniae	10.1111/jam.13372
**ethyl heptanoate**	Pseudomonas	aeruginosa	10.1186/s13568-022-01367-0
**ethyl isobutyl sulfide**	Mycobacterium	tuberculosis	10.1088/1752-7163/aacd18
**ethyl isobutyrate**	Candida	albicans	10.3390/metabo12050432
**ethyl isocyanate**	Mycobacterium	tuberculosis	10.5588/ijtld.11.0576
**ethyl isocyanide**	Escherichia	coli	10.3389/fmicb.2021.693075
**ethyl isocyanide**	Escherichia	coli	10.3389/fmicb.2021.693075
**ethyl isocyanide**	Escherichia	coli	10.3389/fmicb.2021.693075
**ethyl isocyanide**	Escherichia	coli	10.3389/fmicb.2021.693075

**ethyl isovalerate**	Staphylococcus	aureus	10.1186/1471-2180-12-113
**ethyl laurate**	Escherichia	coli	10.3389/fmicb.2021.693075
**ethyl laurate**	Escherichia	coli	10.3389/fmicb.2021.693075
**ethyl laurate**	Escherichia	coli	10.3389/fmicb.2021.693075
**ethyl myristate**	Escherichia	coli	10.3389/fmicb.2021.693075
**ethyl myristate**	Escherichia	coli	10.3389/fmicb.2021.693075
**ethyl octanoate**	Escherichia	coli	10.1038/s41598-020-74909-w
**ethyl octanoate**	Escherichia	coli	10.1038/s41598-020-74909-w
**ethyl phenylacetate**	Escherichia	coli	10.1002/jssc.201800684
**ethyl phenylacetate**	Escherichia	coli	10.1007/s00253-012-3924-4
**ethyl propionate**	Candida	albicans	10.3390/metabo12050432
**ethyl propionate**	Escherichia	coli	10.3389/fmicb.2021.693075
**ethylbenzene**	Escherichia	coli	10.3390/antibiotics9110797
**ethylbenzene**	Staphylococcus	epidermidis	10.3390/metabo10090347
**ethylbenzene**	Staphylococcus	epidermidis	10.3390/metabo10090347
**ethylene glycol**	Pseudomonas	aeruginosa	10.1128/JCM.00392-10
**ethylene glycol**	Staphylococcus	aureus	10.1128/JCM.00392-10
**ethylidenecyclopropane**	Pseudomonas	aeruginosa	10.1007/s11306-018-1357-5
**ethylidenecyclopropane**	Enterobacter	cloacae	10.1007/s11306-018-1357-5
**farnesal**	Streptococcus	pneumoniae	10.3390/metabo11110773
**farnesol**	Candida	albicans	10.1128/EC.00252-07
**farnesol**	Candida	albicans	10.3390/metabo12050432
**farnesol**	Candida	albicans	10.3390/metabo12050432
**formaldehyde**	Pseudomonas	aeruginosa	10.1016/j.mimet.2010.12.001
**formaldehyde**	Pseudomonas	aeruginosa	10.1016/j.mimet.2010.12.001
**formaldehyde**	Pseudomonas	aeruginosa	10.1088/1752-7155/10/3/037102
**formaldehyde**	Pseudomonas	aeruginosa	10.1088/1752-7155/10/3/037102
**formaldehyde**	Pseudomonas	aeruginosa	10.1088/1752-7155/10/3/037102
**formaldehyde**	Staphylococcus	aureus	10.1016/j.mimet.2010.12.001
**formaldehyde**	Staphylococcus	aureus	10.1016/j.mimet.2010.12.001
**formaldehyde**	Staphylococcus	aureus	10.1088/1752-7155/10/3/037102
**formaldehyde**	Staphylococcus	aureus	10.1088/1752-7155/10/3/037102
**formaldehyde**	Staphylococcus	aureus	10.1088/1752-7155/10/3/037102
**formaldehyde**	Burkholderia	cepacia	10.1016/j.mimet.2010.12.001
**formaldehyde**	Burkholderia	cepacia	10.1088/1752-7155/10/3/037102
**formaldehyde**	Burkholderia	cepacia	10.1088/1752-7155/10/3/037102
**formaldehyde**	Burkholderia	cepacia	10.1088/1752-7155/10/3/037102
**formaldehyde**	Escherichia	coli	10.1016/j.mimet.2005.09.003
**formaldehyde**	Escherichia	coli	10.1016/j.mimet.2010.12.001
**formaldehyde**	Escherichia	coli	10.1016/j.mimet.2010.12.001
**formaldehyde**	Enterococcus	faecalis	10.1016/j.mimet.2010.12.001
**formaldehyde**	Stenotrophomonas	maltophilia	10.1088/1752-7155/10/3/037102
**formaldehyde**	Stenotrophomonas	maltophilia	10.1088/1752-7155/10/3/037102

**formaldehyde**	Proteus	mirabilis	10.1016/j.mimet.2010.12.001
**formaldehyde**	Proteus	mirabilis	10.1016/j.mimet.2010.12.001
**formaldehyde**	Streptococcus	pneumoniae	10.1016/j.mimet.2005.09.003
**formaldehyde**	Streptococcus	pyogenes	10.1016/j.mimet.2010.12.001
**furan**	Haemophilus	influenzae	10.1099/mic.0.062687-0
**furan**	Klebsiella	pneumoniae	10.1111/jam.13372
**furan**	Streptococcus	pneumoniae	10.1099/mic.0.062687-0
**furfural**	Pseudomonas	aeruginosa	10.1088/1752-7155/10/1/016002
**furfural**	Pseudomonas	aeruginosa	10.1128/mSphere.00843-20
**furfuryl alcohol**	Staphylococcus	aureus	10.1088/1752-7163/aa8efc
**furfuryl alcohol**	Escherichia	coli	10.1088/1752-7163/aa8efc
**furfuryl alcohol**	Streptococcus	pneumoniae	10.3390/metabo11110773
**gamma-butyrolactone**	Candida	albicans	10.3390/metabo12050432
**gamma-butyrolactone**	Staphylococcus	aureus	10.3389/fmicb.2021.693075
**gamma-butyrolactone**	Staphylococcus	aureus	10.3389/fmicb.2021.693075
**gamma-butyrolactone**	Escherichia	coli	10.1002/jssc.201800684
**gamma-terpinene**	Aspergillus	fumigatus	10.1128/EC.00074-14
**gamma-valerolactone**	Mycobacterium	tuberculosis	10.3762/bjoc.8.31
**heptadecan-2-one**	Escherichia	coli	10.1111/jam.15716
**heptadecan-2-one**	Escherichia	coli	10.1111/jam.15716
**heptadecane**	Candida	albicans	10.1038/srep27441
**heptadecane**	Penicillium	chrysogenum	10.1038/srep27441
**heptadecane**	Aspergillus	niger	10.1038/srep27441
**heptanal**	Pseudomonas	aeruginosa	10.1088/1752-7155/10/3/037102
**heptanal**	Pseudomonas	aeruginosa	10.1088/1752-7155/10/3/037102
**heptanal**	Pseudomonas	aeruginosa	10.1088/1752-7155/10/3/037102
**heptanal**	Pseudomonas	aeruginosa	10.1128/mSphere.00843-20
**heptanal**	Candida	albicans	10.1038/srep27441
**heptanal**	Staphylococcus	aureus	10.1088/1752-7155/10/3/037102
**heptanal**	Staphylococcus	aureus	10.1088/1752-7155/10/3/037102
**heptanal**	Staphylococcus	aureus	10.1088/1752-7155/10/3/037102
**heptanal**	Burkholderia	cepacia	10.1088/1752-7155/10/3/037102
**heptanal**	Burkholderia	cepacia	10.1088/1752-7155/10/3/037102
**heptanal**	Burkholderia	cepacia	10.1088/1752-7155/10/3/037102
**heptanal**	Penicillium	chrysogenum	10.1038/srep27441
**heptanal**	Stenotrophomonas	maltophilia	10.1088/1752-7155/10/3/037102
**heptanal**	Stenotrophomonas	maltophilia	10.1088/1752-7155/10/3/037102
**heptanal**	Stenotrophomonas	maltophilia	10.1088/1752-7155/10/3/037102
**heptanal**	Aspergillus	niger	10.1038/srep27441
**heptanal**	Streptococcus	pneumoniae	10.1016/j.jchromb.2018.08.032
**heptanal**	Streptococcus	pneumoniae	10.1016/j.jchromb.2018.08.032
**heptanal**	Streptococcus	pneumoniae	10.1016/j.jchromb.2018.08.032
**heptanal**	Streptococcus	pneumoniae	10.1016/j.jchromb.2018.08.032

**heptanal**	Streptococcus	pneumoniae	10.1016/j.jchromb.2018.08.032
**heptanal**	Streptococcus	pneumoniae	10.1016/j.jchromb.2018.08.032
**heptanal**	Streptococcus	pneumoniae	10.1016/j.jchromb.2018.08.032
**heptanal**	Mycobacterium	tuberculosis	10.1088/1752-7163/aacd18
**heptane**	Mycobacterium	bovis	10.1371/journal.pone.0194348
**hexadecanal**	Escherichia	coli	10.3389/fmicb.2021.693075
**hexadecanal**	Escherichia	coli	10.3389/fmicb.2021.693075
**hexadecanal**	Escherichia	coli	10.3389/fmicb.2021.693075
**hexadecanal**	Escherichia	coli	10.3389/fmicb.2021.693075
**hexadecanal**	Escherichia	coli	10.3389/fmicb.2021.693075
**hexadecane**	Candida	albicans	10.1038/srep27441
**hexadecane**	Penicillium	chrysogenum	10.1038/srep27441
**hexadecane**	Escherichia	coli	10.1111/jam.15716
**hexadecane**	Aspergillus	niger	10.1038/srep27441
**hexanal**	Pseudomonas	aeruginosa	10.1088/1752-7155/10/1/016002
**hexanal**	Pseudomonas	aeruginosa	10.1088/1752-7155/10/3/037102
**hexanal**	Pseudomonas	aeruginosa	10.1088/1752-7155/10/3/037102
**hexanal**	Pseudomonas	aeruginosa	10.1088/1752-7155/10/3/037102
**hexanal**	Pseudomonas	aeruginosa	10.1128/mSphere.00843-20
**hexanal**	Candida	albicans	10.1038/srep27441
**hexanal**	Staphylococcus	aureus	10.1016/j.mimet.2005.09.003
**hexanal**	Staphylococcus	aureus	10.1088/1752-7155/10/3/037102
**hexanal**	Staphylococcus	aureus	10.1088/1752-7155/10/3/037102
**hexanal**	Staphylococcus	aureus	10.1088/1752-7155/10/3/037102
**hexanal**	Burkholderia	cepacia	10.1088/1752-7155/10/3/037102
**hexanal**	Burkholderia	cepacia	10.1088/1752-7155/10/3/037102
**hexanal**	Burkholderia	cepacia	10.1088/1752-7155/10/3/037102
**hexanal**	Penicillium	chrysogenum	10.1038/srep27441
**hexanal**	Escherichia	coli	10.1016/j.mimet.2005.09.003
**hexanal**	Stenotrophomonas	maltophilia	10.1088/1752-7155/10/3/037102
**hexanal**	Stenotrophomonas	maltophilia	10.1088/1752-7155/10/3/037102
**hexanal**	Stenotrophomonas	maltophilia	10.1088/1752-7155/10/3/037102
**hexanal**	Aspergillus	niger	10.1038/srep27441
**hexanal**	Streptococcus	pneumoniae	10.1016/j.mimet.2005.09.003
**hexane**	Mycobacterium	bovis	10.1371/journal.pone.0194348
**hexane**	Streptococcus	pneumoniae	10.1099/mic.0.062687-0
**hexanoic acid**	Pseudomonas	aeruginosa	10.1088/1752-7155/10/3/037102
**hexanoic acid**	Pseudomonas	aeruginosa	10.1088/1752-7155/10/3/037102
**hexanoic acid**	Pseudomonas	aeruginosa	10.1088/1752-7155/10/3/037102
**hexanoic acid**	Staphylococcus	aureus	10.1088/1752-7155/10/3/037102
**hexanoic acid**	Staphylococcus	aureus	10.1088/1752-7155/10/3/037102
**hexanoic acid**	Staphylococcus	aureus	10.1088/1752-7155/10/3/037102
**hexanoic acid**	Burkholderia	cepacia	10.1088/1752-7155/10/3/037102

**hexanoic acid**	Burkholderia	cepacia	10.1088/1752-7155/10/3/037102
**hexanoic acid**	Burkholderia	cepacia	10.1088/1752-7155/10/3/037102
**hexanoic acid**	Escherichia	coli	10.1111/jam.15716
**hexanoic acid**	Escherichia	coli	10.1111/jam.15716
**hexanoic acid**	Stenotrophomonas	maltophilia	10.1088/1752-7155/10/3/037102
**hexanoic acid**	Stenotrophomonas	maltophilia	10.1088/1752-7155/10/3/037102
**hexanoic acid**	Stenotrophomonas	maltophilia	10.1088/1752-7155/10/3/037102
**hydrogen cyanide**	Pseudomonas	aeruginosa	10.1002/ppul.20170
**hydrogen cyanide**	Pseudomonas	aeruginosa	10.1002/ppul.20170
**hydrogen cyanide**	Pseudomonas	aeruginosa	10.1016/j.jchromb.2012.05.038
**hydrogen cyanide**	Pseudomonas	aeruginosa	10.1088/1752-7155/10/3/037102
**hydrogen cyanide**	Pseudomonas	aeruginosa	10.1088/1752-7155/10/3/037102
**hydrogen cyanide**	Pseudomonas	aeruginosa	10.1088/1752-7155/10/3/037102
**hydrogen cyanide**	Staphylococcus	aureus	10.1088/1752-7155/10/3/037102
**hydrogen cyanide**	Staphylococcus	aureus	10.1088/1752-7155/10/3/037102
**hydrogen cyanide**	Staphylococcus	aureus	10.1088/1752-7155/10/3/037102
**hydrogen cyanide**	Burkholderia	cepacia	10.1088/1752-7155/10/3/037102
**hydrogen cyanide**	Burkholderia	cepacia	10.1088/1752-7155/10/3/037102
**hydrogen cyanide**	Burkholderia	cepacia	10.1088/1752-7155/10/3/037102
**hydrogen cyanide**	Stenotrophomonas	maltophilia	10.1088/1752-7155/10/3/037102
**hydrogen cyanide**	Stenotrophomonas	maltophilia	10.1088/1752-7155/10/3/037102
**hydrogen cyanide**	Stenotrophomonas	maltophilia	10.1088/1752-7155/10/3/037102
**hydrogen sulfide**	Pseudomonas	aeruginosa	10.1016/j.diagmicrobio.2006.01.03
**hydrogen sulfide**	Pseudomonas	aeruginosa	10.1016/j.mimet.2005.09.003
**hydrogen sulfide**	Pseudomonas	aeruginosa	10.1016/j.mimet.2010.12.001
**hydrogen sulfide**	Pseudomonas	aeruginosa	10.1016/j.mimet.2010.12.001
**hydrogen sulfide**	Pseudomonas	aeruginosa	10.1088/1752-7155/10/3/037102
**hydrogen sulfide**	Pseudomonas	aeruginosa	10.1088/1752-7155/10/3/037102
**hydrogen sulfide**	Pseudomonas	aeruginosa	10.1088/1752-7155/10/3/037102
**hydrogen sulfide**	Staphylococcus	aureus	10.1016/j.diagmicrobio.2006.01.03
**hydrogen sulfide**	Staphylococcus	aureus	10.1016/j.mimet.2005.09.003
**hydrogen sulfide**	Staphylococcus	aureus	10.1016/j.mimet.2010.12.001
**hydrogen sulfide**	Staphylococcus	aureus	10.1016/j.mimet.2010.12.001
**hydrogen sulfide**	Staphylococcus	aureus	10.1088/1752-7155/10/3/037102
**hydrogen sulfide**	Staphylococcus	aureus	10.1088/1752-7155/10/3/037102
**hydrogen sulfide**	Staphylococcus	aureus	10.1088/1752-7155/10/3/037102
**hydrogen sulfide**	Mycobacterium	bovis	10.1111/j.1574-6968.2011.02493.
**hydrogen sulfide**	Burkholderia	cepacia	10.1016/j.mimet.2010.12.001
**hydrogen sulfide**	Burkholderia	cepacia	10.1088/1752-7155/10/3/037102
**hydrogen sulfide**	Burkholderia	cepacia	10.1088/1752-7155/10/3/037102
**hydrogen sulfide**	Burkholderia	cepacia	10.1088/1752-7155/10/3/037102
**hydrogen sulfide**	Escherichia	coli	10.1016/j.diagmicrobio.2006.01.03
**hydrogen sulfide**	Escherichia	coli	10.1016/j.mimet.2005.09.003

**hydrogen sulfide**	Escherichia	coli	10.1016/j.mimet.2005.09.016
**hydrogen sulfide**	Escherichia	coli	10.1016/j.mimet.2010.12.001
**hydrogen sulfide**	Escherichia	coli	10.1016/j.mimet.2010.12.001
**hydrogen sulfide**	Enterococcus	faecalis	10.1016/j.mimet.2010.12.001
**hydrogen sulfide**	Stenotrophomonas	maltophilia	10.1088/1752-7155/10/3/037102
**hydrogen sulfide**	Stenotrophomonas	maltophilia	10.1088/1752-7155/10/3/037102
**hydrogen sulfide**	Stenotrophomonas	maltophilia	10.1088/1752-7155/10/3/037102
**hydrogen sulfide**	Neisseria	meningitidis	10.1016/j.mimet.2005.09.016
**hydrogen sulfide**	Proteus	mirabilis	10.1016/j.mimet.2010.12.001
**hydrogen sulfide**	Proteus	mirabilis	10.1016/j.mimet.2010.12.001
**hydrogen sulfide**	Streptococcus	pneumoniae	10.1016/j.diagmicrobio.2006.01.0[illegible]
**hydrogen sulfide**	Streptococcus	pneumoniae	10.1016/j.mimet.2005.09.016
**hydroxyacetone**	Staphylococcus	aureus	10.1038/s41598-020-74909-w
**hydroxyacetone**	Staphylococcus	aureus	10.1038/s41598-020-74909-w
**hydroxyacetone**	Staphylococcus	aureus	10.1186/1471-2180-12-113
**hydroxyacetone**	Escherichia	coli	10.1038/s41598-020-74909-w
**hydroxyacetone**	Escherichia	coli	10.1038/s41598-020-74909-w
**hydroxyacetone**	Staphylococcus	epidermidis	10.1038/s41598-020-74909-w
**hydroxyacetone**	Staphylococcus	epidermidis	10.1038/s41598-020-74909-w
**hydroxyacetone**	Aspergillus	fumigatus	10.1088/1752-7155/10/1/016002
**imidazole-4-methanol**	Streptococcus	pneumoniae	10.3390/metabo11110773
**iminodibenzyl**	Acinetobacter	baumannii	10.1088/1752-7155/10/2/027102
**indole**	Pseudomonas	aeruginosa	10.1111/j.1365-2672.2012.05414.[illegible]
**indole**	Pseudomonas	aeruginosa	10.1128/JCM.00392-10
**indole**	Staphylococcus	aureus	10.1002/jobm.201600505
**indole**	Staphylococcus	aureus	10.1016/j.mimet.2005.09.003
**indole**	Staphylococcus	aureus	10.1016/j.mimet.2010.12.001
**indole**	Burkholderia	cepacia	10.1016/j.mimet.2010.12.001
**indole**	Escherichia	coli	10.1002/jobm.201600505
**indole**	Escherichia	coli	10.1002/jssc.201800684
**indole**	Escherichia	coli	10.1007/s00253-012-3924-4
**indole**	Escherichia	coli	10.1007/s00253-013-4762-8
**indole**	Escherichia	coli	10.1007/s00253-013-4762-8
**indole**	Escherichia	coli	10.1016/j.mimet.2005.09.003
**indole**	Escherichia	coli	10.1016/j.mimet.2005.09.016
**indole**	Escherichia	coli	10.1016/j.mimet.2010.12.001
**indole**	Escherichia	coli	10.1016/j.mimet.2010.12.001
**indole**	Escherichia	coli	10.1038/s41598-020-74909-w
**indole**	Escherichia	coli	10.1038/s41598-020-74909-w
**indole**	Escherichia	coli	10.1088/1752-7155/8/2/027106
**indole**	Escherichia	coli	10.1088/1752-7155/8/2/027106
**indole**	Escherichia	coli	10.1088/1752-7163/aa8efc
**indole**	Escherichia	coli	10.1088/1752-7163/aa8efc

**indole**	Escherichia	coli	10.1093/chromsci/bmt042
**indole**	Escherichia	coli	10.1093/chromsci/bmt042
**indole**	Escherichia	coli	10.1111/j.1365-2672.2012.05414.[illegible]
**indole**	Escherichia	coli	10.1111/jam.15716
**indole**	Escherichia	coli	10.1111/jam.15716
**indole**	Escherichia	coli	10.1128/AEM.02069-07
**indole**	Escherichia	coli	10.1128/JCM.00392-10
**indole**	Escherichia	coli	10.3389/fmicb.2021.693075
**indole**	Escherichia	coli	10.3389/fmicb.2021.693075
**indole**	Escherichia	coli	10.3389/fmicb.2021.693075
**indole**	Escherichia	coli	10.3389/fmicb.2021.693075
**indole**	Escherichia	coli	10.3389/fmicb.2021.693075
**indole**	Escherichia	coli	10.3389/fmicb.2021.693075
**indole**	Escherichia	coli	10.3390/antibiotics9110797
**indole**	Haemophilus	influenzae	10.1016/j.jchromb.2009.05.028
**indole**	Streptococcus	pneumoniae	10.1016/j.mimet.2005.09.003
**isoamyl acetate**	Pseudomonas	aeruginosa	10.1016/S0378-4347(00)80760-4
**isoamyl acetate**	Pseudomonas	aeruginosa	10.1016/S0378-4347(00)80760-4
**isoamyl acetate**	Candida	albicans	10.3390/metabo12050432
**isoamyl acetate**	Candida	albicans	10.3390/metabo12050432
**isoamyl acetate**	Staphylococcus	aureus	10.1038/s41598-020-74909-w
**isoamyl acetate**	Staphylococcus	aureus	10.1093/chromsci/bmt042
**isoamyl acetate**	Staphylococcus	aureus	10.1093/chromsci/bmt042
**isoamyl acetate**	Staphylococcus	aureus	10.1186/1471-2180-12-113
**isoamyl acetate**	Staphylococcus	aureus	10.1186/s13568-022-01367-0
**isoamyl acetate**	Staphylococcus	aureus	10.3389/fmicb.2021.693075
**isoamyl acetate**	Staphylococcus	aureus	10.3389/fmicb.2021.693075
**isoamyl acetate**	Staphylococcus	aureus	10.3389/fmicb.2021.693075
**isoamyl acetate**	Staphylococcus	aureus	10.3389/fmicb.2021.693075
**isoamyl acetate**	Klebsiella	pneumoniae	10.1016/S0378-4347(00)80760-4
**isoamyl acetate**	Klebsiella	pneumoniae	10.1016/S0378-4347(00)80760-4
**isoamyl alcohol**	Pseudomonas	aeruginosa	10.1007/s00253-012-3924-4
**isoamyl alcohol**	Pseudomonas	aeruginosa	10.1016/j.jchromb.2012.05.038
**isoamyl alcohol**	Pseudomonas	aeruginosa	10.1016/S0378-4347(00)80760-4
**isoamyl alcohol**	Pseudomonas	aeruginosa	10.1016/S0378-4347(00)80760-4
**isoamyl alcohol**	Pseudomonas	aeruginosa	10.1038/s41598-020-74909-w
**isoamyl alcohol**	Pseudomonas	aeruginosa	10.1038/s41598-020-74909-w
**isoamyl alcohol**	Pseudomonas	aeruginosa	10.1128/JCM.12.4.521-526.1980
**isoamyl alcohol**	Pseudomonas	aeruginosa	10.1128/JCM.12.4.521-526.1980
**isoamyl alcohol**	Pseudomonas	aeruginosa	10.1128/JCM.12.4.521-526.1980
**isoamyl alcohol**	Pseudomonas	aeruginosa	10.1128/JCM.12.4.521-526.1980
**isoamyl alcohol**	Pseudomonas	aeruginosa	10.1128/JCM.12.4.521-526.1980
**isoamyl alcohol**	Pseudomonas	aeruginosa	10.1128/JCM.12.4.521-526.1980

**isoamyl alcohol**	Pseudomonas	aeruginosa	10.1128/JCM.12.4.521-526.1980
**isoamyl alcohol**	Pseudomonas	aeruginosa	10.1128/JCM.12.4.521-526.1980
**isoamyl alcohol**	Pseudomonas	aeruginosa	10.1128/JCM.12.4.521-526.1980
**isoamyl alcohol**	Pseudomonas	aeruginosa	10.1128/JCM.12.4.521-526.1980
**isoamyl alcohol**	Pseudomonas	aeruginosa	10.1128/JCM.12.4.521-526.1980
**isoamyl alcohol**	Pseudomonas	aeruginosa	10.1128/mSphere.00843-20
**isoamyl alcohol**	Pseudomonas	aeruginosa	10.1186/1471-2180-12-113
**isoamyl alcohol**	Pseudomonas	aeruginosa	10.3389/fmicb.2021.693075
**isoamyl alcohol**	Pseudomonas	aeruginosa	10.3389/fmicb.2021.693075
**isoamyl alcohol**	Pseudomonas	aeruginosa	10.3389/fmicb.2021.693075
**isoamyl alcohol**	Pseudomonas	aeruginosa	10.3389/fmicb.2021.693075
**isoamyl alcohol**	Pseudomonas	aeruginosa	10.3389/fmicb.2021.693075
**isoamyl alcohol**	Pseudomonas	aeruginosa	10.3389/fmicb.2021.693075
**isoamyl alcohol**	Streptococcus	agalactiae	10.1007/s00253-012-3924-4
**isoamyl alcohol**	Candida	albicans	10.1038/srep27441
**isoamyl alcohol**	Candida	albicans	10.1111/j.1439-0507.2011.02037.x
**isoamyl alcohol**	Candida	albicans	10.1111/myc.12442
**isoamyl alcohol**	Candida	albicans	10.1128/EC.00252-07
**isoamyl alcohol**	Candida	albicans	10.3390/metabo12050432
**isoamyl alcohol**	Candida	albicans	10.3390/metabo12050432
**isoamyl alcohol**	Staphylococcus	aureus	10.1007/s00253-012-3924-4
**isoamyl alcohol**	Staphylococcus	aureus	10.1016/S0378-4347(00)80760-4
**isoamyl alcohol**	Staphylococcus	aureus	10.1038/s41598-020-74909-w
**isoamyl alcohol**	Staphylococcus	aureus	10.1038/s41598-020-74909-w
**isoamyl alcohol**	Staphylococcus	aureus	10.1093/chromsci/bmt042
**isoamyl alcohol**	Staphylococcus	aureus	10.1093/chromsci/bmt042
**isoamyl alcohol**	Staphylococcus	aureus	10.1093/chromsci/bmt042
**isoamyl alcohol**	Staphylococcus	aureus	10.1109/JSEN.2009.2035671
**isoamyl alcohol**	Staphylococcus	aureus	10.1109/JSEN.2009.2035671
**isoamyl alcohol**	Staphylococcus	aureus	10.1128/JCM.00392-10
**isoamyl alcohol**	Staphylococcus	aureus	10.1186/1471-2180-12-113
**isoamyl alcohol**	Staphylococcus	aureus	10.3389/fmicb.2021.693075
**isoamyl alcohol**	Staphylococcus	aureus	10.3389/fmicb.2021.693075
**isoamyl alcohol**	Staphylococcus	aureus	10.3389/fmicb.2021.693075
**isoamyl alcohol**	Staphylococcus	aureus	10.3389/fmicb.2021.693075
**isoamyl alcohol**	Staphylococcus	aureus	10.3389/fmicb.2021.693075
**isoamyl alcohol**	Staphylococcus	aureus	10.3389/fmicb.2021.693075
**isoamyl alcohol**	Staphylococcus	aureus	10.3390/metabo10090347
**isoamyl alcohol**	Staphylococcus	aureus	10.3390/metabo10090347
**isoamyl alcohol**	Staphylococcus	aureus	10.3390/metabo10090347
**isoamyl alcohol**	Staphylococcus	aureus	10.3390/metabo10090347
**isoamyl alcohol**	Mycobacterium	bovis	10.1111/j.1574-6968.2011.02493.x
**isoamyl alcohol**	Penicillium	chrysogenum	10.1038/srep27441

**isoamyl alcohol**	Enterobacter	cloacae	10.1007/s00253-012-3924-4
**isoamyl alcohol**	Enterobacter	cloacae	10.1007/s11306-018-1357-5
**isoamyl alcohol**	Enterobacter	cloacae	10.1016/S0045-6535(97)00209-9
**isoamyl alcohol**	Escherichia	coli	10.1038/s41598-020-74909-w
**isoamyl alcohol**	Escherichia	coli	10.1038/s41598-020-74909-w
**isoamyl alcohol**	Escherichia	coli	10.1093/chromsci/bmt042
**isoamyl alcohol**	Escherichia	coli	10.1093/chromsci/bmt042
**isoamyl alcohol**	Escherichia	coli	10.1093/chromsci/bmt042
**isoamyl alcohol**	Escherichia	coli	10.1111/jam.15716
**isoamyl alcohol**	Escherichia	coli	10.1111/jam.15716
**isoamyl alcohol**	Escherichia	coli	10.3389/fmicb.2021.693075
**isoamyl alcohol**	Escherichia	coli	10.3389/fmicb.2021.693075
**isoamyl alcohol**	Escherichia	coli	10.3389/fmicb.2021.693075
**isoamyl alcohol**	Escherichia	coli	10.3389/fmicb.2021.693075
**isoamyl alcohol**	Escherichia	coli	10.3389/fmicb.2021.693075
**isoamyl alcohol**	Escherichia	coli	10.3389/fmicb.2021.693075
**isoamyl alcohol**	Staphylococcus	epidermidis	10.1038/s41598-020-74909-w
**isoamyl alcohol**	Staphylococcus	epidermidis	10.1038/s41598-020-74909-w
**isoamyl alcohol**	Staphylococcus	epidermidis	10.3390/metabo10090347
**isoamyl alcohol**	Staphylococcus	epidermidis	10.3390/metabo10090347
**isoamyl alcohol**	Staphylococcus	epidermidis	10.3390/metabo10090347
**isoamyl alcohol**	Staphylococcus	epidermidis	10.3390/metabo10090347
**isoamyl alcohol**	Pseudomonas	fluorescens	10.1128/JCM.12.4.521-526.1980
**isoamyl alcohol**	Aspergillus	fumigatus	10.1128/EC.00074-14
**isoamyl alcohol**	Stenotrophomonas	maltophilia	10.1088/1752-7155/9/2/027104
**isoamyl alcohol**	Stenotrophomonas	maltophilia	10.1128/JCM.12.4.521-526.1980
**isoamyl alcohol**	Aspergillus	niger	10.1038/srep27441
**isoamyl alcohol**	Klebsiella	pneumoniae	10.1007/s00253-012-3924-4
**isoamyl alcohol**	Klebsiella	pneumoniae	10.1016/S0378-4347(00)80760-4
**isoamyl alcohol**	Klebsiella	pneumoniae	10.1016/S0378-4347(00)80760-4
**isoamyl alcohol**	Klebsiella	pneumoniae	10.1088/1752-7163/aa8efc
**isoamyl alcohol**	Klebsiella	pneumoniae	10.1111/jam.13372
**isoamyl alcohol**	Streptococcus	pneumoniae	10.1007/s00253-012-3924-4
**isoamyl alcohol**	Pseudomonas	putida	10.1128/JCM.12.4.521-526.1980
**isoamyl alcohol**	Shewanella	putrefaciens	10.1128/JCM.12.4.521-526.1980
**isoamyl alcohol**	Stenotrophomonas	rhizophila	10.1088/1752-7155/9/2/027104
**isoamyl benzoate**	Proteus	mirabilis	10.1007/s00253-012-3924-4
**isoamyl butyrate**	Pseudomonas	aeruginosa	10.1186/1471-2180-12-113
**isoamyl butyrate**	Klebsiella	pneumoniae	10.1111/jam.13372
**isoamyl isobutyrate**	Proteus	mirabilis	10.1007/s00253-012-3924-4
**isoamyl isovalerate**	Pseudomonas	aeruginosa	10.1186/1471-2180-12-113
**isoamyl isovalerate**	Acinetobacter	baumannii	10.1088/1752-7155/10/2/027102
**isoaromadendrene epoxide**	Aspergillus	fumigatus	10.1128/EC.00074-14

**isobutane**	Streptococcus	pneumoniae	10.1099/mic.0.062687-0
**isobutyl acetate**	Pseudomonas	aeruginosa	10.3389/fmicb.2021.693075
**isobutyl acetate**	Pseudomonas	aeruginosa	10.3389/fmicb.2021.693075
**isobutyl acetate**	Candida	albicans	10.1111/myc.12442
**isobutyl acetate**	Escherichia	coli	10.3389/fmicb.2021.693075
**isobutyl acetate**	Escherichia	coli	10.3389/fmicb.2021.693075
**isobutyl acetate**	Escherichia	coli	10.3389/fmicb.2021.693075
**isobutyl acetate**	Escherichia	coli	10.3389/fmicb.2021.693075
**isobutyl formate**	Klebsiella	pneumoniae	10.1111/jam.13372
**isobutyl propionate**	Klebsiella	pneumoniae	10.1111/jam.13372
**isobutylene**	Streptococcus	pneumoniae	10.1099/mic.0.062687-0
**isobutyraldehyde**	Staphylococcus	aureus	10.1016/j.mimet.2010.12.001
**isobutyraldehyde**	Staphylococcus	aureus	10.1186/1471-2180-12-113
**isobutyraldehyde**	Escherichia	coli	10.3390/antibiotics9110797
**isobutyraldehyde**	Escherichia	coli	10.3390/antibiotics9110797
**isobutyraldehyde**	Staphylococcus	epidermidis	10.3390/metabo10090347
**isobutyraldehyde**	Streptococcus	pneumoniae	10.1016/j.jchromb.2018.08.032
**isobutyraldehyde**	Streptococcus	pneumoniae	10.1099/mic.0.062687-0
**isobutyric acid**	Staphylococcus	aureus	10.1016/j.jchromb.2009.05.028
**isobutyric acid**	Staphylococcus	aureus	10.1038/s41598-020-74909-w
**isobutyric acid**	Staphylococcus	aureus	10.1038/s41598-020-74909-w
**isobutyric acid**	Staphylococcus	aureus	10.3389/fmicb.2021.693075
**isobutyric acid**	Staphylococcus	aureus	10.3389/fmicb.2021.693075
**isobutyric acid**	Staphylococcus	aureus	10.3389/fmicb.2021.693075
**isobutyric acid**	Mycobacterium	bovis	10.1371/journal.pone.0194348
**isobutyric acid**	Staphylococcus	epidermidis	10.1038/s41598-020-74909-w
**isobutyric acid**	Staphylococcus	epidermidis	10.1038/s41598-020-74909-w
**isobutyric acid**	Klebsiella	pneumoniae	10.1111/jam.13372
**isobutyric acid**	Streptococcus	pneumoniae	10.3390/metabo11110773
**isobutyronitrile**	Mycobacterium	tuberculosis	10.5588/ijtld.11.0576
**isolongifolene, 4,5,9,10-dehydro-**	Aspergillus	fumigatus	10.1128/EC.00074-14
**isolongifolene, 4,5,9,10-dehydro-**	Aspergillus	fumigatus	10.1128/EC.00074-14
**isoprene**	Pseudomonas	aeruginosa	10.1007/BF00294190
**isoprene**	Pseudomonas	aeruginosa	10.1007/BF00294190
**isoprene**	Pseudomonas	aeruginosa	10.1007/BF00294190
**isoprene**	Pseudomonas	aeruginosa	10.1007/BF00294190
**isoprene**	Pseudomonas	aeruginosa	10.1016/j.mimet.2010.12.001
**isoprene**	Pseudomonas	aeruginosa	10.1016/j.mimet.2010.12.001
**isoprene**	Pseudomonas	aeruginosa	10.1016/S0045-6535(97)00209-9
**isoprene**	Pseudomonas	aeruginosa	10.1088/1752-7155/10/3/037102
**isoprene**	Pseudomonas	aeruginosa	10.1088/1752-7155/10/3/037102
**isoprene**	Pseudomonas	aeruginosa	10.1088/1752-7155/10/3/037102
**isoprene**	Pseudomonas	aeruginosa	10.1088/1752-7155/8/2/027106

**isoprene**	Pseudomonas	aeruginosa	10.1088/1752-7155/8/2/027106
**isoprene**	Pseudomonas	aeruginosa	10.1186/1471-2180-12-113
**isoprene**	Staphylococcus	aureus	10.1088/1752-7155/10/3/037102
**isoprene**	Staphylococcus	aureus	10.1088/1752-7155/10/3/037102
**isoprene**	Staphylococcus	aureus	10.1088/1752-7155/10/3/037102
**isoprene**	Burkholderia	cepacia	10.1088/1752-7155/10/3/037102
**isoprene**	Burkholderia	cepacia	10.1088/1752-7155/10/3/037102
**isoprene**	Burkholderia	cepacia	10.1088/1752-7155/10/3/037102
**isoprene**	Enterobacter	cloacae	10.1016/S0045-6535(97)00209-9
**isoprene**	Escherichia	coli	10.1007/BF00294190
**isoprene**	Escherichia	coli	10.1007/BF00294190
**isoprene**	Escherichia	coli	10.1007/BF00294190
**isoprene**	Escherichia	coli	10.1007/BF00294190
**isoprene**	Escherichia	coli	10.1016/j.mimet.2010.12.001
**isoprene**	Escherichia	coli	10.1016/j.mimet.2010.12.001
**isoprene**	Escherichia	coli	10.1088/1752-7155/8/2/027106
**isoprene**	Escherichia	coli	10.1088/1752-7155/8/2/027106
**isoprene**	Enterococcus	faecalis	10.1016/j.mimet.2010.12.001
**isoprene**	Pseudomonas	fluorescens	10.1016/S0045-6535(97)00209-9
**isoprene**	Aspergillus	fumigatus	10.1128/EC.00074-14
**isoprene**	Haemophilus	influenzae	10.1099/mic.0.062687-0
**isoprene**	Serratia	liquefaciens	10.1016/S0045-6535(97)00209-9
**isoprene**	Stenotrophomonas	maltophilia	10.1088/1752-7155/10/3/037102
**isoprene**	Stenotrophomonas	maltophilia	10.1088/1752-7155/10/3/037102
**isoprene**	Stenotrophomonas	maltophilia	10.1088/1752-7155/10/3/037102
**isoprene**	Proteus	mirabilis	10.1016/j.mimet.2010.12.001
**isoprene**	Proteus	mirabilis	10.1016/j.mimet.2010.12.001
**isoprene**	Klebsiella	pneumoniae	10.1088/1752-7155/8/2/027106
**isoprene**	Pseudomonas	putida	10.1016/S0045-6535(97)00209-9
**isopropenyl ethyl ketone**	Klebsiella	pneumoniae	10.1111/jam.13372
**isopropenylpyrazine**	Aspergillus	fumigatus	10.1088/1752-7155/10/1/016002
**isopropylamine**	Mycobacterium	tuberculosis	10.5588/ijtld.11.0576
**isopropylcyclopropane**	Staphylococcus	epidermidis	10.3390/metabo10090347
**isopropylcyclopropane**	Staphylococcus	epidermidis	10.3390/metabo10090347
**isovaleric acid**	Staphylococcus	aureus	10.1002/jobm.201600505
**isovaleric acid**	Staphylococcus	aureus	10.1016/j.jchromb.2009.05.028
**isovaleric acid**	Staphylococcus	aureus	10.1038/s41598-020-74909-w
**isovaleric acid**	Staphylococcus	aureus	10.1038/s41598-020-74909-w
**isovaleric acid**	Staphylococcus	aureus	10.1093/chromsci/bmt042
**isovaleric acid**	Staphylococcus	aureus	10.1093/chromsci/bmt042
**isovaleric acid**	Staphylococcus	aureus	10.1093/chromsci/bmt042
**isovaleric acid**	Staphylococcus	aureus	10.1186/1471-2180-12-113
**isovaleric acid**	Staphylococcus	aureus	10.3389/fmicb.2021.693075

**isovaleric acid**	Staphylococcus	aureus	10.3389/fmicb.2021.693075
**isovaleric acid**	Staphylococcus	aureus	10.3389/fmicb.2021.693075
**isovaleric acid**	Staphylococcus	aureus	10.3389/fmicb.2021.693075
**isovaleric acid**	Staphylococcus	aureus	10.3389/fmicb.2021.693075
**isovaleric acid**	Staphylococcus	aureus	10.3389/fmicb.2021.693075
**isovaleric acid**	Staphylococcus	aureus	10.3390/metabo10090347
**isovaleric acid**	Staphylococcus	aureus	10.3390/metabo10090347
**isovaleric acid**	Escherichia	coli	10.1111/jam.15716
**isovaleric acid**	Escherichia	coli	10.1111/jam.15716
**isovaleric acid**	Staphylococcus	epidermidis	10.1038/s41598-020-74909-w
**isovaleric acid**	Staphylococcus	epidermidis	10.1038/s41598-020-74909-w
**isovaleric acid**	Staphylococcus	epidermidis	10.3390/metabo10090347
**isovaleric acid**	Staphylococcus	epidermidis	10.3390/metabo10090347
**isovaleric acid**	Staphylococcus	epidermidis	10.3390/metabo10090347
**isovaleric acid**	Klebsiella	pneumoniae	10.1111/jam.13372
**isovaleronitrile**	Staphylococcus	aureus	10.3390/metabo10090347
**isovaleronitrile**	Staphylococcus	epidermidis	10.3390/metabo10090347
**isovaleronitrile**	Staphylococcus	epidermidis	10.3390/metabo10090347
**Ledene oxide(II)**	Aspergillus	fumigatus	10.1128/EC.00074-14
**lepidine**	Escherichia	coli	10.1111/jam.15716
**lepidine**	Escherichia	coli	10.1111/jam.15716
**limonene**	Staphylococcus	epidermidis	10.3390/metabo10090347
**limonene**	Aspergillus	fumigatus	10.1039/c8an00841h
**limonene**	Aspergillus	fumigatus	10.1039/c8an00841h
**limonene**	Aspergillus	fumigatus	10.1093/cid/ciu725
**limonene**	Aspergillus	fumigatus	10.1093/cid/ciu725
**linalool**	Aspergillus	fumigatus	10.1128/EC.00074-14
**longifolene**	Acinetobacter	baumannii	10.1088/1752-7155/10/2/027102
**maleimide**	Candida	albicans	10.3390/metabo12050432
**malondialdehyde**	Pseudomonas	aeruginosa	10.1088/1752-7155/10/3/037102
**malondialdehyde**	Pseudomonas	aeruginosa	10.1088/1752-7155/10/3/037102
**malondialdehyde**	Pseudomonas	aeruginosa	10.1088/1752-7155/10/3/037102
**malondialdehyde**	Staphylococcus	aureus	10.1088/1752-7155/10/3/037102
**malondialdehyde**	Staphylococcus	aureus	10.1088/1752-7155/10/3/037102
**malondialdehyde**	Staphylococcus	aureus	10.1088/1752-7155/10/3/037102
**malondialdehyde**	Burkholderia	cepacia	10.1088/1752-7155/10/3/037102
**malondialdehyde**	Burkholderia	cepacia	10.1088/1752-7155/10/3/037102
**malondialdehyde**	Burkholderia	cepacia	10.1088/1752-7155/10/3/037102
**malondialdehyde**	Stenotrophomonas	maltophilia	10.1088/1752-7155/10/3/037102
**malondialdehyde**	Stenotrophomonas	maltophilia	10.1088/1752-7155/10/3/037102
**malondialdehyde**	Stenotrophomonas	maltophilia	10.1088/1752-7155/10/3/037102
**m-cresol**	Escherichia	coli	10.3390/antibiotics9110797
**mesityl oxide**	Pseudomonas	aeruginosa	10.1038/s41598-020-74909-w

**mesityl oxide**	Pseudomonas	aeruginosa	10.1128/mSphere.00843-20
**mesityl oxide**	Staphylococcus	aureus	10.1038/s41598-020-74909-w
**mesityl oxide**	Escherichia	coli	10.1038/s41598-020-74909-w
**mesityl oxide**	Staphylococcus	epidermidis	10.1038/s41598-020-74909-w
**mesityl oxide**	Klebsiella	pneumoniae	10.1111/jam.13372
**mesitylene**	Candida	albicans	10.1038/srep27441
**mesitylene**	Penicillium	chrysogenum	10.1038/srep27441
**mesitylene**	Aspergillus	niger	10.1038/srep27441
**methacrolein**	Streptococcus	pneumoniae	10.1099/mic.0.062687-0
**methane**	Pseudomonas	aeruginosa	10.1088/1752-7155/10/3/037102
**methane**	Pseudomonas	aeruginosa	10.1088/1752-7155/10/3/037102
**methane**	Pseudomonas	aeruginosa	10.1088/1752-7155/10/3/037102
**methane**	Staphylococcus	aureus	10.1088/1752-7155/10/3/037102
**methane**	Staphylococcus	aureus	10.1088/1752-7155/10/3/037102
**methane**	Staphylococcus	aureus	10.1088/1752-7155/10/3/037102
**methane**	Burkholderia	cepacia	10.1088/1752-7155/10/3/037102
**methane**	Burkholderia	cepacia	10.1088/1752-7155/10/3/037102
**methane**	Burkholderia	cepacia	10.1088/1752-7155/10/3/037102
**methane**	Stenotrophomonas	maltophilia	10.1088/1752-7155/10/3/037102
**methane**	Stenotrophomonas	maltophilia	10.1088/1752-7155/10/3/037102
**methane**	Stenotrophomonas	maltophilia	10.1088/1752-7155/10/3/037102
**methanethiol**	Pseudomonas	aeruginosa	10.1002/ppul.20170
**methanethiol**	Pseudomonas	aeruginosa	10.1002/ppul.20170
**methanethiol**	Pseudomonas	aeruginosa	10.1016/j.mimet.2005.09.003
**methanethiol**	Pseudomonas	aeruginosa	10.1016/j.mimet.2010.12.001
**methanethiol**	Pseudomonas	aeruginosa	10.1016/j.mimet.2010.12.001
**methanethiol**	Pseudomonas	aeruginosa	10.1088/1752-7155/10/3/037102
**methanethiol**	Pseudomonas	aeruginosa	10.1088/1752-7155/10/3/037102
**methanethiol**	Pseudomonas	aeruginosa	10.1088/1752-7155/10/3/037102
**methanethiol**	Pseudomonas	aeruginosa	10.1111/j.1365-2672.2012.05414.[illegible]
**methanethiol**	Pseudomonas	aeruginosa	10.1128/JCM.12.4.521-526.1980
**methanethiol**	Pseudomonas	aeruginosa	10.1128/JCM.12.4.521-526.1980
**methanethiol**	Pseudomonas	aeruginosa	10.1128/JCM.12.4.521-526.1980
**methanethiol**	Pseudomonas	aeruginosa	10.1128/JCM.12.4.521-526.1980
**methanethiol**	Pseudomonas	aeruginosa	10.1128/JCM.12.4.521-526.1980
**methanethiol**	Pseudomonas	aeruginosa	10.1128/JCM.12.4.521-526.1980
**methanethiol**	Pseudomonas	aeruginosa	10.1128/JCM.12.4.521-526.1980
**methanethiol**	Pseudomonas	aeruginosa	10.1128/JCM.12.4.521-526.1980
**methanethiol**	Pseudomonas	aeruginosa	10.1128/JCM.12.4.521-526.1980
**methanethiol**	Pseudomonas	aeruginosa	10.1128/JCM.12.4.521-526.1980
**methanethiol**	Pseudomonas	aeruginosa	10.1128/JCM.12.4.521-526.1980
**methanethiol**	Pseudomonas	aeruginosa	10.1186/1471-2180-12-113
**methanethiol**	Staphylococcus	aureus	10.1016/j.diagmicrobio.2006.01.0[illegible]

**methanethiol**	Staphylococcus	aureus	10.1016/j.mimet.2005.09.003
**methanethiol**	Staphylococcus	aureus	10.1016/j.mimet.2010.12.001
**methanethiol**	Staphylococcus	aureus	10.1016/j.mimet.2010.12.001
**methanethiol**	Staphylococcus	aureus	10.1088/1752-7155/10/3/037102
**methanethiol**	Staphylococcus	aureus	10.1088/1752-7155/10/3/037102
**methanethiol**	Staphylococcus	aureus	10.1088/1752-7155/10/3/037102
**methanethiol**	Staphylococcus	aureus	10.1186/1471-2180-12-113
**methanethiol**	Burkholderia	cepacia	10.1016/j.mimet.2010.12.001
**methanethiol**	Burkholderia	cepacia	10.1088/1752-7155/10/3/037102
**methanethiol**	Burkholderia	cepacia	10.1088/1752-7155/10/3/037102
**methanethiol**	Burkholderia	cepacia	10.1088/1752-7155/10/3/037102
**methanethiol**	Escherichia	coli	10.1016/j.diagmicrobio.2006.01.0[illegible]
**methanethiol**	Escherichia	coli	10.1016/j.mimet.2005.09.003
**methanethiol**	Escherichia	coli	10.1016/j.mimet.2005.09.016
**methanethiol**	Escherichia	coli	10.1016/j.mimet.2010.12.001
**methanethiol**	Escherichia	coli	10.1016/j.mimet.2010.12.001
**methanethiol**	Escherichia	coli	10.1111/j.1365-2672.2012.05414.[illegible]
**methanethiol**	Escherichia	coli	10.1128/AEM.02069-07
**methanethiol**	Enterococcus	faecalis	10.1016/j.mimet.2010.12.001
**methanethiol**	Aspergillus	fumigatus	10.1039/C4AY01217H
**methanethiol**	Haemophilus	influenzae	10.1099/mic.0.062687-0
**methanethiol**	Stenotrophomonas	maltophilia	10.1088/1752-7155/10/3/037102
**methanethiol**	Stenotrophomonas	maltophilia	10.1088/1752-7155/10/3/037102
**methanethiol**	Stenotrophomonas	maltophilia	10.1088/1752-7155/10/3/037102
**methanethiol**	Neisseria	meningitidis	10.1016/j.mimet.2005.09.016
**methanethiol**	Proteus	mirabilis	10.1016/j.mimet.2010.12.001
**methanethiol**	Proteus	mirabilis	10.1016/j.mimet.2010.12.001
**methanethiol**	Klebsiella	pneumoniae	10.1111/jam.13372
**methanethiol**	Streptococcus	pneumoniae	10.1016/j.diagmicrobio.2006.01.0[illegible]
**methanethiol**	Streptococcus	pneumoniae	10.1016/j.jchromb.2009.05.028
**methanethiol**	Streptococcus	pneumoniae	10.1016/j.mimet.2005.09.016
**methanethiol**	Streptococcus	pneumoniae	10.1099/mic.0.062687-0
**methanethiol**	Pseudomonas	putida	10.1016/S0045-6535(97)00209-9
**methanethiol**	Streptococcus	pyogenes	10.1016/j.mimet.2010.12.001
**methanol**	Pseudomonas	aeruginosa	10.1016/j.jchromb.2012.05.038
**methanol**	Pseudomonas	aeruginosa	10.1088/1752-7155/10/3/037102
**methanol**	Pseudomonas	aeruginosa	10.1088/1752-7155/10/3/037102
**methanol**	Pseudomonas	aeruginosa	10.1088/1752-7155/10/3/037102
**methanol**	Staphylococcus	aureus	10.1088/1752-7155/10/3/037102
**methanol**	Staphylococcus	aureus	10.1088/1752-7155/10/3/037102
**methanol**	Staphylococcus	aureus	10.1088/1752-7155/10/3/037102
**methanol**	Mycobacterium	bovis	10.1111/j.1574-6968.2011.02493.[illegible]
**methanol**	Burkholderia	cepacia	10.1088/1752-7155/10/3/037102

**methanol**	Burkholderia	cepacia	10.1088/1752-7155/10/3/037102
**methanol**	Burkholderia	cepacia	10.1088/1752-7155/10/3/037102
**methanol**	Escherichia	coli	10.1128/AEM.02069-07
**methanol**	Aspergillus	fumigatus	10.1039/C4AY01217H
**methanol**	Haemophilus	influenzae	10.1099/mic.0.062687-0
**methanol**	Stenotrophomonas	maltophilia	10.1088/1752-7155/10/3/037102
**methanol**	Stenotrophomonas	maltophilia	10.1088/1752-7155/10/3/037102
**methanol**	Stenotrophomonas	maltophilia	10.1088/1752-7155/10/3/037102
**methenamine**	Escherichia	coli	10.1002/jssc.201800684
**methionol**	Candida	albicans	10.3390/metabo12050432
**methionol**	Candida	albicans	10.3390/metabo12050432
**methyl (2s)-2-methylbutanoate**	Pseudomonas	aeruginosa	10.1128/mSphere.00843-20
**methyl 2-ethylhexanoate**	Pseudomonas	aeruginosa	10.1007/s11306-018-1357-5
**methyl 2-ethylhexanoate**	Aspergillus	fumigatus	10.1088/1752-7155/6/1/016002
**methyl 2-ethylhexanoate**	Aspergillus	fumigatus	10.1088/1752-7155/6/1/016002
**methyl 2-ethylhexanoate**	Aspergillus	fumigatus	10.1088/1752-7155/6/1/016002
**methyl 2-ethylhexanoate**	Aspergillus	fumigatus	10.1088/1752-7155/6/1/016002
**methyl 2-ethylhexanoate**	Aspergillus	fumigatus	10.1088/1752-7155/6/1/016002
**methyl 2-ethylhexanoate**	Aspergillus	fumigatus	10.1088/1752-7155/6/1/016002
**methyl 2-furoate**	Mycobacterium	tuberculosis	10.3762/bjoc.8.31
**methyl 2-methylbutyrate**	Pseudomonas	aeruginosa	10.1186/1471-2180-12-113
**methyl 2-methylbutyrate**	Pseudomonas	aeruginosa	10.3389/fmicb.2021.693075
**methyl 2-methylbutyrate**	Pseudomonas	aeruginosa	10.3389/fmicb.2021.693075
**methyl 2-methylbutyrate**	Pseudomonas	aeruginosa	10.3389/fmicb.2021.693075
**methyl 2-methylbutyrate**	Mycobacterium	bovis	10.1111/j.1574-6968.2011.02493.[illegible]
**methyl 2-methylhexanoate**	Mycobacterium	avium	10.1088/1752-7163/aa6e06
**methyl 2-methylhexanoate**	Mycobacterium	bovis	10.1088/1752-7163/aa6e06
**methyl 2-methylhexanoate**	Mycobacterium	intracellulare	10.1088/1752-7163/aa6e06
**methyl 2-methylhexanoate**	Mycobacterium	xenopi	10.1088/1752-7163/aa6e06
**methyl 4-methoxybenzoate**	Mycobacterium	bovis	10.1016/j.tube.2008.01.002
**methyl 4-methoxybenzoate**	Mycobacterium	bovis	10.1016/j.tube.2008.01.002
**methyl 4-methoxybenzoate**	Mycobacterium	bovis	10.1016/j.tube.2008.01.002
**methyl 4-methoxybenzoate**	Mycobacterium	bovis	10.1016/j.tube.2008.01.002
**methyl 4-methoxybenzoate**	Mycobacterium	bovis	10.1016/j.tube.2008.01.002
**methyl 4-methoxybenzoate**	Mycobacterium	bovis	10.1016/j.tube.2008.01.002
**methyl 4-methoxybenzoate**	Mycobacterium	tuberculosis	10.1016/j.tube.2008.01.002
**methyl 4-methoxybenzoate**	Mycobacterium	tuberculosis	10.1016/j.tube.2008.01.002
**methyl 4-methoxybenzoate**	Mycobacterium	tuberculosis	10.1016/j.tube.2008.01.002
**methyl 4-methoxybenzoate**	Mycobacterium	tuberculosis	10.1016/j.tube.2008.01.002
**methyl 4-methoxybenzoate**	Mycobacterium	tuberculosis	10.1016/j.tube.2008.01.002
**methyl 4-methoxybenzoate**	Mycobacterium	tuberculosis	10.1016/j.tube.2008.01.002
**methyl benzoate**	Pseudomonas	aeruginosa	10.3389/fmicb.2021.693075
**methyl benzoate**	Pseudomonas	aeruginosa	10.3389/fmicb.2021.693075

**methyl benzoate**	Enterobacter	cloacae	10.1007/s00253-012-3924-4
**methyl benzoate**	Escherichia	coli	10.3389/fmicb.2021.693075
**methyl benzoate**	Escherichia	coli	10.3389/fmicb.2021.693075
**methyl benzoate**	Escherichia	coli	10.3389/fmicb.2021.693075
**methyl benzoate**	Escherichia	coli	10.3389/fmicb.2021.693075
**methyl benzoate**	Escherichia	coli	10.3389/fmicb.2021.693075
**methyl benzoate**	Escherichia	coli	10.3389/fmicb.2021.693075
**methyl benzoate**	Staphylococcus	epidermidis	10.1038/s41598-020-74909-w
**methyl benzoate**	Staphylococcus	epidermidis	10.3390/metabo10090347
**methyl benzoate**	Mycobacterium	tuberculosis	10.3762/bjoc.8.31
**methyl butyrate**	Pseudomonas	aeruginosa	10.1128/mSphere.00843-20
**methyl isovalerate**	Pseudomonas	aeruginosa	10.1128/mSphere.00843-20
**methyl isovalerate**	Pseudomonas	aeruginosa	10.3389/fmicb.2021.693075
**methyl isovalerate**	Pseudomonas	aeruginosa	10.3389/fmicb.2021.693075
**methyl isovalerate**	Pseudomonas	aeruginosa	10.3389/fmicb.2021.693075
**methyl isovalerate**	Pseudomonas	aeruginosa	10.3389/fmicb.2021.693075
**methyl isovalerate**	Staphylococcus	aureus	10.3389/fmicb.2021.693075
**methyl laurate**	Escherichia	coli	10.1038/s41598-020-74909-w
**methyl laurate**	Escherichia	coli	10.1038/s41598-020-74909-w
**methyl methacrylate**	Pseudomonas	aeruginosa	10.1186/1471-2180-12-113
**methyl methacrylate**	Candida	albicans	10.1038/srep27441
**methyl methacrylate**	Staphylococcus	aureus	10.1186/1471-2180-12-113
**methyl methacrylate**	Penicillium	chrysogenum	10.1038/srep27441
**methyl methacrylate**	Haemophilus	influenzae	10.1099/mic.0.062687-0
**methyl methacrylate**	Aspergillus	niger	10.1038/srep27441
**methyl nerate**	Aspergillus	fumigatus	10.1128/EC.00074-14
**methyl nicotinate**	Mycobacterium	bovis	10.1016/j.tube.2008.01.002
**methyl nicotinate**	Mycobacterium	bovis	10.1016/j.tube.2008.01.002
**methyl nicotinate**	Mycobacterium	bovis	10.1016/j.tube.2008.01.002
**methyl nicotinate**	Mycobacterium	bovis	10.1016/j.tube.2008.01.002
**methyl nicotinate**	Mycobacterium	bovis	10.1016/j.tube.2008.01.002
**methyl nicotinate**	Mycobacterium	bovis	10.1016/j.tube.2008.01.002
**methyl nicotinate**	Mycobacterium	tuberculosis	10.1016/j.tube.2008.01.002
**methyl nicotinate**	Mycobacterium	tuberculosis	10.1016/j.tube.2008.01.002
**methyl nicotinate**	Mycobacterium	tuberculosis	10.1016/j.tube.2008.01.002
**methyl nicotinate**	Mycobacterium	tuberculosis	10.1016/j.tube.2008.01.002
**methyl nicotinate**	Mycobacterium	tuberculosis	10.1016/j.tube.2008.01.002
**methyl nicotinate**	Mycobacterium	tuberculosis	10.1016/j.tube.2008.01.002
**methyl nitrite**	Klebsiella	pneumoniae	10.1088/1752-7155/10/2/027101
**methyl palmitate**	Escherichia	coli	10.1038/s41598-020-74909-w
**methyl phenylacetate**	Mycobacterium	avium	10.1016/j.tube.2008.01.002
**methyl phenylacetate**	Mycobacterium	avium	10.1016/j.tube.2008.01.002
**methyl phenylacetate**	Mycobacterium	bovis	10.1016/j.tube.2008.01.002

**methyl phenylacetate**	Mycobacterium	bovis	10.1016/j.tube.2008.01.002
**methyl phenylacetate**	Mycobacterium	bovis	10.1016/j.tube.2008.01.002
**methyl phenylacetate**	Mycobacterium	bovis	10.1016/j.tube.2008.01.002
**methyl phenylacetate**	Mycobacterium	bovis	10.1016/j.tube.2008.01.002
**methyl phenylacetate**	Mycobacterium	bovis	10.1016/j.tube.2008.01.002
**methyl phenylacetate**	Mycobacterium	tuberculosis	10.1016/j.tube.2008.01.002
**methyl phenylacetate**	Mycobacterium	tuberculosis	10.1016/j.tube.2008.01.002
**methyl phenylacetate**	Mycobacterium	tuberculosis	10.1016/j.tube.2008.01.002
**methyl phenylacetate**	Mycobacterium	tuberculosis	10.1016/j.tube.2008.01.002
**methyl phenylacetate**	Mycobacterium	tuberculosis	10.1016/j.tube.2008.01.002
**methyl phenylacetate**	Mycobacterium	tuberculosis	10.1016/j.tube.2008.01.002
**methyl propionate**	Haemophilus	influenzae	10.1099/mic.0.062687-0
**methyl tetradecanoate**	Escherichia	coli	10.1038/s41598-020-74909-w
**methyl tetradecanoate**	Escherichia	coli	10.1038/s41598-020-74909-w
**methyl tetradecanoate**	Escherichia	coli	10.3389/fmicb.2021.693075
**methyl tetradecanoate**	Escherichia	coli	10.3389/fmicb.2021.693075
**methyl tetradecanoate**	Escherichia	coli	10.3389/fmicb.2021.693075
**methyl tetradecanoate**	Escherichia	coli	10.3389/fmicb.2021.693075
**methyl thioacetate**	Pseudomonas	aeruginosa	10.1128/mSphere.00843-20
**methyl thiocyanate**	Pseudomonas	aeruginosa	10.1002/rcm.5146
**methyl thiocyanate**	Pseudomonas	aeruginosa	10.1002/rcm.5146
**methyl thiocyanate**	Pseudomonas	aeruginosa	10.1002/rcm.5146
**methyl thiocyanate**	Pseudomonas	aeruginosa	10.1002/rcm.5146
**methyl thiocyanate**	Pseudomonas	aeruginosa	10.1002/rcm.5146
**methyl thiocyanate**	Pseudomonas	aeruginosa	10.1016/j.jchromb.2012.05.038
**methyl thiocyanate**	Pseudomonas	aeruginosa	10.1088/1752-7155/10/3/037102
**methyl thiocyanate**	Pseudomonas	aeruginosa	10.1088/1752-7155/10/3/037102
**methyl thiocyanate**	Pseudomonas	aeruginosa	10.1088/1752-7155/10/3/037102
**methyl thiocyanate**	Pseudomonas	aeruginosa	10.1088/1752-7163/aa8efc
**methyl thiocyanate**	Pseudomonas	aeruginosa	10.1088/1752-7163/aa8efc
**methyl thiocyanate**	Pseudomonas	aeruginosa	10.1128/mSphere.00843-20
**methyl thiocyanate**	Pseudomonas	aeruginosa	10.3389/fmicb.2021.693075
**methyl thiocyanate**	Pseudomonas	aeruginosa	10.3389/fmicb.2021.693075
**methyl thiocyanate**	Pseudomonas	aeruginosa	10.3389/fmicb.2021.693075
**methyl thiocyanate**	Pseudomonas	aeruginosa	10.3389/fmicb.2021.693075
**methyl thiocyanate**	Pseudomonas	aeruginosa	10.3389/fmicb.2021.693075
**methyl thiocyanate**	Staphylococcus	aureus	10.1088/1752-7155/10/3/037102
**methyl thiocyanate**	Staphylococcus	aureus	10.1088/1752-7155/10/3/037102
**methyl thiocyanate**	Staphylococcus	aureus	10.1088/1752-7155/10/3/037102
**methyl thiocyanate**	Staphylococcus	aureus	10.3389/fmicb.2021.693075
**methyl thiocyanate**	Staphylococcus	aureus	10.3389/fmicb.2021.693075
**methyl thiocyanate**	Staphylococcus	aureus	10.3389/fmicb.2021.693075
**methyl thiocyanate**	Staphylococcus	aureus	10.3389/fmicb.2021.693075

**methyl thiocyanate**	Staphylococcus	aureus	10.3389/fmicb.2021.693075
**methyl thiocyanate**	Staphylococcus	aureus	10.3389/fmicb.2021.693075
**methyl thiocyanate**	Burkholderia	cepacia	10.1088/1752-7155/10/3/037102
**methyl thiocyanate**	Burkholderia	cepacia	10.1088/1752-7155/10/3/037102
**methyl thiocyanate**	Burkholderia	cepacia	10.1088/1752-7155/10/3/037102
**methyl thiocyanate**	Stenotrophomonas	maltophilia	10.1088/1752-7155/10/3/037102
**methyl thiocyanate**	Stenotrophomonas	maltophilia	10.1088/1752-7155/10/3/037102
**methyl thiocyanate**	Stenotrophomonas	maltophilia	10.1088/1752-7155/10/3/037102
**methyl vinyl ketone**	Pseudomonas	aeruginosa	10.1088/1752-7155/10/4/047102
**methyl vinyl ketone**	Staphylococcus	epidermidis	10.3390/metabo10090347
**methyl vinyl ketone**	Staphylococcus	epidermidis	10.3390/metabo10090347
**methylcyclododecane**	Mycobacterium	tuberculosis	10.1016/j.tube.2006.03.004
**methylcyclododecane**	Mycobacterium	tuberculosis	10.1016/j.tube.2006.03.004
**m-xylene**	Candida	albicans	10.1038/srep27441
**m-xylene**	Penicillium	chrysogenum	10.1038/srep27441
**m-xylene**	Aspergillus	niger	10.1038/srep27441
**myrcene**	Staphylococcus	aureus	10.3390/metabo10090347
**myrcene**	Staphylococcus	aureus	10.3390/metabo10090347
**N-(Phenylmethylene)-1-butanamine**	Escherichia	coli	10.1007/s00253-012-3924-4
**N-(Phenylmethylene)-1-butanamine**	Proteus	mirabilis	10.1007/s00253-012-3924-4
**n,n-dimethylthioformamide**	Mycobacterium	tuberculosis	10.5588/ijtld.11.0576
**N1,N2-Dibenzylideneethylenediamine**	Escherichia	coli	10.1007/s00253-012-3924-4
**naphthalene**	Escherichia	coli	10.1111/jam.15716
**n-benzylidenemethylamine**	Escherichia	coli	10.1007/s00253-012-3924-4
**n-butylphthalimide**	Proteus	mirabilis	10.1007/s00253-012-3924-4
**nerolidol**	Candida	albicans	10.1128/EC.00252-07
**nerolidol**	Candida	albicans	10.3390/metabo12050432
**n-ethylmethylamine**	Mycobacterium	tuberculosis	10.5588/ijtld.11.0576
**n-ethylphenylacetamide**	Escherichia	coli	10.3390/metabo11110773
**nicotinic acid**	Pseudomonas	aeruginosa	10.3390/metabo11110773
**nonanal**	Pseudomonas	aeruginosa	10.1007/s00253-013-4762-8
**nonanal**	Pseudomonas	aeruginosa	10.1007/s00253-013-4762-8
**nonanal**	Pseudomonas	aeruginosa	10.1088/1752-7155/10/3/037102
**nonanal**	Pseudomonas	aeruginosa	10.1088/1752-7155/10/3/037102
**nonanal**	Pseudomonas	aeruginosa	10.1088/1752-7155/10/3/037102
**nonanal**	Candida	albicans	10.1038/srep27441
**nonanal**	Staphylococcus	aureus	10.1088/1752-7155/10/3/037102
**nonanal**	Staphylococcus	aureus	10.1088/1752-7155/10/3/037102
**nonanal**	Staphylococcus	aureus	10.1088/1752-7155/10/3/037102
**nonanal**	Burkholderia	cepacia	10.1088/1752-7155/10/3/037102
**nonanal**	Burkholderia	cepacia	10.1088/1752-7155/10/3/037102
**nonanal**	Burkholderia	cepacia	10.1088/1752-7155/10/3/037102
**nonanal**	Penicillium	chrysogenum	10.1038/srep27441

**nonanal**	Escherichia	coli	10.1007/s00253-013-4762-8
**nonanal**	Escherichia	coli	10.1007/s00253-013-4762-8
**nonanal**	Stenotrophomonas	maltophilia	10.1088/1752-7155/10/3/037102
**nonanal**	Stenotrophomonas	maltophilia	10.1088/1752-7155/10/3/037102
**nonanal**	Stenotrophomonas	maltophilia	10.1088/1752-7155/10/3/037102
**nonanal**	Aspergillus	niger	10.1038/srep27441
**nonane**	Mycobacterium	bovis	10.1371/journal.pone.0194348
**n-tert-butylbenzamide**	Proteus	mirabilis	10.1007/s00253-012-3924-4
**octadecane**	Escherichia	coli	10.1111/jam.15716
**octanal**	Pseudomonas	aeruginosa	10.1088/1752-7155/10/3/037102
**octanal**	Pseudomonas	aeruginosa	10.1088/1752-7155/10/3/037102
**octanal**	Pseudomonas	aeruginosa	10.1088/1752-7155/10/3/037102
**octanal**	Staphylococcus	aureus	10.1088/1752-7155/10/3/037102
**octanal**	Staphylococcus	aureus	10.1088/1752-7155/10/3/037102
**octanal**	Staphylococcus	aureus	10.1088/1752-7155/10/3/037102
**octanal**	Burkholderia	cepacia	10.1088/1752-7155/10/3/037102
**octanal**	Burkholderia	cepacia	10.1088/1752-7155/10/3/037102
**octanal**	Burkholderia	cepacia	10.1088/1752-7155/10/3/037102
**octanal**	Stenotrophomonas	maltophilia	10.1088/1752-7155/10/3/037102
**octanal**	Stenotrophomonas	maltophilia	10.1088/1752-7155/10/3/037102
**octanal**	Stenotrophomonas	maltophilia	10.1088/1752-7155/10/3/037102
**octanal**	Klebsiella	pneumoniae	10.1111/jam.13372
**octane**	Mycobacterium	bovis	10.1371/journal.pone.0194348
**octanenitrile**	Pseudomonas	aeruginosa	10.1128/mSphere.00843-20
**octanoic acid**	Streptococcus	pneumoniae	10.3390/metabo11110773
**o-cymene**	Candida	albicans	10.1038/srep27441
**o-cymene**	Penicillium	chrysogenum	10.1038/srep27441
**o-cymene**	Aspergillus	fumigatus	10.1128/EC.00074-14
**o-cymene**	Aspergillus	niger	10.1038/srep27441
**o-xylene**	Candida	albicans	10.1038/srep27441
**o-xylene**	Penicillium	chrysogenum	10.1038/srep27441
**o-xylene**	Aspergillus	niger	10.1038/srep27441
**oxypentadienecarboxylic acid**	Staphylococcus	aureus	10.3390/metabo11110773
**p-cresol**	Pseudomonas	aeruginosa	10.1128/JCM.00392-10
**p-cresol**	Staphylococcus	aureus	10.1128/JCM.00392-10
**p-cresol**	Escherichia	coli	10.1002/jssc.201800684
**p-cresol**	Klebsiella	pneumoniae	10.1111/jam.13372
**p-cymene**	Mycobacterium	tuberculosis	10.1016/j.tube.2006.03.004
**p-cymene**	Mycobacterium	tuberculosis	10.1016/j.tube.2006.03.004
**pent-2-ene**	Pseudomonas	aeruginosa	10.1088/1752-7155/8/2/027106
**pent-2-ene**	Pseudomonas	aeruginosa	10.1088/1752-7155/8/2/027106
**pentadecan-2-one**	Escherichia	coli	10.1002/jssc.201800684
**pentadecan-2-one**	Escherichia	coli	10.1038/s41598-020-74909-w

**pentadecan-2-one**	Escherichia	coli	10.1038/s41598-020-74909-w
**pentadecan-2-one**	Escherichia	coli	10.1111/jam.15716
**pentadecan-2-one**	Escherichia	coli	10.1111/jam.15716
**pentadecan-2-one**	Escherichia	coli	10.3389/fmicb.2021.693075
**pentadecan-2-one**	Escherichia	coli	10.3389/fmicb.2021.693075
**pentadecan-2-one**	Escherichia	coli	10.3389/fmicb.2021.693075
**pentadecan-2-one**	Escherichia	coli	10.3389/fmicb.2021.693075
**pentadecan-2-one**	Escherichia	coli	10.3389/fmicb.2021.693075
**pentadecan-2-one**	Escherichia	coli	10.3389/fmicb.2021.693075
**pentadecane**	Pseudomonas	aeruginosa	10.1038/s41598-020-74909-w
**pentane**	Pseudomonas	aeruginosa	10.1088/1752-7155/10/3/037102
**pentane**	Pseudomonas	aeruginosa	10.1088/1752-7155/10/3/037102
**pentane**	Pseudomonas	aeruginosa	10.1088/1752-7155/10/3/037102
**pentane**	Staphylococcus	aureus	10.1088/1752-7155/10/3/037102
**pentane**	Staphylococcus	aureus	10.1088/1752-7155/10/3/037102
**pentane**	Staphylococcus	aureus	10.1088/1752-7155/10/3/037102
**pentane**	Mycobacterium	bovis	10.1371/journal.pone.0194348
**pentane**	Burkholderia	cepacia	10.1088/1752-7155/10/3/037102
**pentane**	Burkholderia	cepacia	10.1088/1752-7155/10/3/037102
**pentane**	Burkholderia	cepacia	10.1088/1752-7155/10/3/037102
**pentane**	Stenotrophomonas	maltophilia	10.1088/1752-7155/10/3/037102
**pentane**	Stenotrophomonas	maltophilia	10.1088/1752-7155/10/3/037102
**pentane**	Stenotrophomonas	maltophilia	10.1088/1752-7155/10/3/037102
**pentyl propionate**	Candida	albicans	10.3390/metabo12050432
**phenaceturic acid**	Escherichia	coli	10.3390/metabo11110773
**phenethyl acetate**	Streptococcus	agalactiae	10.1007/s00253-012-3924-4
**phenethyl acetate**	Candida	albicans	10.1038/srep27441
**phenethyl acetate**	Penicillium	chrysogenum	10.1038/srep27441
**phenethyl acetate**	Proteus	mirabilis	10.1007/s00253-012-3924-4
**phenethyl acetate**	Aspergillus	niger	10.1038/srep27441
**phenethyl butyrate**	Serratia	marcescens	10.1007/s00253-012-3924-4
**phenethyl butyrate**	Proteus	mirabilis	10.1007/s00253-012-3924-4
**phenol**	Pseudomonas	aeruginosa	10.1038/s41598-020-74909-w
**phenol**	Pseudomonas	aeruginosa	10.1088/1752-7155/10/3/037102
**phenol**	Pseudomonas	aeruginosa	10.1088/1752-7155/10/3/037102
**phenol**	Pseudomonas	aeruginosa	10.1088/1752-7155/10/3/037102
**phenol**	Staphylococcus	aureus	10.1038/s41598-020-74909-w
**phenol**	Staphylococcus	aureus	10.1038/s41598-020-74909-w
**phenol**	Staphylococcus	aureus	10.1088/1752-7155/10/3/037102
**phenol**	Staphylococcus	aureus	10.1088/1752-7155/10/3/037102
**phenol**	Staphylococcus	aureus	10.1088/1752-7155/10/3/037102
**phenol**	Burkholderia	cepacia	10.1088/1752-7155/10/3/037102
**phenol**	Burkholderia	cepacia	10.1088/1752-7155/10/3/037102

**phenol**	Enterobacter	cloacae	10.1007/s00253-012-3924-4
**phenol**	Escherichia	coli	10.1007/s00253-012-3924-4
**phenol**	Escherichia	coli	10.1111/jam.15716
**phenol**	Escherichia	coli	10.1111/jam.15716
**phenol**	Stenotrophomonas	maltophilia	10.1088/1752-7155/10/3/037102
**phenol**	Stenotrophomonas	maltophilia	10.1088/1752-7155/10/3/037102
**phenol**	Stenotrophomonas	maltophilia	10.1088/1752-7155/10/3/037102
**phenol**	Serratia	marcescens	10.1007/s00253-012-3924-4
**phenylacetaldehyde**	Pseudomonas	aeruginosa	10.1007/s00253-013-4762-8
**phenylacetaldehyde**	Pseudomonas	aeruginosa	10.1007/s00253-013-4762-8
**phenylacetaldehyde**	Candida	albicans	10.1038/srep27441
**phenylacetaldehyde**	Penicillium	chrysogenum	10.1038/srep27441
**phenylacetaldehyde**	Escherichia	coli	10.1007/s00253-013-4762-8
**phenylacetaldehyde**	Escherichia	coli	10.1007/s00253-013-4762-8
**phenylacetaldehyde**	Staphylococcus	epidermidis	10.3390/metabo10090347
**phenylacetaldehyde**	Staphylococcus	epidermidis	10.3390/metabo10090347
**phenylacetaldehyde**	Staphylococcus	epidermidis	10.3390/metabo10090347
**phenylacetaldehyde**	Staphylococcus	epidermidis	10.3390/metabo10090347
**phenylacetaldehyde**	Aspergillus	niger	10.1038/srep27441
**phenylacetic acid**	Staphylococcus	aureus	10.1016/j.mimet.2010.12.001
**phenylacetic acid**	Escherichia	coli	10.1016/j.mimet.2010.12.001
**phenylacetic acid**	Escherichia	coli	10.1016/j.mimet.2010.12.001
**phenylacetic acid**	Enterococcus	faecalis	10.1016/j.mimet.2010.12.001
**phenylacetic acid**	Proteus	mirabilis	10.1016/j.mimet.2010.12.001
**phenylacetic acid**	Proteus	mirabilis	10.1016/j.mimet.2010.12.001
**phenylacetic acid**	Streptococcus	pyogenes	10.1016/j.mimet.2010.12.001
**phenylacetone**	Staphylococcus	epidermidis	10.3390/metabo10090347
**phenylacetone**	Klebsiella	pneumoniae	10.1111/jam.13372
**phenylacetonitrile**	Streptococcus	agalactiae	10.1007/s00253-012-3924-4
**phenylacetonitrile**	Proteus	mirabilis	10.1007/s00253-012-3924-4
**phenylacetonitrile**	Streptococcus	pneumoniae	10.1007/s00253-012-3924-4
**phylloclad-15-ene**	Aspergillus	fumigatus	10.1128/EC.00074-14
**p-menth-8-ene, cis-**	Staphylococcus	aureus	10.1016/j.jchromb.2009.05.028
**p-mentha-1,3,8-triene**	Aspergillus	fumigatus	10.1088/1752-7155/6/1/016002
**p-mentha-1,3,8-triene**	Aspergillus	fumigatus	10.1088/1752-7155/6/1/016002
**p-mentha-1,3,8-triene**	Aspergillus	fumigatus	10.1088/1752-7155/6/1/016002
**propionaldehyde**	Pseudomonas	aeruginosa	10.1088/1752-7155/10/3/037102
**propionaldehyde**	Pseudomonas	aeruginosa	10.1088/1752-7155/10/3/037102
**propionaldehyde**	Pseudomonas	aeruginosa	10.1088/1752-7155/10/3/037102
**propionaldehyde**	Staphylococcus	aureus	10.1088/1752-7155/10/3/037102
**propionaldehyde**	Staphylococcus	aureus	10.1088/1752-7155/10/3/037102
**propionaldehyde**	Staphylococcus	aureus	10.1088/1752-7155/10/3/037102
**propionaldehyde**	Staphylococcus	aureus	10.1109/JSEN.2009.2035671

**propionaldehyde**	Staphylococcus	aureus	10.1109/JSEN.2009.2035671
**propionaldehyde**	Staphylococcus	aureus	10.1186/1471-2180-12-113
**propionaldehyde**	Burkholderia	cepacia	10.1088/1752-7155/10/3/037102
**propionaldehyde**	Burkholderia	cepacia	10.1088/1752-7155/10/3/037102
**propionaldehyde**	Burkholderia	cepacia	10.1088/1752-7155/10/3/037102
**propionaldehyde**	Haemophilus	influenzae	10.1099/mic.0.062687-0
**propionaldehyde**	Stenotrophomonas	maltophilia	10.1088/1752-7155/10/3/037102
**propionaldehyde**	Stenotrophomonas	maltophilia	10.1088/1752-7155/10/3/037102
**propionaldehyde**	Stenotrophomonas	maltophilia	10.1088/1752-7155/10/3/037102
**propionaldehyde**	Streptococcus	pneumoniae	10.1099/mic.0.062687-0
**propionic acid**	Pseudomonas	aeruginosa	10.1088/1752-7155/10/3/037102
**propionic acid**	Pseudomonas	aeruginosa	10.1088/1752-7155/10/3/037102
**propionic acid**	Pseudomonas	aeruginosa	10.1088/1752-7155/10/3/037102
**propionic acid**	Staphylococcus	aureus	10.1088/1752-7155/10/3/037102
**propionic acid**	Staphylococcus	aureus	10.1088/1752-7155/10/3/037102
**propionic acid**	Staphylococcus	aureus	10.1088/1752-7155/10/3/037102
**propionic acid**	Staphylococcus	aureus	10.3389/fmicb.2021.693075
**propionic acid**	Staphylococcus	aureus	10.3389/fmicb.2021.693075
**propionic acid**	Staphylococcus	aureus	10.3389/fmicb.2021.693075
**propionic acid**	Staphylococcus	aureus	10.3389/fmicb.2021.693075
**propionic acid**	Staphylococcus	aureus	10.3389/fmicb.2021.693075
**propionic acid**	Staphylococcus	aureus	10.3389/fmicb.2021.693075
**propionic acid**	Burkholderia	cepacia	10.1088/1752-7155/10/3/037102
**propionic acid**	Burkholderia	cepacia	10.1088/1752-7155/10/3/037102
**propionic acid**	Burkholderia	cepacia	10.1088/1752-7155/10/3/037102
**propionic acid**	Escherichia	coli	10.3389/fmicb.2021.693075
**propionic acid**	Escherichia	coli	10.3389/fmicb.2021.693075
**propionic acid**	Escherichia	coli	10.3389/fmicb.2021.693075
**propionic acid**	Escherichia	coli	10.3389/fmicb.2021.693075
**propionic acid**	Escherichia	coli	10.3389/fmicb.2021.693075
**propionic acid**	Escherichia	coli	10.3390/antibiotics9110797
**propionic acid**	Stenotrophomonas	maltophilia	10.1088/1752-7155/10/3/037102
**propionic acid**	Stenotrophomonas	maltophilia	10.1088/1752-7155/10/3/037102
**propionic acid**	Stenotrophomonas	maltophilia	10.1088/1752-7155/10/3/037102
**propyl acetate**	Candida	albicans	10.1111/myc.12442
**propyl acetate**	Escherichia	coli	10.1088/1752-7155/8/2/027106
**propyl acetate**	Escherichia	coli	10.1088/1752-7155/8/2/027106
**propyl phenylacetate**	Escherichia	coli	10.1007/s00253-012-3924-4
**propylamine, n-benzylidene-**	Escherichia	coli	10.1007/s00253-012-3924-4
**propylbenzene**	Candida	albicans	10.1038/srep27441
**propylbenzene**	Penicillium	chrysogenum	10.1038/srep27441
**propylbenzene**	Aspergillus	niger	10.1038/srep27441
**propylcyclopropane**	Aspergillus	fumigatus	10.1088/1752-7155/6/1/016002

**propylcyclopropane**	Aspergillus	fumigatus	10.1088/1752-7155/6/1/016002
**propylcyclopropane**	Aspergillus	fumigatus	10.1088/1752-7155/6/1/016002
**propylene**	Pseudomonas	aeruginosa	10.1016/j.mimet.2005.09.016
**propylene**	Escherichia	coli	10.1016/j.mimet.2005.09.016
**p-xylene**	Staphylococcus	epidermidis	10.3390/metabo10090347
**p-xylene**	Staphylococcus	epidermidis	10.3390/metabo10090347
**pyrazine**	Pseudomonas	aeruginosa	10.1038/s41598-020-74909-w
**pyrazine**	Staphylococcus	aureus	10.1038/s41598-020-74909-w
**pyrazine**	Staphylococcus	aureus	10.1038/s41598-020-74909-w
**pyrazine**	Aspergillus	fumigatus	10.1039/c8an00841h
**pyrazine**	Aspergillus	fumigatus	10.1039/c8an00841h
**pyridine**	Pseudomonas	aeruginosa	10.1128/mSphere.00843-20
**pyrimidine**	Staphylococcus	aureus	10.1128/JCM.00392-10
**pyrimidine**	Streptococcus	pneumoniae	10.3390/metabo11110773
**pyrrole**	Pseudomonas	aeruginosa	10.1007/s11306-018-1357-5
**pyrrole**	Pseudomonas	aeruginosa	10.1016/j.jchromb.2012.05.038
**pyrrole**	Pseudomonas	aeruginosa	10.1016/j.mimet.2010.12.001
**pyrrole**	Pseudomonas	aeruginosa	10.1038/s41598-020-74909-w
**pyrrole**	Pseudomonas	aeruginosa	10.1038/s41598-020-74909-w
**pyrrole**	Pseudomonas	aeruginosa	10.1088/1752-7155/10/4/047102
**pyrrole**	Pseudomonas	aeruginosa	10.1186/1471-2180-12-113
**pyrrole**	Pseudomonas	aeruginosa	10.3389/fmicb.2021.693075
**pyrrole**	Pseudomonas	aeruginosa	10.3389/fmicb.2021.693075
**pyrrole**	Pseudomonas	aeruginosa	10.3389/fmicb.2021.693075
**pyrrole**	Pseudomonas	aeruginosa	10.3389/fmicb.2021.693075
**pyrrole**	Pseudomonas	aeruginosa	10.3389/fmicb.2021.693075
**pyrrole**	Pseudomonas	aeruginosa	10.3389/fmicb.2021.693075
**pyrrole**	Pseudomonas	aeruginosa	10.3390/metabo11110773
**pyrrole**	Staphylococcus	aureus	10.1016/j.mimet.2010.12.001
**pyrrole**	Escherichia	coli	10.1016/j.mimet.2010.12.001
**pyrrole**	Escherichia	coli	10.1016/j.mimet.2010.12.001
**pyrrole**	Enterococcus	faecalis	10.1016/j.mimet.2010.12.001
**pyrrole**	Proteus	mirabilis	10.1016/j.mimet.2010.12.001
**pyrrole**	Proteus	mirabilis	10.1016/j.mimet.2010.12.001
**pyrrole**	Klebsiella	pneumoniae	10.1088/1752-7155/10/2/027101
**pyrroline**	Pseudomonas	aeruginosa	10.3390/metabo11110773
**quinazoline**	Escherichia	coli	10.1002/jssc.201800684
**rimuene**	Aspergillus	fumigatus	10.1128/EC.00074-14
**santalene**	Aspergillus	fumigatus	10.1128/EC.00074-14
**santalene**	Aspergillus	terreus	10.1093/cid/ciu725
**santalol**	Aspergillus	fumigatus	10.1128/EC.00074-14
**sesquiphellandrene**	Aspergillus	calidoustus	10.1093/cid/ciu725
**s-methyl 3-methylbutanethioate**	Pseudomonas	aeruginosa	10.3389/fmicb.2021.693075

**s-methyl 3-methylbutanethioate**	Pseudomonas	aeruginosa	10.3389/fmicb.2021.693075
**s-methyl 3-methylbutanethioate**	Pseudomonas	aeruginosa	10.3389/fmicb.2021.693075
**s-methyl 3-methylbutanethioate**	Pseudomonas	aeruginosa	10.3389/fmicb.2021.693075
**s-methyl 3-methylbutanethioate**	Pseudomonas	aeruginosa	10.3389/fmicb.2021.693075
**s-methyl 3-methylbutanethioate**	Pseudomonas	aeruginosa	10.3389/fmicb.2021.693075
**s-methyl benzenecarbothioate**	Proteus	mirabilis	10.1007/s00253-012-3924-4
**s-methyl thioacetate**	Pseudomonas	aeruginosa	10.1016/j.jchromb.2012.05.038
**s-methyl thioacetate**	Pseudomonas	aeruginosa	10.1088/1752-7155/10/1/016002
**s-methyl thioacetate**	Pseudomonas	aeruginosa	10.3389/fmicb.2021.693075
**s-methyl thioacetate**	Pseudomonas	aeruginosa	10.3389/fmicb.2021.693075
**s-methyl thioacetate**	Pseudomonas	aeruginosa	10.3389/fmicb.2021.693075
**s-methyl thioacetate**	Pseudomonas	aeruginosa	10.3389/fmicb.2021.693075
**s-methyl thioacetate**	Pseudomonas	aeruginosa	10.3389/fmicb.2021.693075
**s-methyl thioacetate**	Pseudomonas	aeruginosa	10.3389/fmicb.2021.693075
**s-methyl thioacetate**	Candida	albicans	10.3390/metabo12050432
**s-methyl thioacetate**	Candida	albicans	10.3390/metabo12050432
**s-methyl thioacetate**	Staphylococcus	aureus	10.3389/fmicb.2021.693075
**s-methyl thioacetate**	Staphylococcus	aureus	10.3389/fmicb.2021.693075
**s-methyl thioacetate**	Staphylococcus	aureus	10.3389/fmicb.2021.693075
**s-methyl thioacetate**	Staphylococcus	aureus	10.3389/fmicb.2021.693075
**s-methyl thioacetate**	Staphylococcus	aureus	10.3389/fmicb.2021.693075
**s-methyl thioacetate**	Staphylococcus	aureus	10.3389/fmicb.2021.693075
**s-methyl thioacetate**	Escherichia	coli	10.3389/fmicb.2021.693075
**s-methyl thioacetate**	Escherichia	coli	10.3389/fmicb.2021.693075
**s-methyl thioacetate**	Escherichia	coli	10.3389/fmicb.2021.693075
**s-methyl thioacetate**	Escherichia	coli	10.3389/fmicb.2021.693075
**s-methyl thioacetate**	Escherichia	coli	10.3389/fmicb.2021.693075
**s-methyl thioacetate**	Stenotrophomonas	maltophilia	10.1088/1752-7155/9/2/027104
**s-methyl thioacetate**	Stenotrophomonas	rhizophila	10.1088/1752-7155/9/2/027104
**styrene**	Candida	albicans	10.3390/metabo12050432
**styrene**	Escherichia	coli	10.1038/s41598-020-74909-w
**styrene**	Aspergillus	fumigatus	10.1039/c8an00841h
**terpinolene**	Aspergillus	fumigatus	10.1128/EC.00074-14
**terpinolene**	Aspergillus	fumigatus	10.1128/EC.00074-14
**tert-butanol**	Pseudomonas	aeruginosa	10.1016/j.jchromb.2012.05.038
**tert-butanol**	Staphylococcus	epidermidis	10.3390/metabo10090347
**tert-butyl isopropyl ether**	Staphylococcus	epidermidis	10.3390/metabo10090347
**tetradecanal**	Escherichia	coli	10.3389/fmicb.2021.693075
**tetradecane**	Pseudomonas	aeruginosa	10.1088/1752-7155/10/1/016002
**tetradecane**	Acinetobacter	baumannii	10.1088/1752-7155/10/2/027102
**tetradecane**	Escherichia	coli	10.1038/s41598-020-74909-w
**tetradecane**	Escherichia	coli	10.1038/s41598-020-74909-w
**tetradecane**	Escherichia	coli	10.1111/jam.15716

**tetrahydrofuran**	Pseudomonas	aeruginosa	10.1128/mSphere.00843-20
**thiazole**	Klebsiella	pneumoniae	10.1111/jam.13372
**thujane**	Klebsiella	pneumoniae	10.1111/jam.13372
**t-muurolol**	Streptococcus	pneumoniae	10.3390/metabo11110773
**toluene**	Pseudomonas	aeruginosa	10.1128/JCM.12.4.521-526.1980
**toluene**	Pseudomonas	aeruginosa	10.1128/JCM.12.4.521-526.1980
**toluene**	Pseudomonas	aeruginosa	10.1128/JCM.12.4.521-526.1980
**toluene**	Pseudomonas	aeruginosa	10.1128/JCM.12.4.521-526.1980
**toluene**	Pseudomonas	aeruginosa	10.1128/JCM.12.4.521-526.1980
**toluene**	Pseudomonas	aeruginosa	10.1128/JCM.12.4.521-526.1980
**toluene**	Pseudomonas	aeruginosa	10.1128/JCM.12.4.521-526.1980
**toluene**	Pseudomonas	aeruginosa	10.1128/JCM.12.4.521-526.1980
**toluene**	Pseudomonas	aeruginosa	10.1128/JCM.12.4.521-526.1980
**toluene**	Pseudomonas	aeruginosa	10.1128/JCM.12.4.521-526.1980
**toluene**	Pseudomonas	aeruginosa	10.1128/JCM.12.4.521-526.1980
**toluene**	Candida	albicans	10.1038/srep27441
**toluene**	Penicillium	chrysogenum	10.1038/srep27441
**toluene**	Escherichia	coli	10.3390/antibiotics9110797
**toluene**	Escherichia	coli	10.3390/antibiotics9110797
**toluene**	Aspergillus	niger	10.1038/srep27441
**trans-2-butene**	Streptococcus	pneumoniae	10.1099/mic.0.062687-0
**trans-2-heptenal**	Aspergillus	fumigatus	10.1088/1752-7155/6/1/016002
**trans-2-hexen-1-ol**	Pseudomonas	aeruginosa	10.1128/mSphere.00843-20
**trans-2-hexen-1-ol**	Pseudomonas	aeruginosa	10.1186/s13568-022-01367-0
**trans-2-octenal**	Pseudomonas	aeruginosa	10.1016/j.jchromb.2012.05.038
**trans-2-octenal**	Pseudomonas	aeruginosa	10.1128/mSphere.00843-20
**trans-2-octenal**	Pseudomonas	aeruginosa	10.1186/s13568-022-01367-0
**trans-2-undecenal**	Candida	albicans	10.1038/srep27441
**trans-2-undecenal**	Penicillium	chrysogenum	10.1038/srep27441
**trans-2-undecenal**	Aspergillus	niger	10.1038/srep27441
**tridecanal**	Escherichia	coli	10.1111/jam.15716
**tridecane**	Pseudomonas	aeruginosa	10.1038/s41598-020-74909-w
**trimethylamine**	Pseudomonas	aeruginosa	10.1016/j.mimet.2010.12.001
**trimethylamine**	Pseudomonas	aeruginosa	10.1016/j.mimet.2010.12.001
**trimethylamine**	Staphylococcus	aureus	10.1016/j.mimet.2005.09.003
**trimethylamine**	Staphylococcus	aureus	10.1016/j.mimet.2010.12.001
**trimethylamine**	Escherichia	coli	10.1016/j.mimet.2005.09.003
**trimethylamine**	Escherichia	coli	10.1016/j.mimet.2010.12.001
**trimethylamine**	Escherichia	coli	10.1016/j.mimet.2010.12.001
**trimethylamine**	Enterococcus	faecalis	10.1016/j.mimet.2010.12.001
**trimethylamine**	Proteus	mirabilis	10.1016/j.mimet.2010.12.001
**trimethylamine**	Proteus	mirabilis	10.1016/j.mimet.2010.12.001
**trimethylamine**	Streptococcus	pneumoniae	10.1016/j.mimet.2005.09.003

**tropinone**	Stenotrophomonas	maltophilia	10.3390/metabo11110773
**undec-3-ene**	Staphylococcus	aureus	10.3390/metabo10090347
**undec-3-ene**	Staphylococcus	epidermidis	10.3390/metabo10090347
**undecanal**	Escherichia	coli	10.1111/jam.15716
**undecanal**	Escherichia	coli	10.1111/jam.15716
**undecane**	Pseudomonas	aeruginosa	10.1016/S0378-4347(00)80760-4
**undecane**	Pseudomonas	aeruginosa	10.1016/S0378-4347(00)80760-4
**undecane**	Staphylococcus	epidermidis	10.3390/metabo10090347
**undecane**	Staphylococcus	epidermidis	10.3390/metabo10090347
**undecane**	Klebsiella	pneumoniae	10.1016/S0378-4347(00)80760-4
**undecane**	Klebsiella	pneumoniae	10.1016/S0378-4347(00)80760-4
**undecylenic acid**	Streptococcus	pneumoniae	10.3390/metabo11110773
**valeraldehyde**	Pseudomonas	aeruginosa	10.1088/1752-7155/10/3/037102
**valeraldehyde**	Pseudomonas	aeruginosa	10.1088/1752-7155/10/3/037102
**valeraldehyde**	Pseudomonas	aeruginosa	10.1088/1752-7155/10/3/037102
**valeraldehyde**	Staphylococcus	aureus	10.1088/1752-7155/10/3/037102
**valeraldehyde**	Staphylococcus	aureus	10.1088/1752-7155/10/3/037102
**valeraldehyde**	Staphylococcus	aureus	10.1088/1752-7155/10/3/037102
**valeraldehyde**	Burkholderia	cepacia	10.1088/1752-7155/10/3/037102
**valeraldehyde**	Burkholderia	cepacia	10.1088/1752-7155/10/3/037102
**valeraldehyde**	Burkholderia	cepacia	10.1088/1752-7155/10/3/037102
**valeraldehyde**	Stenotrophomonas	maltophilia	10.1088/1752-7155/10/3/037102
**valeraldehyde**	Stenotrophomonas	maltophilia	10.1088/1752-7155/10/3/037102
**valeraldehyde**	Stenotrophomonas	maltophilia	10.1088/1752-7155/10/3/037102
**valeraldehyde**	Streptococcus	pneumoniae	10.1016/j.jchromb.2018.08.032
**valeraldehyde**	Streptococcus	pneumoniae	10.1016/j.jchromb.2018.08.032
**valeraldehyde**	Streptococcus	pneumoniae	10.1016/j.jchromb.2018.08.032
**valeraldehyde**	Streptococcus	pneumoniae	10.1016/j.jchromb.2018.08.032
**valeraldehyde**	Streptococcus	pneumoniae	10.1016/j.jchromb.2018.08.032
**valeraldehyde**	Streptococcus	pneumoniae	10.1016/j.jchromb.2018.08.032
**valeraldehyde**	Streptococcus	pneumoniae	10.1016/j.jchromb.2018.08.032
**valeric acid**	Pseudomonas	aeruginosa	10.1088/1752-7155/10/3/037102
**valeric acid**	Pseudomonas	aeruginosa	10.1088/1752-7155/10/3/037102
**valeric acid**	Pseudomonas	aeruginosa	10.1088/1752-7155/10/3/037102
**valeric acid**	Staphylococcus	aureus	10.1088/1752-7155/10/3/037102
**valeric acid**	Staphylococcus	aureus	10.1088/1752-7155/10/3/037102
**valeric acid**	Staphylococcus	aureus	10.1088/1752-7155/10/3/037102
**valeric acid**	Burkholderia	cepacia	10.1088/1752-7155/10/3/037102
**valeric acid**	Burkholderia	cepacia	10.1088/1752-7155/10/3/037102
**valeric acid**	Burkholderia	cepacia	10.1088/1752-7155/10/3/037102
**valeric acid**	Stenotrophomonas	maltophilia	10.1088/1752-7155/10/3/037102
**valeric acid**	Stenotrophomonas	maltophilia	10.1088/1752-7155/10/3/037102
**valeric acid**	Stenotrophomonas	maltophilia	10.1088/1752-7155/10/3/037102
